# Optimization of a Novel Mandelamide-Derived Pyrrolopyrimidine Series of PERK Inhibitors

**DOI:** 10.3390/pharmaceutics14102233

**Published:** 2022-10-19

**Authors:** Michael E. Stokes, Matthew D. Surman, Veronica Calvo, David Surguladze, An-Hu Li, Jennifer Gasparek, Matthew Betzenhauser, Guangyu Zhu, Hongwen Du, Alan C. Rigby, Mark J. Mulvihill

**Affiliations:** 1 HiberCell Inc., 619 West 54th Street, New York, NY 10019, USA; 2 Curia, 26 Corporate Circle, Albany, NY 12203, USA; 3 Curia, 1001 Main Street, Buffalo, NY 14203, USA; 4 Pharmaron Beijing Co., Ltd., No. 6, TaiHe Road, BDA, Beijing 100176, China

**Keywords:** ER stress, PERK, UPR, kinase, inhibitor, cancer, diabetes, small molecule, structure–activity-relationship (SAR)

## Abstract

The protein kinase R (PKR)-like endoplasmic reticulum kinase (PERK) is one of three endoplasmic reticulum (ER) transmembrane sensors of the unfolded protein response (UPR) responsible for regulating protein synthesis and alleviating ER stress. PERK has been implicated in tumorigenesis, cancer cell survival as well metabolic diseases such as diabetes. The structure-based design and optimization of a novel mandelamide-derived pyrrolopyrimidine series of PERK inhibitors as described herein, resulted in the identification of compound **26**, a potent, selective, and orally bioavailable compound suitable for interrogating PERK pathway biology in vitro and in vivo, with pharmacokinetics suitable for once-a-day oral dosing in mice.

## 1. Introduction

Cells must match the rates of protein synthesis with demand to avoid the onset of proteotoxic endoplasmic reticulum (ER) stress. In conditions of excess protein production, an accumulation of misfolded proteins in the ER lumen induces the unfolded protein response (UPR), which arrests global translation while enabling specific processes that restore protein homeostasis [1,2]. The UPR is mediated by three discrete signaling cascades, IRE1, ATF6, and EIF2AK3 (PERK) [3]. Upon activating the UPR, PERK phosphorylates eIF2a at Ser51, leading to widespread inhibition of protein translation with a few selected regulatory proteins translated to restore homeostasis and alleviate ER stress [1,3]. In cancer, acute activation of UPR enables tumors to overcome deleterious ER stress induced by rapid cell proliferation, nutrient deprivation, hypoxia, and drug treatments [4,5,6]. Pharmacological or genetic inhibition of PERK slows proliferation of tumor xenografts in mice [7,8], and small molecule PERK inhibitors have recently entered clinical trials for several oncology indications, including clear cell renal cell carcinoma (ccRCC) and multiple myeloma (NCT04834778; NCT05027594).

Acute ER stress and activation of UPR restores protein homeostasis and supports cell survival, whereas long-term ER stress shifts cells toward an apoptotic cell fate [9]. While intermittent ER stress occurs regularly in cell types that produce high abundance of protein, metabolic dysfunction can result in chronic ER stress that coincides with a number of metabolic and neurological diseases [9,10,11,12]. Type-1 Diabetes (T1D) is an example of a metabolic disease and autoimmune disorder characterized by PERK hyperactivation in insulin-producing β-cells of the pancreatic islets [11,12,13]. Immunohistochemistry (IHC) staining of pancreas sections from T1D patients revealed PERK pathway activation in insulin-secreting beta cells, relative to healthy control patient samples [13], and PERK activation precedes the destructive autoimmune attack of beta cells in animal models of T1D [12,14]. Together, these findings highlight a potential role for PERK in diabetes and possible therapeutic benefit of PERK inhibitors beyond oncology.

While some cancers and metabolic diseases are characterized by PERK hyperactivation, genetic ablation of PERK results in glucose dysregulation and pancreatitis, suggesting that some basal level of PERK activity is essential to pancreatic health [15,16]. It is possible that the attenuation of PERK activity, rather than complete suppression, could provide therapeutic benefit while supporting normal pancreatic function. To enable future testing of this hypothesis, we sought to create a series of PERK inhibitors with stable PK profiles that could be administered at low doses to attenuate PERK yet tuned through higher doses to sufficiently inhibit PERK for tumor inhibition.

Previously described medicinal chemistry efforts around PERK inhibitors have made use of a variety of core hinge-binding regions, including aminopyridines, pyrrolopyrimidines, quinazolines, and quinolines [7,8,17]. Pyrrolopyrimidine cores have been developed and approved as JAK kinase inhibitors [18,19], and modifications to the core scaffold can have profound effects on stability and selectivity [17]. Here, we optimized the pyrrolopyrimidine core and leveraged extensive SAR information from our recently reported aminopyridine series of inhibitors to guide chemistry efforts aimed at optimizing the in vivo properties of pyrrolopyrimidine PERK inhibitors [7].

Our efforts were focused on developing potent and selective PERK inhibitors with suitable pharmacokinetic profiles to enable once or twice per day oral dosing in mouse models with a flat steady state exposure profile. The goal was to avoid large differences between C_max_ to C_12h_ or C_24h_ in order to achieve a constant and sustained pharmacodynamic response. This paper focuses on the structure-based design, structure–activity relationship (SAR), absorption, distribution, metabolism and excretion (ADME), and pharmacokinetic/pharmacodynamic (PK/PD) properties of a novel class of mandelamide-derived pyrrolopyrimidine PERK inhibitors leading to the identification of Compound **26,** with the desired flat steady state exposure profile. Compound **26** was characterized in a mouse pancreas assay and has been selected for further evaluation in animal models, which will be reported in due course.

## 2. Materials and Methods

### 2.1. Biochemical Assays

The inhibitory potencies of **26** inhibition against PKR, HRI, PERK and GCN2 were evaluated in cell-free biochemical assays following manufacturer’s instructions (Carna; Table 1). In brief, **26** was dissolved in DMSO and serially diluted 3-fold to generate concentrations ranging from 10 µM to 0.2 nM in a 384-well plate using a TECAN EVO200 automated liquid handler. 10 nL stock solution was transferred into each well of a 384well assay plate by an Echo550 and an equivalent volume of DMSO was added to the control wells. To each assay well, recombinant protein was suspended in 20 µL reaction buffer (listed below) and incubated at 25 °C for 1.5 h while shaking at 1250 rpm. Following incubation, 10 µL of a FRET dilution buffer including 20 mM EDTA and 4 nM Tb-anti-peIF2a [pSer51] (Invitrogen, Cat# PV4815) was added to each well and incubated at 25 °C for 2 h while shaking at 600 rpm in the dark. Analysis was performed using an Envision Microplate Reader (PerkinElmer).

### 2.2. PERK Crystallization and Structure Determination

Human PERK (575-1094 Δ670-874) was purified as described previously [7]. Purified PERK protein at 11 mg/mL was mixed with 10 mM compound **11**, **24**, or **26** (in DMSO) to a final protein-inhibitor molar ratio 1:2. The PERK-inhibitor mixture was incubated on ice for 2 h before crystallization. The crystals with compound **11** or **26** were grown at 20 °C in sitting drops by combining 2.0 µL PERK-inhibitor mixture, 2.0 µL reservoir solution (12–14% PEG3350, 4% tacsimate pH7.0), and 0.4 µL seed stock which was equilibrated over a 500 µL reservoir solution. The crystal with compound **24** was grown at 4 °C in a hanging drop by combining 1.5 μL PERK-inhibitor mixture, 1.5 μL reservoir solution (9% PEG3350, 180 mM Na/K tartrate, 100 mM HEPES pH 7.0) which was equilibrated over a 500 μL reservoir solution. The crystals were grown to 0.2–0.5 mm over a three-week period before harvesting for analysis. The crystal was transferred stepwise to a cryo-solution with the crystallization cocktail plus 20% glycerol before being frozen in liquid nitrogen. Diffraction data were collected at the IMCA-CAT beamline 17ID for compound **11** or GMCA-CAT beamline 23IDD for compound **24** or **26** at the Advanced Photon Source at Argonne National Laboratory using a Pilatus 6M detector. The diffraction images were processed with DIALS [20] and scaled with AIMLESS [21]. The structure was solved by molecular replacement using PDB 4X7J [17] as starting model by Phaser [22]. The structure was manually built using Coot [23] and subsequently refined using Refmac5 [24]. The crystallographic figures were generated by CCP4MG [25], and the statistics of data collection and refinement are summarized in Supplemental Table S1.

### 2.3. Pharmacokinetic and Pharmacodynamic Analysis in Plasma and Pancreas

For PK analysis of pyrrolopyrimidine analogs in mouse, rat, and dog plasma, the oral formulation was prepared as a solution containing 20% (*w*/*v*) Captisol in 25 mM NaH_2_PO_4_ buffer (pH 2). Compounds were administered orally by gavage at 10 mg/kg to female CD1 mice. After dosing, plasma samples were collected to characterize the PK profiles at 0.25, 0.5, 1, 2, 4, 8, 12 and 24 h post-dose. The plasma concentration of compound was determined by protein precipitation with acetonitrile and liquid chromatography with mass spectrometric detection (LC-MS/MS). Parameters were estimated using Phoenix (WinNonlin) pharmacokinetic software version 6.1.0 using a non-compartmental approach consistent with the oral route of administration. PK analysis of **26** in plasma and pancreas followed a similar analytical method as above, with minor modifications. Compound **26** was suspended in a vehicle consisting of 0.5% methylcellulose (400 cP) and 0.1% Tween80 in water and administered to BALB/c nude mice by oral gavage at 0.3, 1, 3, 10, 30 mg/kg. Plasma and pancreas were sampled from 5 mice per group following a single oral administration at 1, 4, 8, 12, 24 h post-dose.

Total protein was extracted from pancreas tissues sampled during PK studies described above. Frozen pancreas tissue was homogenized by polytron in lysis buffer consisting of 2x Laemmli SDS sample buffer (Novex), supplemented with 10% BME (Gibco), 1X benzonase (EMD Millipore Sigma), phosphatase inhibitors (Sigma) and Mini protease inhibitor tablet (Roche). Homogenate was incubated at room temperature for 10′, then boiled for 10′, followed by centrifugation for 10′ at max speed on a benchtop centrifuge. Protein analysis performed using JESS high-throughput protein analysis instrument (Biotechne) using antibodies for phosphoPERK developed internally and PERK (CST).

### 2.4. Biochemical and Cell-Based Characterization of Pyrrolopyrimidine Analogs

Methods used to evaluate biochemical PERK activity are described and published elsewhere [7]. In brief, biochemical PERK activity is evaluated using a LanthaScreenTM (PerkinElmer) TR-FRET assay to detect phosphorylation of a GFP-tagged eIF2a substrate (ThermoFisher). Excitation at 340 nM of a terbium chelate donor fluorophore in an eIF2a antibody results in energy transfer to GFP acceptor fluorophore upon eIF2a phosphorylation by PERK. Reaction buffer was composed of 50 mM HEPES (pH 7.4), 10 mM MgCl_2_, 1 mM EGTA and 0.01% (*v*/*v*) BrijTM-35. Reactions were initiated by the addition of substrate and ATP, followed by incubation for 1 h at RT prior to addition of EDTA and the anti-peIF2a antibody. FRET was measured by an EnVision Plate Reader (PerkinElmer). Data were plotted as percent inhibition along a 10-point 3-fold dilution series of inhibitors. IC_50_ values were calculated using 4-parameter logistical fitting in XLFit.

Methods used to evaluate in vitro cellular PERK inhibition are described and published elsewhere [7]. In brief, stableHEK293 cells were created using lentiviral particles harboring a GFP-eIF2a expression vector. Transfectants were selected by puromycin and enriched using fluorescent cell sorting based on GFP expression. HEK293-EGFP-eIF2a cells were plated at 5000 cells/well in 384-well assay plates and incubated overnight. The following day, pyrrolopyrimidine analogs were added to wells using an Echo acoustic dispenser and incubated for 30 min. ER stress was induced by the addition of tunicamycin to the wells at 1 mM for 2 h. Cells were lysed and peIF2a was evaluated using a TR-FRET system, as described above.

### 2.5. In Vivo Tumor Xenograft Studies

In vivo studies of the effect of pyrrolopyrimidines on 786-O xenograft inhibition follow a method published elsewhere [7]. Female BALB/c nude mice were inoculated subcutaneously with 786-O tumor cells (5 × 10^6^) in 0.1 mL of PBS. Animals were randomized when tumors reached 100–200 mm^3^ into treatment groups. Pyrrolopyrimidine PERK inhibitors **24**, **26** or **70** were dissolved in vehicle solution consisting of 20% (*w*/*v*) Captisol in 25 mM NaPO_4_ buffer (pH2) and administered twice daily (BID) by oral gavage for 29 days. Tumor volumes were measured by caliper and the volume was expressed in mm^3^ using the formula: V = (L × W × W)/2, where V, L, and W represent tumor volume, length, and width, respectively. All studies were conducted following an IACUC-approved protocol (AN-1903-05-1798). Experimental data management and reporting were in accordance with applicable Crown Bioscience’s Guidelines and Standard Operating Procedures.

## 3. Results and Discussion

Small molecule PERK inhibitors across a variety of chemotypes have been previously described in the literature and captured in an earlier publication from our group around an aminopyridine series of PERK inhibition [7]. Herein, we report the synthesis and discovery of a series of novel mandelamide-derived pyrrolopyrimidines (I) that are highly potent, selective, and orally bioavailable PERK inhibitors. These medicinal chemistry efforts were iteratively supported by an X-ray crystallography structure-based approach to develop and refine SAR information. Collectively this approach permitted us to optimize the physiochemical and ADME/PK properties of this chemical series. Different R^1^, R^2^, R^3^, Ar and L groups have been introduced into our PERK small molecule series to tune the potency, selectivity and drug-like properties, such as aqueous solubility and cellular permeability.

**Scheme 1:**
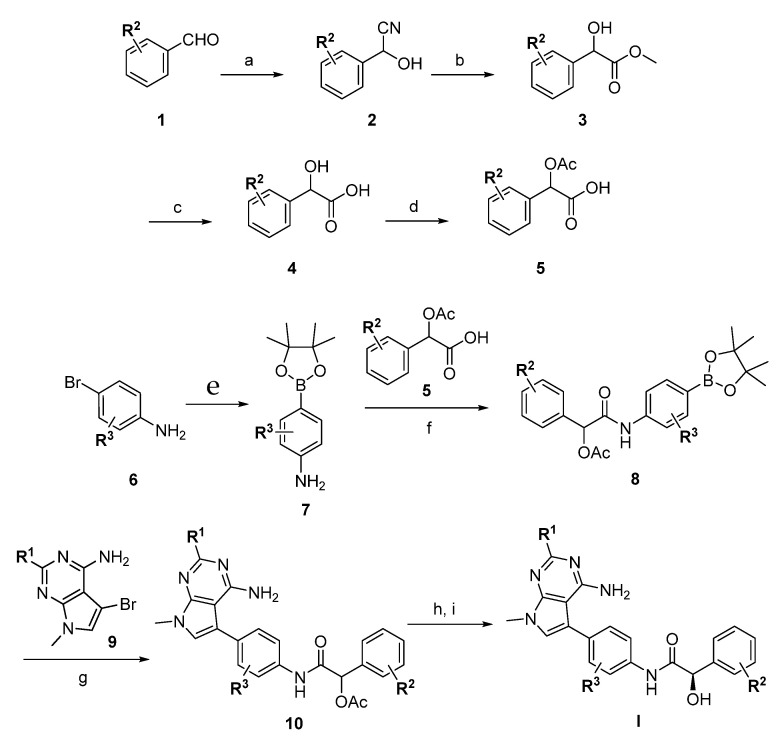
General synthesis of PERK inhibitors. Reaction conditions: (a) TMS-CN (1.2 equiv.), Znl_2_ (0.1 equiv.), 0 °C; (b) 3M HCl in MeOH, 0 °C to rt, 60–80% over two steps; (c) LiOH•H_2_O (1.2 equiv.), THF, H_2_O, MeOH, rt, 50–85%; (d) AcCl (2 vol.), 0 °C to rt, 70–85%; (e) B_2_pin_2_ (1.2 equiv.), KOAc (3 equiv.), PdCl_2_ (PPh_3_) _2_ (5 mol. %), NEt_3_ (10 vol.), 90 °C, 50–70%; (f) **5** (1.2 equiv.), 50% propylphosphonic anhydride (T_3_P) in EtOAc (1.5 equiv.), DIPEA (3 equiv.), CH_2_Cl_2_, 0 °C to rt, 50–80%; (g) XPhos-Pd-G_2_ (10 mol. %), K_2_CO_3_ (3 equiv.), THF, H_2_O, 80 °C, 25–70%; (h) K_2_CO_3_ (2 equiv.), MeOH, rt, 80–90%; (i) Chiral SFC separation.

The general synthesis for our pyrrolopyrimidine PERK inhibitors is shown in Scheme 1. The terminal ring substitution with different R^2^ groups was introduced through the corresponding aldehyde **1**. Cyanohydrin **2** formation with TMS-cyanide and zinc(II) iodide followed by acid hydrolysis of the nitrile in methanol provided methyl mandelic ester **3**. Hydrolysis of the ester with lithium hydroxide and acylation of the benzylic alcohol gave the protected mandelic acid **5**.

The proximal ring substitution with different R^3^ groups was introduced through bromo aniline **6**. Palladium-mediated borylation of **6** to give the aniline boronate ester **7** was followed by T_3_P coupling with acid **5** to provide amide **8**. Boronate ester **8** underwent Suzuki coupling with pyrrolopyrimidine bromide **9** to give racemic acetyl-protected product **10**. Deacylation under basic conditions followed by chiral separation of the enantiomers provided the final product **I**.

The PERK activity of analog **I** was evaluated using cell-free biochemical assays. In these assays, PERK phosphorylation of eIF2a was assessed using a FRET-based labelled protein assay [7]. Compounds were then evaluated for cellular activity by quantifying inhibition of eIF2a phosphorylation in HEK293 cells treated with the ER inducer tunicamycin [7]. Together, these data read out the potency of a given analog against PERK as well as the cellular permeability to enable bioactivity.

Initial SAR studies in the pyrrolopyrimidine series focused on the linker (**L**) region between the central amide and terminal aryl ring (Table 2). Hydroxyl substitution on this group was found to increase potency by 2-3-fold (**13** vs. **14**) with preference for the (*R*)-over the *(S)*-enantiomer (**11** vs. **12**). Expansion or contraction of the length of the linker **L** was not tolerated, resulting in at least 20-fold losses in PERK biochemical potency and nearly 50- to 100-fold losses in cellular potency (**14** vs. **15** and **16**, respectively). Difluoro substitution of the linker also caused a substantial loss in potency, as did replacement of the CH(OH) group with an O, NH, or NCH_3_ group (**13** vs. **17**, **18**, **19**, and **20**, respectively). Seven-fold losses in both biochemical and cellular potency were observed when the hydroxyl group was converted to the methyl ether (**13** vs. **21**). Even more substantial losses were associated with replacement of the hydroxyl with an amino group (**13** vs. **23**). Addition of a methyl group to the linker to form a tertiary alcohol also caused a substantial loss in potency (**13** vs. **22**). Based on these findings, it was concluded that (R)-CH(OH) is the preferred **L** group.

**Figure 1:**
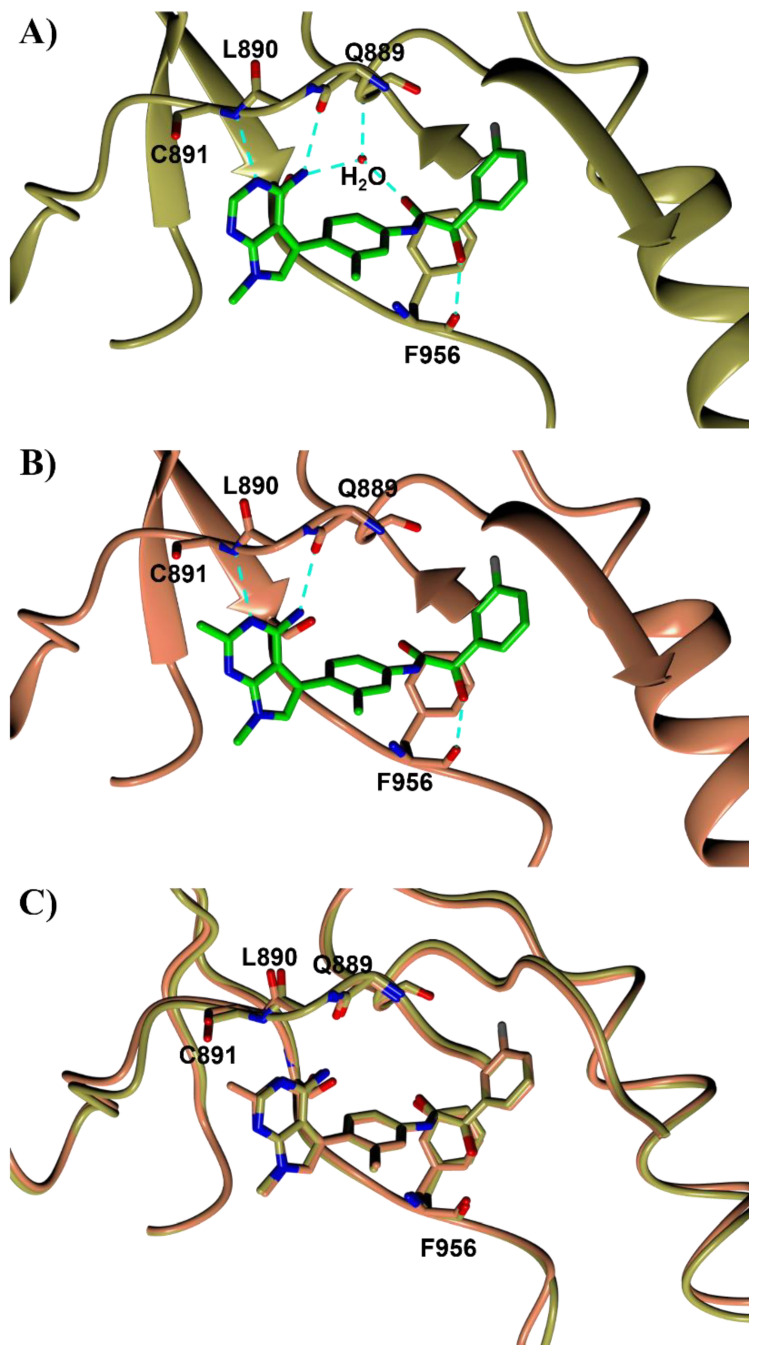
Crystal structures of PERK with selected inhibitors **11** (**A**) and **24** (**B**). Overlay of the crystal structures of **11** and **24** (**C**). The same orientation is presented for all panels. The PERK backbone is shown as a ribbon or solid strand in yellow for **11** and coral for **24**. The side chains are depicted as cylinders in the same color as the main chain for carbon, blue for nitrogen, and red for oxygen atoms. Inhibitors are shown as cylinders with same color scheme except green for carbon in panels (**A**,**B**) and grey for fluorine. Hydrogen bonds are shown as cyan dashed lines.

Previously reported SAR of PERK inhibitors have revealed modifications to the hinge binding region that have profound effects on binding affinity to PERK as well as selectivity versus the kinome [7,17]. Structure based design modelling was used to focus efforts on optimizing the pyrrolopyrimidine hinge-binding region of the molecule. Inhibitor **11** was co-crystallized with PERK into a trigonal space groupP3_2_21 with comparable cell parameters as PDB 4X7O. Compound **11** formed three hydrogen bonds directly with the carbonyl of Q889, the amide of C891, and the carbonyl of F956 from PERK, respectively (Figure 1A). In addition, compound **11** forms two hydrogen bonds with a water molecule, and this molecule hydrogen bonds to the amide of V652. In the ATP binding hinge region, substitution at the C2-position (R1) of the pyrrolopyrimidine ring appeared feasible, filling a small pocket near C891. A methyl group located at the R1 position of compound **11** afforded analog **24** whose co-crystal structure with PERK confirmed the occupation of the small pocket near C891 along with the close contact of the C2-methyl group with the carbonyl of C891 with a distance of 3.14 Å (Figure 1B). Although a weak CH···O hydrogen bond is feasible, this contact appears to be more repulsive. As a result, a small rotation of the main chain amino acid Q889 and L890 of PERK, along with a minor shift of the pyrrolopyrimidine ring of inhibitor **24** are observed compared to those in the structure of **11** (Figure 1C). These shifts maintain the hydrogen bonds with similar distances. Due to a lower resolution (2.92Å) of the compound **24** structure (Table S1), the aforementioned water molecule in the compound **11** structure was not observed. This water molecule is assumed to locate at the same position, as all hydrogen bond doner and acceptor atoms are still presented at similar positions in the compound **24** structure. These observations indicate that the reduction of potency of **24** is likely caused by repulsion from the methyl group rather than the disruption of the hydrogen bonding pattern.

**Figure 2:**
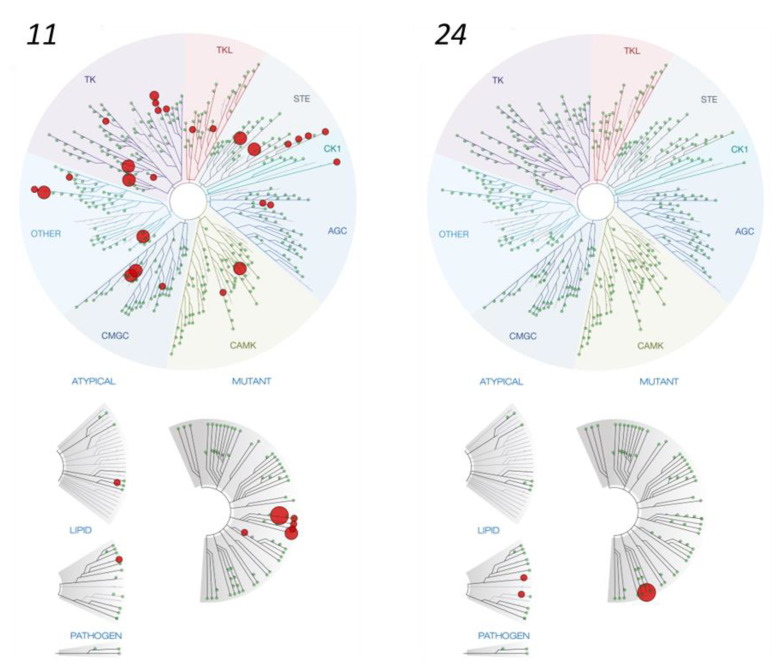
The TREEspot^TM^ interaction map of **11** and **24** assayed at 1000 nM in the 468-kinase scanMAXSM panel. The panel includes 403 unique wild-type kinases and 59 mutant isoforms. Note that PERK is not included in the TREEspot assay but was evaluated in separate biochemical assays. Complete tabulated results presented in Supplemental Table S2.

While having a minor effect on PERK potency, the methyl substituent on the pyrrolopyrimidine ring had a profound effect on the kinase selectivity. Compounds **11** and **24** were screened in the DiscoverX scanMAX^SM^ panel of 468 kinases [26]. The resulting TREEspot^TM^ interaction maps (Figure 2) revealed that the methyl substituent in **24** led to superior selectivity versus **11**. The S(35), S(10), and S(1) selectivity scores at a concentration of 1 µM were 0.005, 0, and 0 for **24** versus 0.072, 0.035, and 0 for **11**. Given the superior selectivity of **24** but slight loss in potency versus **11**, further optimization of the series was carried out with the 2-methyl substitution on the pyrrolopyrimidine ring to further improve potency and drug-like properties.

Exploration of the terminal aryl ring revealed several trends (Table 2): (1) Substitution at the 3-position was preferred to substitution at the 2-position of the ring (compare **24** vs. **25**, **26** vs. **27**, **28** vs. **29**, and **30** vs. **31**). (2) Lipophilic substituents, such as CF_3_, Cl, Br, CCH, CF_2_H, and *c*-Pr provided enhanced biochemical and cellular potency compared with F substitution (**24** vs. **26**, **28**, **33**, **32**, **34**, and **35**) leading to the recovery of the potency lost with the incorporation of the selectivity-enhancing 2-methyl pyrrolopyrimidine ring (compare **11** vs. **24** and **11** vs. **26**, **28**, **33**, and **32**). (3) Polar substituents were not well tolerated and resulted in substantially reduced cellular potency, as in the case of CN derivative **36**, or in complete loss of activity (biochemical and cellular activity at the concentrations tested), as in the case of sulfone analog **37**. (4) 3,5-Di-substitution with lipophilic groups provided further enhancements in potency (**38**, **39**, **40**, and **41**). Interestingly, addition of a fluorine substituent to CN on the terminal phenyl ring largely reversed the loss of cellular potency due to the CN group (**36** vs. **42**). (5) Replacement of the terminal phenyl ring with heterocycles, such as F-, Cl-, or CF_3_-substituted pyridines, resulted in 8- to 11-fold losses in cellular potency (compare **24** vs. **43**, **28** vs. **44**, and **26** vs. **45**).

Next, the SAR of the proximal aryl ring was examined with new analogs incorporating the best substituted phenyl groups from the survey of the terminal aryl ring (3-F-Ph; 3-CF_3_-Ph; 3-Cl-Ph; 3-CH_3_-Ph; 3-Et-Ph; 3-F,5-CF_3_-Ph; and 3-F,5-CH_3_-Ph) was (Table 3). Replacement of the proximal ring methyl substituent with a F, Cl, or Et group was found to be well tolerated (**24** vs. **46**, **53**, and **59**). These changes to the proximal ring could be combined with modifications to the terminal ring to provide analogs with <5 nM biochemical and <15 nM cellular potency (**47**, **48**, **52**, **57**). Modest losses in cellular potency (2.5- to 4-fold) were observed for H and CF_3_ methyl replacements on the proximal ring (**24** vs. **62** and **58**). More substantial reductions in potency (8- to 36-fold) occurred with CF_3_O and CH_3_O replacements (**24** vs. **61** and **60**). Addition of a fluorine substituent to the proximal ring of **24** resulted in the general maintenance of cellular potency with a 10-fold range in biochemical potency, depending upon the F regioisomer (**24** vs. **65**, **66**, and **67**). 3,5-Dimethyl proximal ring substitution (**68**) provided comparable in vitro potency to the 3-fluoro, 5-methyl analog **67**. Movement of the fluorine substituent from the 3-position of the proximal ring (**46**) to the 2-position (**69**) had little impact on the biochemical or cellular potency of the compounds. Addition of a second fluorine to the proximal ring of **46** similarly had little impact on the cellular potency of the compounds, although some variation in the biochemical data was observed (**46** vs. **70** and **71**). Replacement of the proximal phenyl ring of **24** with a pyridine ring resulted in modest (2- to 3-fold) losses in cellular potency (**24** vs. **72** and **74**). Some of the lost potency could be restored by replacing the terminal ring F with CF_3_ (**73** vs. **72** and **75** vs. **74**). Replacement of the proximal phenyl ring of **24** with a pyrimidine ring was not tolerated, resulting in a 30-fold loss in biochemical potency and complete loss in cellular activity (**24** vs. **76**).

The SAR campaign successfully identified a number of PERK inhibitors that demonstrated biochemical and cellular potency. These promising compounds were evaluated in in vitro ADME assays (aqueous solubility, Caco-2 permeability, plasma protein binding, and hepatocyte stability) to determine their drug-like properties (Table 4). In general, the compounds showed moderate kinetic aqueous solubility in PBS at pH 7.4, ranging from 10–66 µM. The Caco-2 permeability was found to be high for all compounds (P_app_ ≥ 9 × 10^−6^ cm/s), predicting good oral absorption. Additionally, with the exception of **24** (efflux ratio of 5.2), the compounds showed little to no efflux. Plasma protein binding was found to be high across all species tested, a characteristic common for many kinase inhibitors.

In vitro metabolic stability was assessed in human, rat, mouse, and dog hepatocytes. In general, the compounds were found to be relatively stable in human and dog hepatocytes and exhibited long half-lives. For several compounds (**28**, **65**, **24**, **41**, **66**, and **70**), stability was reduced in rodent hepatocytes, particularly rat. Compounds with alkyl groups (i.e., Me) on the terminal phenyl ring tended to have lower stability (**41** vs. **39**). Several compounds (**26**, **39**, and **38**) maintained good metabolic stability across all species tested.

To connect the in vitro ADME results with the in vivo setting, select compounds (**26**, **28**, **65**, **24**, **41**, **39**, **38**, **48**, **66**, and **70**) were examined in mouse PK studies (Table 5). All of the compounds showed robust oral exposure with C_max_ values for most compounds >8000 ng/mL and AUC_0–last_ values >25,000 h·ng/mL. The compounds also showed good in vivo half-lives of 2–3.5 h and excellent bioavailability (≥70%) apart from compound **41**. Compound **26** stood out with a particularly attractive pharmacokinetics profile with C_max_ of 17,200 ng/mL, AUC_0–last_ of 126,019 h·ng/mL, half-life of 3.5 h, and bioavailability of 82%, a significant improvement over our earlier analog **24** and was therefore chosen as one of the key leads in this series PERK inhibitors.

**Figure 3:**
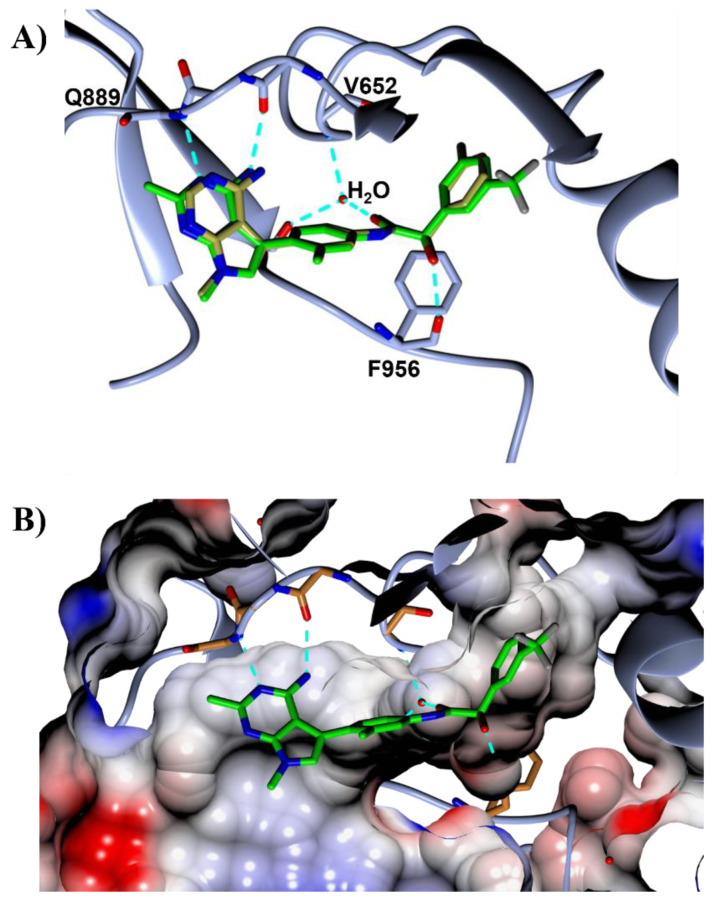
Crystal structure of compound **26** bound to the PERK kinase domain. (**A**) View of **26** in the PERK active site. PERK backbone is shown as a blue ribbon, and the side chains are shown as cylinder in brown for carbon, blue for nitrogen, and red for oxygen atoms, respectively. Compound **26** is shown as cylinder with same color scheme except green for carbon and grey for fluorine. Compound **11** is shown in a similar manner to compound **26**, except the color gold is used for carbon atoms. Hydrogen bonds for **26** are shown as cyan dashed lines. The phenyl ring is flipped due to a replacement of the 3-fluorine in **11** to CF_3_ in **26**. (**B**) View of **26** in the PERK active site with surface rendering. The color intensity corresponds to the calculated electrostatic potential, from −20 kT/e (red) to +20 kT/e (blue).

Inhibitor **26** was co-crystallized with PERK into a trigonal space groupP3_2_21 with comparable cell parameters as previously noted with compounds **11** and **24**. Replacement of the 3-fluorine on the terminal phenyl group in **11** and **24** and with a larger CF_3_ group in **26** induces a flip in the phenyl ring since the enzyme position containing the fluorine cannot accommodate the bulkier CF_3_ group (Figure 1 and Figure 3). The fluorine atom of **24** forms four contacts with a distance of 3.8 Å or less with residues within the back pocket region of PERK, whereas the CF_3_ group of **26** forms ten contacts with PERK. These extra contacts may stabilize the binding of **26** to PERK and explain the increased potency of **26** compared to **24**.

**Figure 4:**
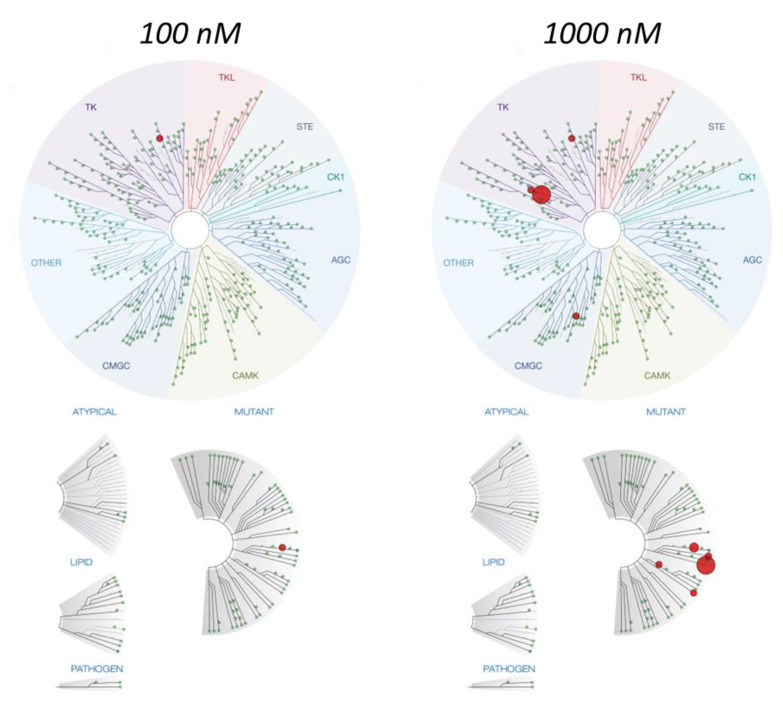
The TREEspot^TM^ interaction map of compound **26** in the 468-kinase scanMAXSM panel at 100 and 1000 nM. The panel includes 403 unique wild-type kinases and 59 mutant isoforms. Note that PERK is not included in the TREEspot assay. Complete tabulated results presented in Supplemental Table S2.

Compound **26** was found to have excellent selectivity in the DiscoverX kinase panel (Figure 4; Supplemental Table S2) comparable to that of compound **24**. The S(35), S(10), and S(1) selectivity scores at a concentration of 1 µM were 0.01, 0.002, and 0.002. Importantly, **26** demonstrated potent selectivity for PERK over the other three highlyconserved ISR kinases (GCN2, HRI, and PKR; Supplemental Table S3). In biochemical assays, the IC_50_ of **26** against PERK was 4.7 nM, compared to IC_50_ values greater than 10 µM for each of the other ISR kinases. The primary off-target activity of **26** was associated with FLT3 kinase and its mutant isoforms. Radiometric kinase assays were used to validate the kinome panel and determine the relative IC_50_ value of **26** against wild-type FLT3 and a mutant isoform (D835Y). When evaluated individually using concentrations of **26** up to 10 μM, the relative IC_50_ against wild-type FLT3 was >1 µM, and >10 µM against the mutant variant FLT3 (D385Y), indicating weak to no activity against FLT3wt or the mutant form (Supplemental Table S3).

**Figure 5:**
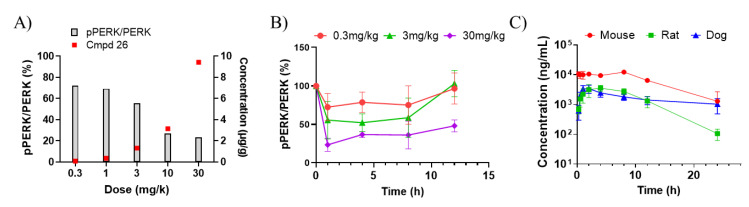
In vivo PK/PD analysis of **26**. (**A**) Inhibition of pPERK relative to total PERK protein (pPERK/PERK) in pancreas following oral administration of **26**. Abundance of **26** quantified by LC-MS/MS from matching pancreas samples at Cmax (1 h post administration). (**B**) Inhibition of pPERK/PERK in pancreas following oral administration of **26** at 0.3, 3, or 30 mg/kg. Pancreas samples taken at 1, 4, 8, 12 h following oral administration of **26**. Data represent mean ± SD of four mice. (**C**) Plasma PK analysis of **26** in mouse, rat and dog. **26** administered orally at 10 mg/kg, and sampled across a 24-h period; **26** quantified by LC-MS/MS.

To understand the effect of **26** in target organs, the PK/PD relationship of **26** was investigated in mouse pancreas. PK analysis of plasma and pancreas samples from mice treated with **26** revealed dose-proportionate exposure at doses ranging from 0.3 to 30 mg/kg following a single administration by oral gavage (Supplemental Figure S2). Pancreas tissue was sampled to evaluate the effect of **26** on PERK autophosphorylation in pancreas, following oral administration of **26** at 0.3, 1, 3, 10, and 30 mg/kg. At 1 h following administration, phosphoPERK (T980), relative to total PERK protein (pPERK/PERK) decreased in a dose-dependent manner, reaching approximately 80% inhibition at 30 mg/kg (Figure 5A). When PERK inhibition was evaluated across time in mouse pancreas, a dose-dependent inhibition was observed which was relatively stable across a 12 h period following administration of **26** (Figure 5B). This observation was consistent with the dose-proportionate exposure and low clearance rate of **26** observed in both mouse plasma and pancreas (Supplemental Figure S2). To confirm that the PK profile of **26** in other species was suitable to enable further development, plasma concentration of **26** was quantified in mouse, rat and dog following a single oral administration at 10 mg/kg. Consistent with observations in mice, rat and dog both shared similar clearance rates as mouse, albeit with different exposure levels following administration (Figure 5C).

**Figure 6:**
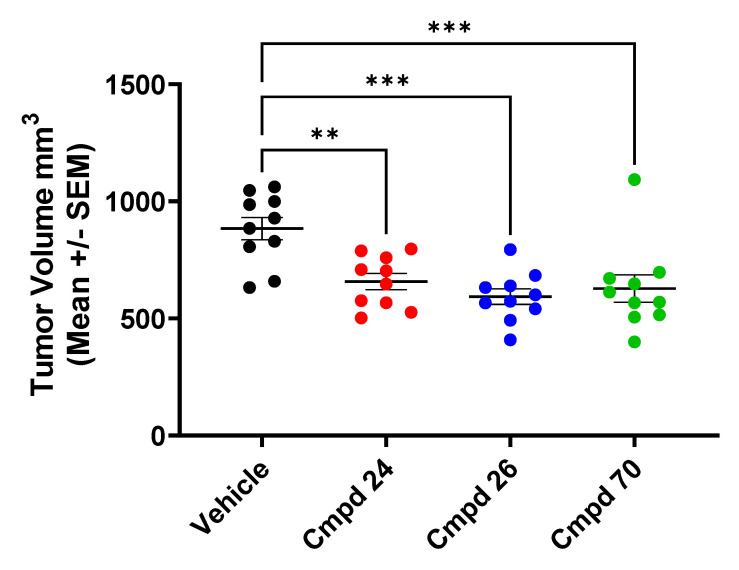
PERK inhibitors **24**, **26** and **70** inhibit tumor growth of 786-O RCC xenografts. Animals were treated at 30 mg/kg BID dosing for 29 days. Statistical analysis of group means by one-way ANOVA (** *p* < 0.01, *** *p* < 0.001).

As the focus of this program was to develop a novel, highly potent, selective and orally bioavailable PERK inhibitor to probe the role of PERK biology in animal models, compounds **24**, **26** and **70** were tested for in vivo efficacy in a clear cell renal cell carcinoma (ccRCC) xenograft model, 786-O (Figure 6). The 786-O model is driven by the Von Hippel-Lindau (VHL) mutation, which leads to PERK pathway activation and is therefore a suitable model to assess the biological activity of these PERK inhibitors [17]. The compounds were dosed at 30 mg/kg, twice per day (BID) for 29 days and were well tolerated as determined by body weight measurement (Supplemental Figure S3). Treatment with pyrrolopyrimidine PERK inhibitors resulted in significant tumor growth inhibition that was in line with our previously reported aminopyridine series of PERK inhibitors [7].

In summary, we have developed a novel class of potent pyrrolopyrimidine PERK inhibitors with excellent ADME and PK/PD properties. Robust kinase selectivity was achieved with substitution on the pyrrolopyrimidine hinge binder region driving kinase selectivity, with a methyl substitution at the 2-position of the ring resulting in exquisite selectivity for PERK over other kinases. Further optimization of the 2-methyl substituted analogs led to identification of lead molecule **26** that retained excellent potency and selectivity and had good in vitro metabolic stability across all species tested. When **26** was evaluated in vivo, high AUC_last_ values and a flat, sustained plasma exposure profile were observed in mouse plasma and pancreas tissue. A single administration of **26** resulted in stable PERK inhibition across a 12 h period in mouse pancreas. These characteristics led to the selection of **26** for further development, and evaluation in animal models is ongoing.

## Figures and Tables

**Table 1:** Reagents and conditions used in biochemical characterization of ISR kinases.

Protein	Carna Cat#	Assay Buffer
GCN2	05-153	GCN2 (1 nM); DTT (2 mM); ATP (150 µM); GFP-eIF2a (100 nM; Invitrogen Cat# PV4809)
HRI	05-154	HRI (0.12 nM); DTT (2 mM); ATP (30 µM); GFP-eIF2a (100 nM; Invitrogen Cat# PV4809)
PERK	05-155	PERK (0.09 nM); DTT (2 mM); ATP (8 µM); GFP-eIF2a (100 nM; Invitrogen Cat# PV4809)
PKR	05-156	PKR (0.11 nM); DTT (2 mM); ATP (6 µM); GFP-eIF2a (100 nM; Invitrogen Cat# PV4809)

**Table 2:** In vitro data of PERK inhibitors.

	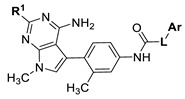			PERK	p-eIF2α
Cmpd	R^1^	L	Ar	IC_50_ (µM)	IC_50_ (µM)
**11**	H	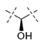	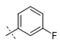	0.0022	0.0084
**12**	H	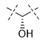	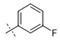	0.0078	0.057
**13**	H	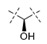	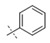	0.0018	0.023
**14**	H	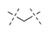	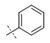	0.0052	0.041
**15**	H	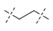	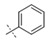	0.126	1.93
**16**	H	---	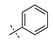	0.168	3.96
**17**	H	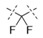	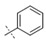	0.3	0.532
**18**	H	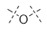	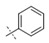	3.98	>10.0
**19**	H	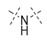	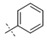	0.021	0.211
**20**	H	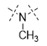	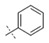	0.108	1.75
**21**	H	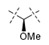	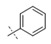	0.014	0.174
**22**	H	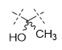	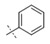	0.056	0.184
**23**	H	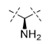	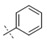	0.033	0.581
**24**	Me	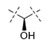	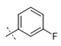	0.02	0.051
**25**	Me	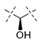	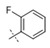	0.102	0.303
**26**	Me	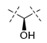	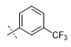	0.0028	0.015
**27**	Me	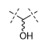	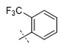	0.343	2.05
**28**	Me	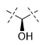	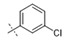	0.0018	0.016
**29**	Me	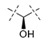	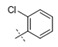	0.107	0.713
**30**	Me	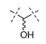	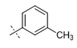	0.014	0.083
**31**	Me	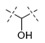	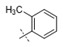	0.026	0.122
**32**	Me	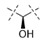	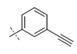	0.003	0.0078
**33**	Me	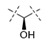	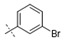	0.003	0.014
**34**	Me	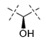	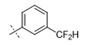	0.0059	0.021
**35**	Me	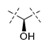	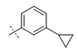	0.0059	0.017
**36**	Me	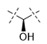	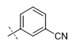	0.008	0.325
**37**	Me	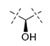	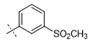	>1	>10
**38**	Me	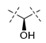	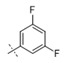	0.0019	0.014
**39**	Me	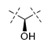	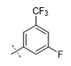	0.0026	0.011
**40**	Me	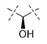	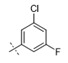	0.0029	0.0085
**41**	Me	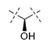	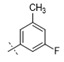	0.0014	0.01
**42**	Me	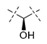	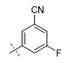	0.0042	0.032
**43**	Me	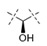	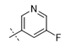	0.052	0.562
**44**	Me	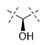	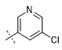	0.017	0.129
**45**	Me	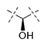	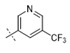	0.019	0.165

**Table 3:** In vitro data of PERK inhibitors with proximal and terminal ring modifications.

Cmpd No.	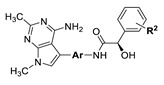		PERK IC_50_ (µM)	p-eIF2α IC_50_ (µM)
	Ar	R^2^		
**24**	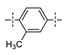	3-F	0.020	0.051
**46**	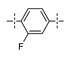	3-F	0.0022	0.041
**47**	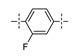	3-CF_3_	0.0051	0.011
**48**	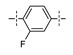	3-Cl	0.0010	0.010
**49**	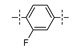	3-Me	0.0011	0.017
**50**	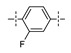	3-Et	0.0009	0.025
**51**	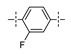	3-F, 5-CF_3_	0.0027	0.062
**52**	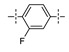	3-F, 5-Me	0.0011	0.011
**53**	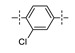	3-F	0.017	0.056
**54**	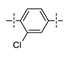	3-CF_3_	0.0027	0.021
**55**	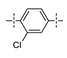	3-Cl	0.0016	0.017
**56**	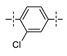	3-Me	0.0041	0.018
**57**	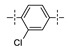	3-Et	0.059	0.014
**58**	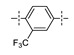	3-F	0.0085	0.204
**59**	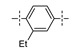	3-F	0.0040	0.041
**60**	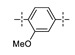	3-F	0.236	1.85
**61**	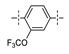	3-F	0.023	0.435
**62**	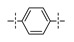	3-F	0.0020	0.126
**63**	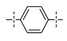	3-CF_3_	0.0028	0.013
**64**	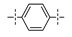	3-Br	0.0015	0.014
**65**	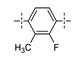	3-F	0.0019	0.030
**66**	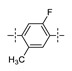	3-F	0.011	0.042
**67~**	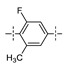	3-F	0.022	0.078
**68~**	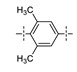	3-F	0.013	0.082
**69**	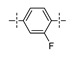	3-F	0.0010	0.026
**70**	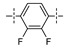	3-F	0.0015	0.024
**71~**	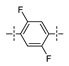	3-F	0.0010	0.034
**72**	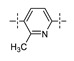	3-F	0.017	0.105
**73**	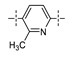	3-CF_3_	0.013	0.051
**74**	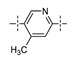	3-F	0.0083	0.146
**75**	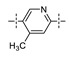	3-CF_3_	0.0068	0.095
**76**	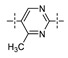	3-F	0.647	>10

~Racemic mixture of alcohols.

**Table 4:** Solubility, permeability, plasma protein binding and hepatocyte clearance properties of selected PERK inhibitors.

Cmpd No.	Kinetic Aqueous Solubility (µM)	Caco-2		Plasma Protein Binding				Hepatocyte Clearance (t_1/2_ min)			
		P_app_ (A→B) (10^−6^ cm/s)	Efflux Ratio	Human	Rat	Mouse	Dog	Human	Rat	Mouse	Dog
**26**	19	21	2.2	99.8 ± 0.0	99.8 ± 0.0	99.9 ± 0.0	99.5 ± 0.0	194	166	118	130
**28**	35	23	1.9	99.8 ± 0.1	99.7 ± 0.0	>99.9	98.3 ± 0.3	778	27	62	355
**65**	58	26	1.6	99.6 ± 0.1	98.9 ± 0.0	99.3 ± 0.2	98.7 ± 0.1	390	22	64	567
**24**	76	9	5.2	99.2 ± 0.2	99.1 ± 0.2	99.4 ± 0.0	97.0 ± 0.4	733	23	31	>1000
**41**	24	20	2.5	99.6 ± 0.2	99.5 ± 0.2	99.9 ± 0.0	98.8 ± 0.3	68	16	24	232
**39**	10	16	2.0	99.9 ± 0.0	99.9 ± 0.0	>99.9	>99.9	277	252	320	223
**38**	19	19	1.9	99.0 ± 0.1	99.1 ± 0.3	99.2 ± 0.3	98.4 ± 0.1	897	121	805	460
**48**	14	24	1.6	99.9 ± 0.0	99.6 ± 0.0	99.8 ± 0.0	99.6 ± 0.1	556	25	53	215
**66**	61	24	1.5	99.3 ± 0.2	98.6 ± 0.1	98.8 ± 0.2	98.2 ± 0.2	358	13	167	39
**70**	66	29	1.7	99.4 ± 0.0	99.3 ± 0.1	99.7 ± 0.1	99.4 ± 0.1	360	15	45	268

**Table 5:** In vivo pharmacokinetics (PK) properties of selected PERK inhibitors in mice. Compounds administered at 10 mg/kg by oral gavage (PO).

Compound	C_max_ (ng/mL)	AUC_0–last_ (h·ng/mL)	t_1/2_ (h)	F (%)
**26**	17,200	126,019	3.5	82
**28**	14,067	65,764	2.4	84
**65**	8150	40,105	2.2	129
**24**	13,100	37,495	2.1	77
**41**	12,467	26,010	2.7	40
**39**	14,300	86,444	2.9	78
**38**	8597	28,932	2.0	94
**48**	3017	10,889	2.1	70
**66**	9610	39,835	2.0	126
**70**	9723	32,258	2.0	117

## Data Availability

Coordinates and structural factors have been deposited in the PDB under codes 8EQ9 (Compound **11**), 8EQD (Compound **24**), and 8EQE (Compound **26**).

## Supplementary Materials

The following supporting information can be downloaded at: https://www.mdpi.com/article/10.3390/pharmaceutics14102233/s1, Supplemental Table S1: Crystallography data collection and refinement statistics; Supplemental Table S2:scanMAXSM Kinome Assay Results; Supplemental Table S3: Potency of Cmpd **26** against four ISR kinases and two FLT3 isoforms; Supplemental Figure S1: Compound **26** is selective against cell lines driven by FLT3-ITD; Supplemental Figure S2: In vivo PK in plasma and pancreas; Supplemental Figure S3: Compound **26** slows growth of 786-O RCC tumor xenografts; Supplemental Scheme S1: Synthesis of (*R*)-*N*-(4-(4-amino-7-methyl-7*H*-pyrrolo[2,3-*d*]pyrimidin-5-yl)-3-methylphenyl)-2-(3-fluorophenyl)-2-hydroxy acetamide (**11**) and (*S*)-*N*-(4-(4-amino-7-methyl-7*H*-pyrrolo[2,3-*d*]pyrimidin-5-yl)-3-methylphenyl) -2-(3-fluorophenyl) -2-hydroxyacetamide (**12**); Supplemental Scheme S2: Synthesis of (*S*)-*N*-(4-(4-amino-2,7-dimethyl-7*H*-pyrrolo[2,3-*d*]pyrimidin-5-yl)-2,3-difluoro phenyl)-2-(3-fluorophenyl)-2-hydroxyacetamide (**96**) and (*R*)-*N*-(4-(4-amino-2,7-dimethyl-7*H*-pyrrolo[2,3-*d*]pyrimidin-5-yl)-2,3-difluorophenyl)-2-(3-fluoro phenyl)-2-hydroxyacetamide (**70**); Supplemental Scheme S3: Synthesis of (*S*)-*N*-(4-(4-amino-2,7-dimethyl-7*H*-pyrrolo[2,3-*d*]pyrimidin-5-yl)-3-fluorophenyl)-2-hydroxy-2-(3-(trifluoromethyl)phenyl)acetamide (**106**) and (*R*)-*N*-(4-(4-amino-2,7-dimethyl-7*H*-pyrrolo[2,3-*d*]pyrimidin-5-yl)-3-fluorophenyl)-2-hydroxy-2-(3-(trifluoromethyl) phenyl)acetamide (**47**). References [7,17,20,21,22,23,24,25,27] are cited in the supplementary materials.
