# Supplementary material for: Optimization of a Novel Mandelamide-Derived Pyrrolopyrimidine Series of PERK Inhibitors
Source: Pharmaceutics. 2022 Oct 19;14(10):2233. doi: 10.3390/pharmaceutics14102233 (PMC9611727; doi:10.3390/pharmaceutics14102233)
Supplement: Supplementary file 1 [file pharmaceutics-14-02233-s001.zip › pharmaceutics-1924436-supplementary.pdf]

Supporting Information

Article

Optimization of a Novel Mandelamide-Derived Pyrrolopyrimidine Series of PERK Inhibitors

Michael E. Stokes [1,*,†], Matthew D. Surman [2,*,†], Veronica Calvo [1], David Surguladze [1], An-Hu Li [1], Jennifer Gasparek [3], Matthew Betzenhauser [3], Guangyu Zhu [3], Hongwen Du [4], Alan C. Rigby [1] and Mark J. Mulvihill [1]

[1] HiberCell Inc., 619 West 54th Street, New York, NY 10019, USA
[2] Curia, 26 Corporate Circle, Albany, NY 12203, USA
[3] Curia, 1001 Main Street, Buffalo, NY 14203, USA
[4] Pharmaron Beijing Co., Ltd., No. 6, TaiHe Road, BDA, Beijing 100176, China
* Correspondence: mstokes@hibercell.com (M.E.S.); matthew.surman@curiaglobal.com (M.D.S.)
† These authors contributed equally to this work.

Contents

1. Chemistry----------2
2. Crystallography---------- 42
3. Supplemental Data----------43
4. Materials and Methods----------49
5. References ----------56

1. Chemistry

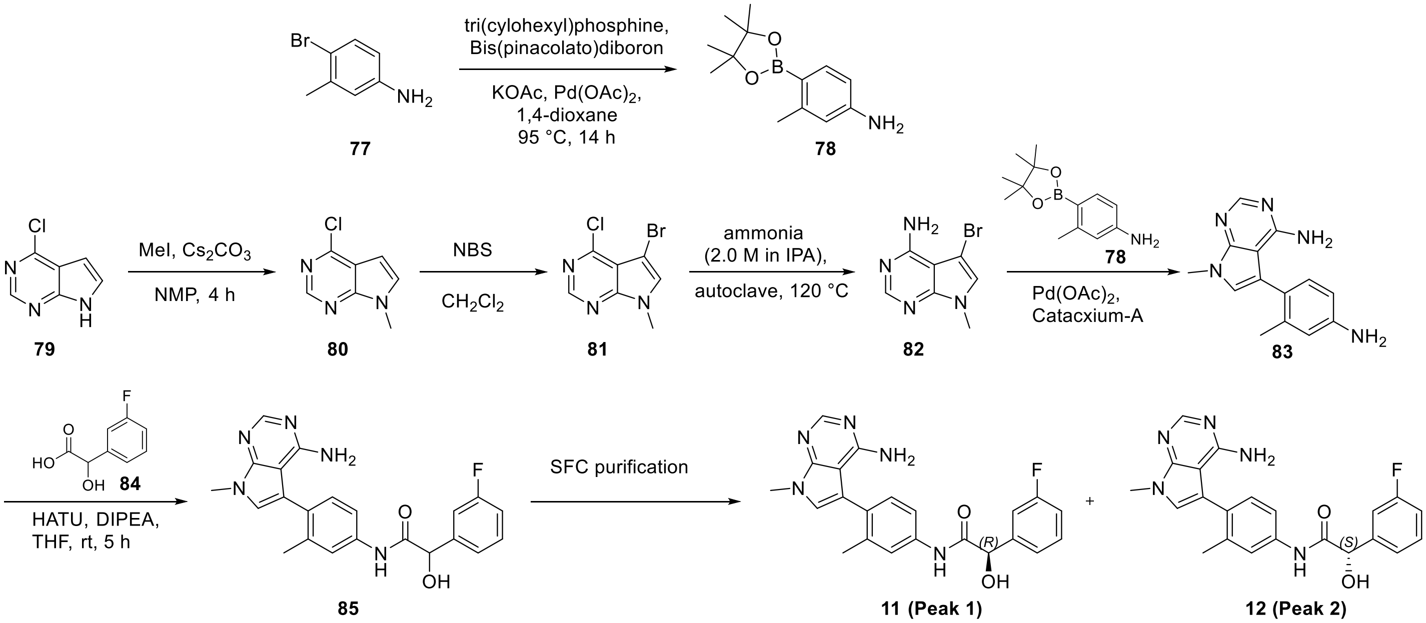

Scheme S1. Synthesis of (*R*)-*N*-(4-(4-amino-7-methyl-7*H*-pyrrolo[2,3-*d*]pyrimidin-5-yl)-3-methylphenyl)-2-(3-fluorophenyl)-2-hydroxy acetamide (11) and (***S***)**-*N*-(4-(4-amino-7-methyl-7*H*-pyrrolo[2,3-*d*]pyrimidin-5-yl)-3-methylphenyl) -2-(3-fluorophenyl) -2-hydroxyacetamide** (**12**).

3-Methyl-4-(4,4,5,5-tetramethyl-1,3,2-dioxaborolan-2-yl)aniline (**78**)

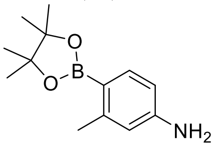

78

To a stirred solution of tricyclohexylphosphine (7.18 g, 25.7 mmol) in 1,4-dioxane (1.2 L) under argon atmosphere were added sequentially bis(pinacolato)diboron (89.62 g, 352.9 mmol), potassium acetate (62.98 g, 641.7 mmol) and 4-bromo-3-methylaniline (**77,** 59.69 g, 320.8 mmol). The reaction mixture was purged with argon gas for 10 min, treated with palladium (II) acetate (5.77 g, 25.7 mmol) and again purged with argon gas for an additional 10 min. The reaction mixture was heated with stirring at 95 °C under an argon atmosphere for overnight. After this time, the reaction mixture was allowed to cool to room temperature, passed through a bed of diatomaceous earth and washed with methyl *tert*-butyl ether (4 × 250 mL). The filtrate was washed with water (2 × 500 mL) and brine solution (2 × 250 mL). The organic layer was separated, dried over anhydrous sodium sulfate and concentrated under reduced pressure. The crude material was purified by column chromatography (silica gel (100-200 mesh size), 10% ethyl acetate/hexanes as an eluent) to afford 3-methyl-4-(4,4,5,5-tetramethyl-1,3,2-dioxaborolan-2-yl)aniline (**78**, 44.80 g, 60%) as a pale brown solid. ^{1}H NMR (400 MHz, DMSO-d_6) δ 7.32–7.30 (d, *J* = 8.0 Hz, 1H), 6.31 (s, 1H), 6.29 (s, 1H), 5.34 (s, 2H), 2.29 (s, 3H), 1.23 (s, 12H). ESI (*m*/*z*): 234 [$C_{13}H_{20}BNO_2$ + H]$^+$.

4-Chloro-7-methyl-7*H*-pyrrolo[2,3-*d*]pyrimidine (80)

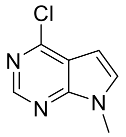

80

To a stirred solution of 4-chloro-7*H*-pyrrolo[2,3-*d*]pyrimidine (**79**, 5.00 g, 32.5 mmol) in *N*-methyl-2-pyrrolidone (30 mL) was added cesium carbonate (21.2 g, 65.1 mmol) at 15 °C. After 15 min, methyl iodide (2.32 g, 1.0 mL, 16.4 mmol) was added dropwise at 15 °C, and the resulting mixture was stirred under argon atmosphere at ambient temperature for 4 h. After this time, the reaction mixture was poured into ice cold water (60 mL) and stirred for 30 min. The solid was precipitated out, isolated by filtration, washed with water (30 mL) and dried under vacuum to afford 4-chloro-7-methyl-7*H*-pyrrolo[2,3-*d*]pyrimidine (**80**, 4.4 g, 80%) as an off white solid. ESI (*m*/*z*): 168 [$C_7H_6ClN_3$ + H]$^+$.

5-Bromo-4-chloro-7-methyl-7*H*-pyrrolo[2,3-*d*]pyrimidine (81)

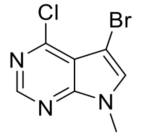

81

To a stirred solution of 4-chloro-7-methyl-7*H*-pyrrolo[2,3-*d*]pyrimidine (**80**, 4.41 g, 26.3 mmol) in methylene chloride (40 mL) was added *N*-bromosuccinimide (5.14 g, 28.9 mmol) portion-wise at 5 °C. The resulting mixture was warmed to ambient temperature and stirred for 3 h. After this time, the reaction mixture was filtered, solid was washed with water (40 mL) and dried to afford 5-bromo-4-chloro-7-methyl-7*H*-pyrrolo[2,3-*d*]pyrimidine (**81**, 5.1 g, 78%) as an off white solid. ESI (*m*/*z*): 246, 249 [$C_7H_5BrClN_3$ + H]$^+$.

5-Bromo-7-methyl-7*H*-pyrrolo[2,3-*d*]pyrimidin-4-amine (82)

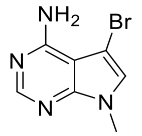

82

In a 100 mL autoclave, 5-bromo-4-chloro-7-methyl-7*H*-pyrrolo[2,3-d]pyrimidine (**81**, 3.0 g, 12.19 mmol) in 25% aqueous ammonia (25 mL) was heated with stirring at 120 °C for 16 h. After this time, the reaction mixture was allowed to cool to room temperature. The resulting solid was filtrated, washed with water (25 mL) followed by methanol (5 mL) and dried to afford 5-bromo-7-methyl-7*H*-pyrrolo[2,3-*d*]pyrimidin-4-amine (**82**, 2.1 g, 77%) as an off white solid. ESI (*m*/*z*) 227, 229 [$C_7H_7BrN_4$ + H]$^+$.

5-(4-Amino-2-methylphenyl)-7-methyl-7*H*-pyrrolo[2,3-*d*]pyrimidin-4-amine (83)

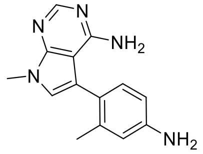

83

To a mixture of 2-methyl tetrahydrofuran (15 mL) and water (5 mL), 5-bromo-7-methyl-7*H*-pyrrolo[2,3-*d*]pyrimidin-4-amine (**82**, 2.00 g, 8.81 mmol), 3-methyl-4-(4,4,5,5-tetramethyl-1,3,2-dioxaborolan-2-yl)aniline (**78,** 2.66 g, 11.5 mmol) were added. The resulting reaction mixture was purged with argon for 5 min. followed by addition of sodium bicarbonate (1.48 g, 17.6 mmol.), Catacxium-A (0.19 g, 0.52 mmol) and palladium(II) acetate (0.058 g, 0.26 mmol). After addition was complete, the resulting reaction mixture was purged again with argon for 10 min. and then heated at 100 °C for 16 h. After this time, the mixture was allowed to cool to room temperature, passed through a bed of diatomaceous earth and washed with ethyl acetate (2 × 50 mL). The filtrate was washed with water (20 mL) and brine solution (20 mL). The organic layer was dried over anhydrous sodium sulfate and concentrated under reduced pressure. Crude product was purified by reverse phase column chromatography (40% ACN: Water) to afford 5-(4-amino-2-methylphenyl)-7-methyl-7*H*-pyrrolo[2,3-*d*]pyrimidin-4-amine (**83**, 0.8 g, 36%) as an off white solid. ESI (*m*/*z*): 254 [$C_{14}H_{15}N_5$ + H]$^+$.

***N*-(4-(4-Amino-7-methyl-7*H*-pyrrolo[2,3-*d*]pyrimidin-5-yl)-3-methylphenyl)-2-(3-fluorophenyl)-2-hydroxyacetamide (85)**

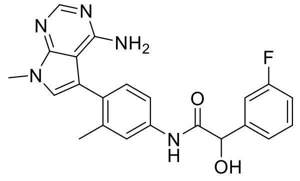

85

To a solution of 5-(4-amino-2-methylphenyl)-7-methyl-7*H*-pyrrolo[2,3-*d*]pyrimidin-4-amine (**83**, 800 mg, 3.16 mmol), 2-(3-fluorophenyl)-2-hydroxyacetic acid (**84**, 591 mg, 3.47 mmol) in tetrahydrofuran (5 mL) were added *N*,*N*-diisopropylethylamine (0.70 mL, 3.8 mmol) followed by 1-[bis(dimethylamino)methylene]-1*H*-1,2,3-triazolo[4,5-*b*]pyridinium 3-oxide hexafluorophosphate (HATU) (1.44 g, 3.79 mmol) at room temperature, and the reaction mixture was stirred for 12 h. After this time, the reaction mixture was diluted with methylene chloride (20 mL) and washed with water (4 × 10 mL) followed by brine solution (10 mL). The organic layer was separated, dried over anhydrous sodium sulfate, and concentrated under reduced pressure. The crude product was purified by combi flash column chromatography (using 2% methanol/methylene chloride as an eluent) to afford *N*-(4-(4-amino-7-methyl-7*H*-pyrrolo[2,3-*d*]pyrimidin-5-yl)-3-methylphenyl)-2-(3-fluorophenyl)-2-hydroxyacetamide (**85**, 900 mg, 70%) as a mixture of enantiomers as an off white solid:
^{1}H NMR (400 MHz, DMSO-d_6): δ 10.00 (s, 1H), 8.17 (s, 1H), 7.69 (s, 1H), 7.62 (d, *J* = 8.80 Hz, 1H), 7.42–7.34 (m, 3H), 7.19–7.14 (m, 3H), 5.17 (d, *J* = 4.40 Hz, 2H), 5.75 (s, 3H), 2.16 (s, 3H); ESI (*m*/*z*): 406 [$C_{22}H_{20}FN_5O_2$ + H]$^+$.

(*R*)-*N*-(4-(4-Amino-7-methyl-7*H*-pyrrolo[2,3-d]pyrimidin-5-yl)-3-methylphenyl)-2-(3-fluorophenyl)-2-hydroxyacetamide (11) and **(*S*)-*N*-(4-(4-amino-7-methyl-7*H*-pyrrolo[2,3-*d*]pyrimidine-5-yl)-3-methyl phenyl)-2-(3-fluorophenyl)-2-hydroxyacetamide** (**12**)

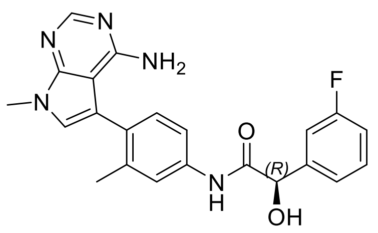

11 (Peak 1)

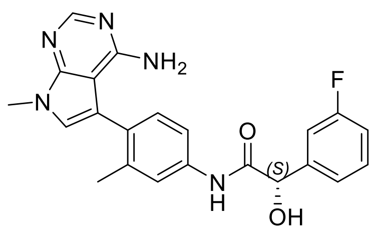

12 (Peak 2)

The mixture of enantiomers was purified by chiral supercritical fluid chromatography (SFC) (Chiralcel OX-H column, 30% methanol in CO_2, 40 °C temperature).

11/Peak 1) (52 mg) as an off white solid; ^{1}H NMR (400 MHz, DMSO-*d*$_6$) 9.98 (s, 1H), 8.13 (s, 1H), 7.68 (d, *J* = 1.60 Hz, 1H), 7.61 (dd, *J* = 1.60, 8.20 Hz, 1H), 7.42–7.34 (m, 3H), 7.17–7.14 (m, 3H), 6.59 (d, *J* = 4.80 Hz, 1H), 5.58 (br s, 2H), 5.16 (d, *J* = 4.80 Hz, 1H), 3.73 (s, 3H), 2.15 (s, 3H); HPLC (Method C) 99.3% (AUC), t_R = 6.46 min; Chiral SFC (Chiralcel OX-H, Method B) 99.2% (AUC), t_R = 5.89 min; ESI (*m/z*): 406 [$C_{22}H_{20}FN_5O_2$ + H]$^+$.

12/Peak 2) (35 mg) as a light-yellow solid; ^{1}H NMR (400 MHz, DMSO-*d*$_6$) δ 9.98 (s, 1H), 8.12 (s, 1H), 7.68 (s, 1H), 7.61 (d, *J* = 8.00 Hz, 1H), 7.42–7.34 (m, 3H), 7.17–7.13 (m, 3H), 6.59 (d, *J* = 4.80 Hz, 1H), 5.58 (br s, 2H), 5.16 (d, *J* = 4.80 Hz, 1H), 3.73 (s, 3H), 2.15 (s, 3H); HPLC (Method A) 99.3% (AUC), t_R = 6.44 min; Chiral SFC (Chiralcel OX-H, Method A) 99.3% (AUC), t_R = 7.63 min. ESI (m/z): 406 [$C_{22}H_{20}FN_5O_2$ + H]$^+$.

The following compounds were prepared by following the same procedure for synthesizing **11** and **12**:

(*R*)-*N*-(4-(4-Amino-7-methyl-7*H*-pyrrolo[2,3-*d*]pyrimidin-5-yl)-3-methylphenyl)-2-hydroxy-2-phenylacetamide (13)

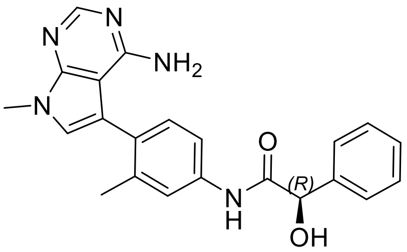

13

^{1}H NMR (400 MHz, DMSO-*d*$_6$) δ 9.95 (s, 1H), 8.12 (s, 1H), 7.69 (d, *J* = 1.60 Hz, 1H), 7.62 (dd, *J* = 2.40 , 8.20 Hz, 1H), 7.53 (d, *J* = 7.60 Hz, 2H), 7.38–7.30 (m, 3H), 7.15 (d, *J* = 8.40 Hz, 1H), 7.13 (s, 1H), 6.45 (d, *J* = 4.80 Hz, 1H), 5.76 (s, 2H), 5.12 (d, *J* = 4.80 Hz, 1H), 3.73 (s, 3H), 2.15 (s, 3H); ESI (*m/z*): 388 [$C_{22}H_{21}N_5O_2$ + H]$^+$; HPLC (Method C) > 99% (AUC), t_R = 6.37 min; Chiral SFC (Chiralcel OX-H, Method B) 98.5% (AUC), t_R = 5.93 min.

***N*-(4-(4-Amino-7-methyl-7*H*-pyrrolo[2,3-*d*]pyrimidin-5-yl)-3-methylphenyl)-2-phenylacetamide (14)**

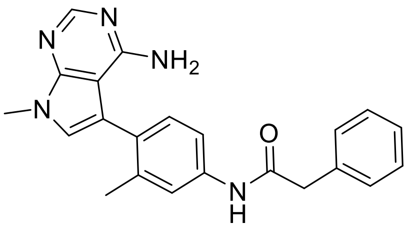

14

^{1}H NMR (400 MHz, DMSO-*d*$_6$) δ 10.23 (s, 1H), 8.13 (s, 1H), 7.59 (d, *J* = 1.60 Hz, 1H), 7.52 (dd, *J* = 1.60 , 8.20 Hz, 1H), 7.37–7.32 (m, 4H), 7.28–7.23 (m, 1H), 7.16 (d, *J* = 8.00 Hz, 1H), 7.13 (s, 1H), 5.62 (brs, 2H), 3.73 (s, 3H), 3.65 (s, 2H), 2.16 (s, 3H); ESI (*m/z*): 372 [$C_{22}H_{21}N_5O$ + H]$^+$; HPLC (Method C) >99.4% (AUC), t_R = 6.51 min.

***N*-(4-(4-Amino-7-methyl-7*H*-pyrrolo[2,3-*d*]pyrimidin-5-yl)-3-methylphenyl)-3-phenylpropanamide (15)**

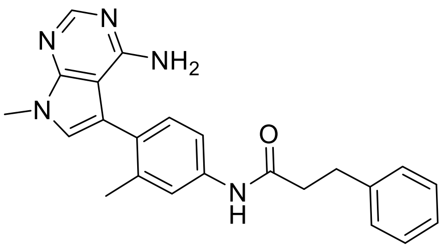

15

^{1}H NMR (400 MHz, DMSO-d_6) δ 9.99 (brs, 1H), 8.12 (s, 1H), 7.56 (s, 1H), 7.48 (dd, J = 11.20 , 11.60 Hz, 1H), 7.35–7.21 (m, 4H), 7.11–7.18 (m, 3H), 5.73 (brs, 2H), 3.72 (s, 3H), 2.94–2.91 (m, 2H), 2.64–2.60 (m, 2H), 2.20 (s, 3H); ESI (*m/z*): 386 [$C_{23}H_{23}N_5O$ + H]$^+$; HPLC (Method B) 95.7% (AUC), t_R = 8.40 min.

***N*-(4-(4-Amino-7-methyl-7*H*-pyrrolo[2,3-*d*]pyrimidin-5-yl)-3-methylphenyl)benzamide (16)**

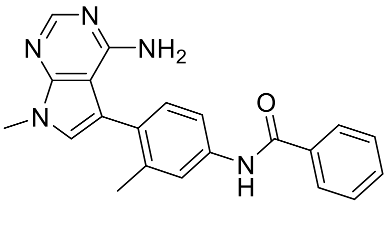

16

^{1}H NMR (400 MHz, DMSO-d_6) δ 10.3 (s, 1H), 8.14 (s, 1H), 7.97 (d, J = 7.20 Hz, 2H), 7.79 (s, 1H), 7.72 (dd, J = 1.60 , 8.20 Hz, 1H), 7.61–7.53 (m, 3H), 7.23 (d, J = 8.00 Hz, 1H), 7.17 (s, 1H), 5.76 (brs, 2H), 3.75 (s, 3H), 2.21 (s, 3H); ESI (*m/z*): 358 [$C_{21}H_{19}N_5O$ + H]$^+$; HPLC (Method B) >99 % (AUC), t_R = 6.40 min.

***N*-(4-(4-Amino-7-methyl-7*H*-pyrrolo[2,3-*d*]pyrimidin-5-yl)-3-methylphenyl)-2,2-difluoro-2-phenylacetamide (17)**

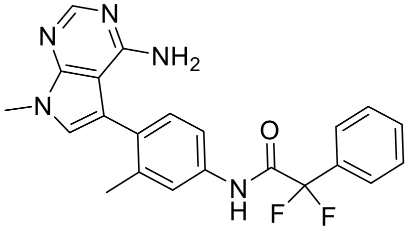

17

^{1}H NMR (400 MHz, DMSO-d_6) δ 10.80 (s, 1H), 8.13 (s, 1H), 7.71–7.69 (m, 3H), 7.65–7.57 (m, 4H), 7.22 (d, J = 8.0 Hz, 1H), 7.15 (s, 1H), 5.79–5.53 (m, 2H), 3.73 (s, 3H), 2.18 (s, 3H). ESI (*m/z*): 408 [$C_{22}H_{19}F_2N_5O$ + H] $^+$; HPLC (Method B) 97.6% (AUC), t_R = 7.46 min.

(*R*)-*N*-(4-(4-Amino-7-methyl-7*H*-pyrrolo[2,3-*d*]pyrimidin-5-yl)-3-methylphenyl)-2-methoxy-2-phenylacetamide (21)

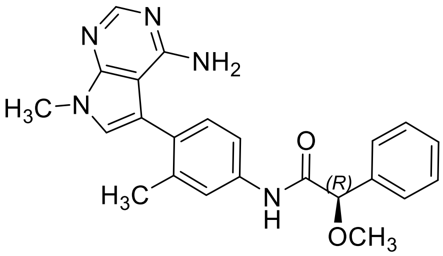

21

^{1}H NMR (400 MHz, DMSO-d$_6$) δ 10.08 (s, 1H), 8.13 (s, 1H), 7.67 (s, 1H), 7.60 (dd, J = 4.00 , 8.00 Hz, 1H), 7.50 (d, J = 8.00 Hz, 2H), 7.41–7.32 (m, 3H), 7.17 (s, 1H), 7.14 (s, 1H), 5.75 (s, 2H), 4.85 (s, 1H), 3.73 (s, 3H), 3.38 (s, 3H), 2.15 (s, 3H); ESI (m/z): 402 [$C_{23}H_{23}N_5O_2$ + H]$^+$; HPLC (Method C) >99% (AUC), t_R = 6.57 min.

N-(4-(4-Amino-7-methyl-7H-pyrrolo[2,3-d]pyrimidin-5-yl)-3-methylphenyl)-2-hydroxy-2-phenylpropanamide (22)

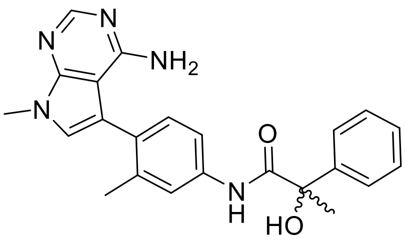

22

^{1}H NMR (400 MHz, DMSO-d_6) δ 9.73 (s, 1H), 8.12 (s, 1H), 7.73 (s, 1H), 7.64–7.61 (m, 3H), 7.36 (m, 2H), 7.29–7.25 (m, 1H), 7.15–7.12 (m, 2H), 6.49 (s, 1H), 5.68 (brs, 2H), 3.73 (s, 3H), 2.14 (s, 3H), 1.74 (s, 3H); ESI (*m*/*z*): 402 [$C_{23}H_{23}N_5O_2$ + H]$^+$; HPLC (Method B) >99% (AUC), t_R = 7.22 min.

(*R*)-2-Amino-N-(4-(4-Amino-7-methyl-7*H*-pyrrolo[2,3-*d*]pyrimidin-5-yl)-3-methylphenyl)-2-phenylacetamide (23)

The Boc group was then removed using trifluoroacetic acid in methylene chloride to give **23**.

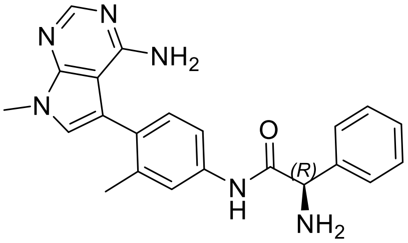

23

^{1}H NMR (400 MHz, DMSO-d_6) δ 10.07 (brs, 1H), 8.11 (s, 1H), 7.59–7.54 (m, 4H), 7.36–7.32 (m, 2H), 7.28–7.24 (m, 1H), 7.15 (d, *J* = 8.00 Hz, 1H), 7.12 (s, 1H), 5.69 (brs, 2H), 4.55 (s, 1H), 3.72 (s, 3H), 2.15 (s, 3H); ESI (*m*/*z*): 387 [$C_{22}H_{22}N_6O$ + H]$^+$; HPLC (Method C) >99% (AUC), t_R = 5.99 min.

(*R*)-*N*-(4-(4-Amino-2,7-dimethyl-7*H*-pyrrolo[2,3-*d*]pyrimidin-5-yl)-3-methylphenyl)-2-(3-fluorophenyl)-2-hydroxyacetamide (24)

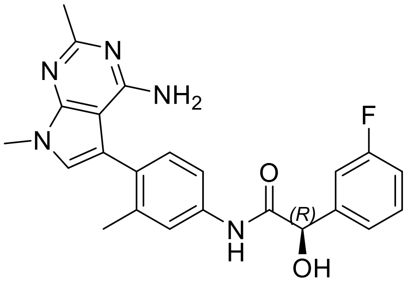

24

^{1}H NMR (400 MHz, DMSO-d_6) δ 9.95 (s, 1H), 7.66 (s, 1H), 7.59 (d, *J* = 8.4 Hz, 1H), 7.41–7.33 (m, 3H), 7.14 (d, *J* = 8.4 Hz, 2H), 7.01 (s, 1H), 6.57 (d, *J* = 4.8 Hz, 1H), 5.54 (brs, 2H), 5.15 (d, *J* = 4.8 Hz, 1H), 3.68 (s, 3H), 2.39 (s, 3H), 2.14 (s, 3H); ESI (m/z): 420 [$C_{23}H_{22}FN_5O_2$ + H]$^+$; HPLC (Method C) 94.6% (AUC), t_R = 6.52 min; Chiral SFC (Chiralcel OX-H, Method B) >99% (AUC), t_R = 5.26 min.

(*R*)-*N*-(4-(4-Amino-2,7-dimethyl-7*H*-pyrrolo[2,3-*d*]pyrimidin-5-yl)-3-methylphenyl)-2-(2-fluorophenyl)-2-hydroxyacetamide (25)

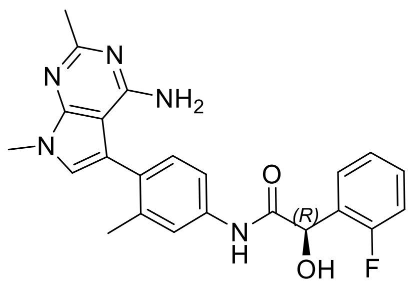

25

^{1}H NMR (400 MHz, DMSO-d_6) δ 9.98 (s, 1H), 7.71 (d, *J* = 4.00 Hz, 1H), 7.63–7.61 (m, 1H), 7.56–7.52 (m, 1H), 7.39–7.34 (m, 1H), 7.24–7.14 (m, 3H), 7.03 (s, 1H), 6.62 (d, *J* = 4.00 Hz, 1H), 5.71 (brs, 2H), 5.36 (d, *J* = 8.00 Hz, 1H), 3.69 (s, 3H), 2.40 (s, 3H), 2.15 (s, 3H); ESI (*m*/*z*): 420 $[C_{23}H_{22}FN_5O_2 + H]^+$; HPLC (Method B) 97.3% (AUC), t_R = 7.79 min; Chiral SFC (Chiralpak IA, Method A) >99% (AUC), t_R = 4.82 min.

***N*-(4-(4-Amino-2,7-dimethyl-7*H*-pyrrolo[2,3-*d*]pyrimidin-5-yl)-3-methylphenyl)-2-hydroxy-2-(2-(trifluoromethyl) phenyl) acetamide (27)**

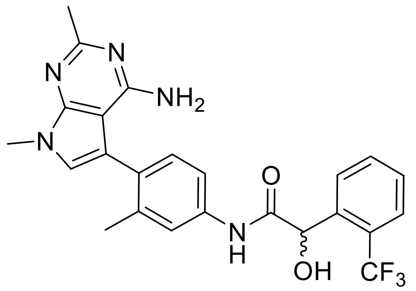

27

^{1}H NMR (400 MHz, DMSO-d_6) δ 10.13 (s, 1H), 7.78–7.70 (m, 4H), 7.62 (dd, *J* = 1.60, 8.20 Hz, 1H), 7.54 (t, *J* = 7.60 Hz, 1H), 7.16 (d, *J* = 8.40 Hz, 1H), 7.04 (s, 1H), 6.86 (brs, 1H), 5.67 (brs, 2H), 5.44 (s, 1H), 3.69 (s, 3H), 2.40 (s, 3H), 2.16 (s, 3H); ESI (*m*/*z*): 470 $[C_{24}H_{22}F_3N_5O_2 + H]^+$; HPLC (Method B) 98.4% (AUC), t_R = 8.35 min.

(*R*)-*N*-(4-(4-Amino-2,7-dimethyl-7*H*-pyrrolo[2,3-*d*]pyrimidin-5-yl)-3-methylphenyl)-2-(2-chlorophenyl)-2-hydroxyacetamide (29)

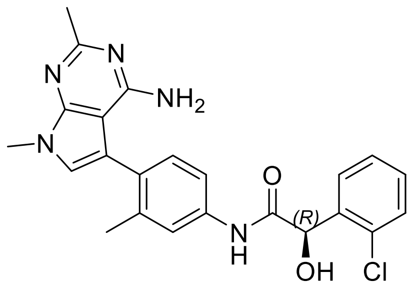

29

^{1}H-NMR (400 MHz, DMSO-d_6) *δ* 10.03 (s, 1H), 7.71 (s, 1H), 7.63–7.58 (m, 2H), 7.45 (dd, *J* =1.60, 7.60 Hz, 1H), 7.39–7.34 (m, 2H), 7.16 (d, *J* = 8.00 Hz, 1H), 7.04 (s, 1H), 6.65 (d, *J* = 5.60 Hz, 1H), 5.72 (brs, 2H), 5.49 (d, *J* = 5.20 Hz, 1H), 3.70 (s, 3H), 2.41 (s, 3H), 2.16 (s, 3H); ESI (m/z): 436 $[C_{23}H_{22}ClN_5O_2+H]^+$; HPLC (Method B) 98.5% (AUC), t_R = 8.16 min; Chiral SFC (Chiralcel OX-H, Method B) >99% (AUC), t_R = 2.62 min.

(*R*)-*N*-(4-(4-Amino-2,7-dimethyl-7*H*-pyrrolo[2,3-*d*]pyrimidin-5-yl)-3-methylphenyl)-2-hydroxy-2-(m-tolyl)acetamide (30)

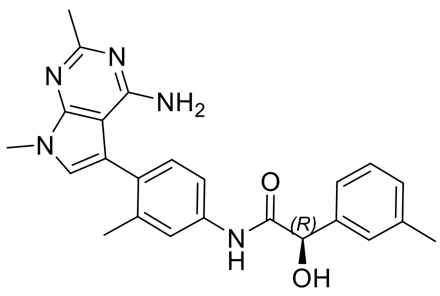

30

^{1}H NMR (400 MHz, DMSO-d_6) δ 9.89 (s, 1H), 7.68 (d, *J* = 4.00 Hz, 1H), 7.61–7.59 (m, 1H), 7.33–7.30 (m, 2H), 7.24 (t, *J* = 8.00 Hz, 1H), 7.14–7.09 (m, 2H), 7.02 (s, 1H), 6.39 (d, *J* = 4.00 Hz, 1H), 5.58 (brs, 2H), 5.06 (d, *J* = 4.00 Hz, 1H), 3.68 (s, 3H), 2.39 (s, 3H), 2.31 (s, 3H), 2.14 (s, 3H); ESI (m/z): 416 [$C_{24}H_{25}N_5O_2$ + H]$^+$; HPLC (Method B) >99% (AUC), t_R = 8.11 min; Chiral SFC (Chiralpak IA, Method A) >99% (AUC), t_R = 4.16 min.

***N*-(4-(4-Amino-2,7-dimethyl-7*H*-pyrrolo[2,3-*d*]pyrimidin-5-yl)-3-methylphenyl)-2-hydroxy-2-(o-tolyl)acetamide (31)**

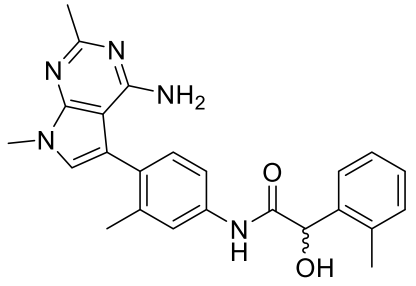

31

^{1}H NMR (400 MHz, DMSO-d_6) δ 9.90 (s, 1H), 7.72 (d, *J* = 1.60 Hz, 1H), 7.63 (dd, *J* = 1.60 , 8.20 Hz, 1H), 7.46–7.44 (m, 1H), 7.21–7.14 (m, 4H), 7.03 (s, 1H), 6.42 (d, *J* = 4.80 Hz, 1H), 5.66 (s, 2H), 5.31 (d, *J* = 3.20 Hz, 1H), 3.69 (s, 3H), 2.44 (s, 3H), 2.40 (s, 3H), 2.15 (s, 3H); ESI (*m*/*z*): 416 [$C_{24}H_{25}N_5O_2$ + H]$^+$; HPLC (Method B) 97.5% (AUC), t_R = 8.09 min.

(*R*)-*N*-(4-(4-Amino-2,7-dimethyl-7*H*-pyrrolo[2,3-*d*]pyrimidin-5-yl)-3-methylphenyl)-2-(3-ethynylphenyl)-2-hydroxy acetamide (32)

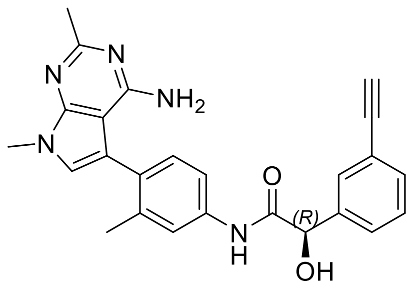

32

^{1}H NMR (400 MHz, DMSO-d_6) *δ* 9.94 (brs, 1H), 7.67–7.64 (m, 2H), 7.61–7.55 (m, 2H), 7.42–7.36 (m, 2H), 7.14 (d, *J* = 8.00 Hz, 1H), 7.01 (s, 1H), 6.57 (brs, 1H), 5.60 (brs, 2H), 5.13 (s, 1H), 4.20 (s, 1H), 3.68 (s, 3H), 2.39 (s, 3H), 2.14 (s, 3H); ESI (*m*/*z*): 426 [$C_{25}H_{23}N_5O_2$ + H]$^+$; HPLC (Method A) 97.1% (AUC), t_R = 3.19 min; Chiral SFC (Chiralpak IB, Method D) >99% (AUC), t_R = 4.82 min.

(*R*)-*N*-(4-(4-Amino-2,7-dimethyl-7*H*-pyrrolo[2,3-*d*]pyrimidin-5-yl)-3-methylphenyl)-2-(3-bromophenyl)-2-hydroxyacetamide (33)

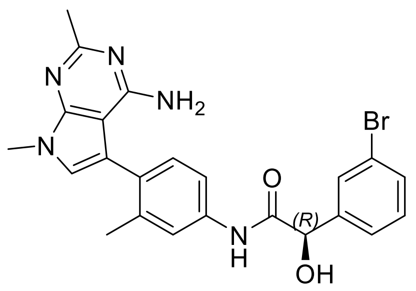

33

^{1}H NMR (400 MHz, DMSO-d_6) δ 9.96 (s, 1H), 7.73 (s, 1H), 7.67 (d, *J* = 2.00 Hz, 1H), 7.60–7.58 (m, 1H), 7.53–7.49 (m, 2H), 7.35–7.31 (m, 1H), 7.14 (d, *J* = 8.40 Hz, 1H), 7.02 (s, 1H), 6.58 (d, *J* = 8.00 Hz, 1H), 5.60 (brs, 2H), 5.15 (d, *J* = 4.00 Hz, 1H), 3.68 (s, 3H), 2.39 (s, 3H), 2.14 (s, 3H); ESI (*m*/*z*): 480 [$C_{23}H_{22}BrN_5O_2$ + H]$^+$; HPLC (Method A) >99% (AUC), t_R = 3.38 min; Chiral SFC (Chiralcel OX-H, Method B) 97.1% (AUC), t_R = 2.43 min.

(*R*)-*N*-(4-(4-Amino-2,7-dimethyl-7*H*-pyrrolo[2,3-*d*]pyrimidin-5-yl)-3-methylphenyl)-2-(5-fluoropyridin-3-yl)-2-hydroxyacetamide (43)

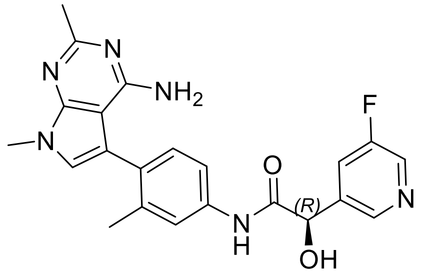

43

^{1}H NMR (400 MHz, DMSO-d_6) δ 10.06 (s, 1H), 8.61 (s, 1H), 8.54 (d, *J* = 2.80 Hz, 1H), 7.83–7.80 (m, 1H), 7.67 (d, *J* = 2.00 Hz, 1H), 7.61–7.59 (m, 1H), 7.16 (d, *J* = 8.00 Hz, 1H), 7.04 (s, 1H), 6.82 (d, *J* = 5.20 Hz, 1H), 5.63 (brs, 2H), 5.30 (d, *J* = 5.20 Hz, 1H), 3.69 (s, 3H), 2.40 (s, 3H), 2.15 (s, 3H); ESI (*m*/*z*): 421 [$C_{22}H_{21}FN_6O_2$ + H]$^+$; HPLC (Method A) 98.5% (AUC), t_R = 2.75 min; Chiral SFC (Chiralcel OX-H, Method B) > 99% (AUC), t_R = 1.35 min.

(*R*)-*N*-(4-(4-Amino-2,7-dimethyl-7*H*-pyrrolo[2,3-*d*]pyrimidin-5-yl)-3-methylphenyl)-2-(5-chloropyridin-3-yl)-2-hydroxyacetamide (44)

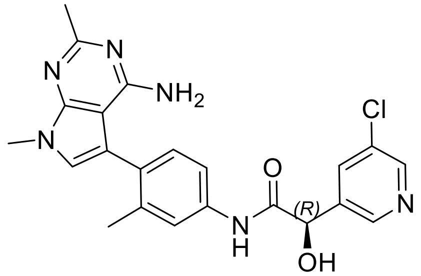

44

^{1}H NMR (400 MHz, DMSO-d_6) δ 10.04 (s, 1H), 8.68 (s, 1H), 8.59 (d, *J* = 2.00 Hz, 1H), 8.03–8.02 (m, 1H), 7.67 (d, *J* = 2.00 Hz, 1H), 7.61–7.58 (m, 1H), 7.15 (d, *J* = 8.00 Hz, 1H), 7.02 (s, 1H), 6.81 (d, *J* = 4.80 Hz, 1H), 5.60 (brs, 2H), 5.28 (d, *J* = 5.20 Hz, 1H), 3.69 (s, 3H), 2.40 (s, 3H), 2.15 (s, 3H); ESI (*m*/*z*): 437 [$C_{22}H_{21}ClN_6O_2$ + H]$^+$; HPLC (Method A) > 99% (AUC), t_R = 2.97 min; Chiral SFC (Chiralcel OX-H, Method E) 98.5% (AUC), t_R = 1.71 min.

(*R*)-*N*-(4-(4-Amino-2,7-dimethyl-7*H*-pyrrolo[2,3-*d*]pyrimidin-5-yl)-3-ethylphenyl)-2-(3-fluorophenyl)-2-hydroxyacetamide (59)

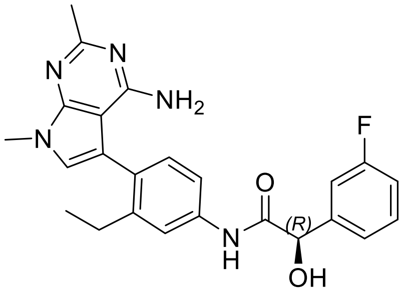

59

^{1}H NMR (400 MHz, DMSO-d_6) δ 9.98 (s, 1H), 7.70 (d, *J* = 2.00 Hz, 1H), 7.62 (dd, *J* = 2.00 , 8.40 Hz, 1H), 7.44–7.34 (m, 3H), 7.16–7.12 (m, 2H), 7.01 (s, 1H), 6.58 (d, *J* = 4.80 Hz, 1H), 5.72 (brs, 2H), 5.16 (d, *J* = 4.80 Hz, 1H), 3.69 (s, 3H), 2.56 (q, *J* = 4.00 Hz, 2H), 2.39 (s, 3H), 0.99 (t, *J* = 3.60 Hz, 3H); ESI (*m*/*z*): 434 [$C_{24}H_{24}FN_5O_2$ + H]$^+$; HPLC (Method B) 98.7% (AUC), t_R = 8.35 min; Chiral SFC (Chiralcel OX-H, Method B) 99.2% (AUC), t_R = 4.04 min.

(*R*)-*N*-(4-(4-Amino-2,7-dimethyl-7*H*-pyrrolo[2,3-*d*]pyrimidin-5-yl)phenyl)-2-(3-fluorophenyl)-2-hydroxyacetamide (62)

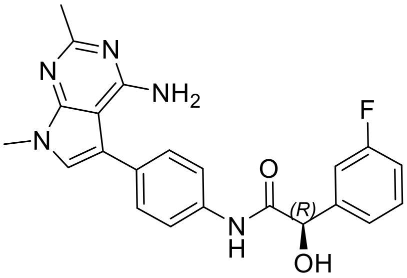

62

^{1}H NMR (400 MHz, DMSO-d_6) δ 10.08 (s, 1H), 7.78 (d, *J* = 8.0 Hz, 2H), 7.44–7.34 (m, 5H), 7.15 (s, 2H), 6.62 (d, *J* = 4.0 Hz, 1H), 5.91 (brs, 2H), 5.17 (d, *J* = 8.0 Hz, 1H), 3.68 (s, 3H), 2.40 (s, 3H); ESI (*m*/*z*): 406 [$C_{22}H_{20}FN_5O_2$ + H]$^+$; HPLC (Method B) 93.4% (AUC), t_R = 7.90 min.; Chiral SFC (Chiralpak IA, Method A) 97.6% (AUC), t_R = 7.09 min.

(*R*)-*N*-(4-(4-Amino-2,7-dimethyl-7*H*-pyrrolo[2,3-*d*]pyrimidin-5-yl)phenyl)-2-hydroxy-2-(3-(trifluoromethyl)phenyl)acetamide (63)

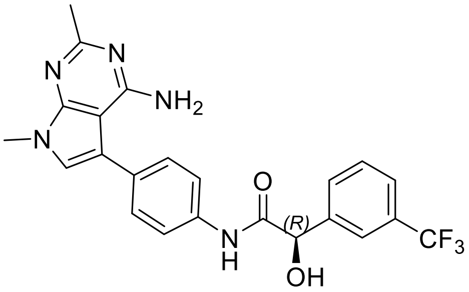

63

^{1}H NMR (400 MHz, DMSO-d_6) δ 10.11 (s, 1H), 7.91 (s, 1H), 7.84 (d, *J* = 7.60 Hz, 1H), 7.78 (d, *J* = 8.40 Hz, 2H), 7.69–7.60 (m, 2H), 7.36 (dd, *J* = 4.00, 12.00 Hz, 2H), 7.15 (s, 1H), 6.72 (d, *J* = 4.80 Hz, 1H), 5.94 (brs, 2H), 5.28 (d, *J* = 4.80 Hz, 1H), 3.68 (s, 3H), 2.41 (s, 3H); ESI (*m*/*z*): 456 [$C_{23}H_{20}F_3N_5O_2$ + H]$^+$; HPLC (Method A) > 99% (AUC), t_R = 3.37 min; Chiral SFC (Chiralcel OX-H, Method B) > 99 % (AUC), t_R = 3.36 min.

(*R*)-*N*-(4-(4-Amino-2,7-dimethyl-7*H*-pyrrolo[2,3-*d*]pyrimidin-5-yl)phenyl)-2-(3-bromophenyl)-2-hydroxyacetamide (64)

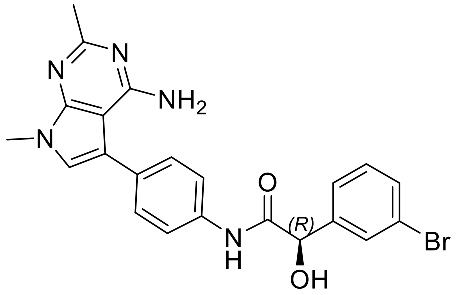

64

^{1}H NMR (400 MHz, DMSO-d_6) δ 10.05 (s, 1H), 7.78 (d, *J* = 8.40 Hz, 2H), 7.74 (s, 1H), 7.54–7.49 (m, 2H), 7.37–7.32 (m, 3H), 7.15 (s, 1H), 6.61 (d, *J* = 4.80 Hz, 1H), 5.94 (brs, 2H), 5.16 (d, *J* = 4.40 Hz, 1H), 3.68 (s, 3H), 2.41 (s, 3H); ESI (*m/z*): 466 [$C_{22}H_{20}BrN_5O_2$ + H]$^+$; HPLC (Method A) 97.9 % (AUC), t_R = 3.22 min; Chiral SFC (ChiralPak IB, Method D) >99 % (AUC), t_R = 2.63 min.

Synthesis of Phenyl (4-(4-Amino-7-methyl-7*H*-pyrrolo[2,3-*d*]pyrimidin-5-yl)-3-methylphenyl)carbamate (18)

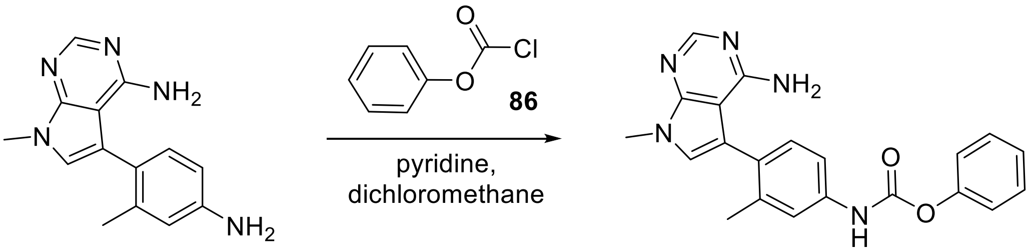

83 **18**

To a solution of 5-(4-amino-2-methylphenyl)-7-methyl-7*H*-pyrrolo[2,3-*d*]pyrimidin-4-amine (**83**, 200 mg, 0.79 mmol) in methylene chloride (15 mL) were added pyridine (124 mg, 1.58 mmol) followed by phenyl chloroformate (**86**, 1.18 mL, 0.94 mmol) at 0 °C. The resulting reaction mixture was allowed to warm to room temperature and stirred for 1 h. After this, the reaction mixture was concentrated under vacuum to afford crude product, which was purified by combi flash column chromatography (5% methanol in methylene chloride as an eluent) to provide phenyl (4-(4-amino-7-methyl-7*H*-pyrrolo[2,3-*d*]pyrimidin-5-yl)-3-methylphenyl)carbamate (**18**, 90 mg, 40%). ^{1}H NMR (400 MHz, DMSO-d_6) δ 10.31 (s, 1H), 8.13 (s, 1H), 7.42–7.50 (m, 4H), 7.15–7.29 (m, 5H), 5.76 (br s, 2H), 3.74 (s, 3H), 2.17 (s, 3H); ESI (*m/z*): 374 [$C_{21}H_{19}N_5O_2$ + H]$^+$.

Synthesis of 3-(4-(4-Amino-7-methyl-7*H*-pyrrolo[2,3-*d*]pyrimidin-5-yl)-3-methylphenyl)-1-methyl-1-phenylurea (20)

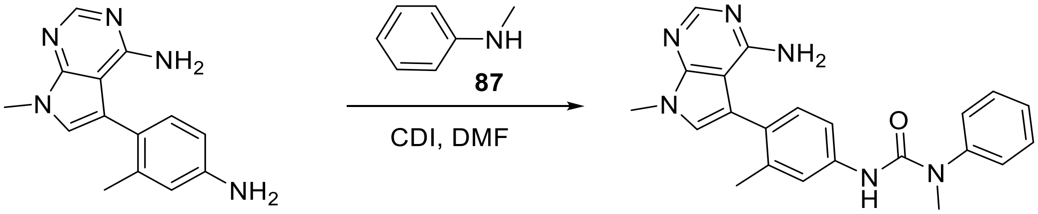

83 **20**

To a solution of 5-(4-amino-2-methylphenyl)-7-methyl-7*H*-pyrrolo[2,3-*d*]pyrimidin-4-amine (**83**, 150 mg, 0.59 mmol) and *N*-methyl aniline (**87**, 0.95 g, 0.89 mmol) in *N,N*-dimethylformamide (10 mL) was added 1,1'-carbonyldiimidazole (CDI) (192 mg, 1.18 mmol) at room temperature, and the mixture was stirred for 16 h. After this time, the reaction mixture was quenched with water (20 mL). The resulting solid was isolated by filtration and dried. The crude material was purified by combi flash column chromatography (5% methanol in methylene chloride as an eluent) to afford 3-(4-(4-amino-7-methyl-7*H*-pyrrolo[2,3-*d*]pyrimidin-5-yl)-3-methylphenyl)-1-methyl-1-phenylurea (**20**). ^{1}H NMR (400 MHz, DMSO-d_6) δ 8.19 (s, 1H), 8.12 (s, 1H), 7.33–7.44 (m, 6H), 7.24–7.28 (m, 1H), 7.08–7.12 (m, 2H), 5.76 (br s, 2H), 3.73 (s, 3H), 3.28 (s, 3H), 2.12 (s, 3H); ESI (*m/z*): 387 [$C_{22}H_{22}N_6O$ + H]$^+$.

The following compounds were prepared by following the same procedure for synthesizing **20**:

1-(4-(4-Amino-7-methyl-7*H*-pyrrolo[2,3-*d*]pyrimidin-5-yl)-3-methylphenyl)-3-phenylurea (19)

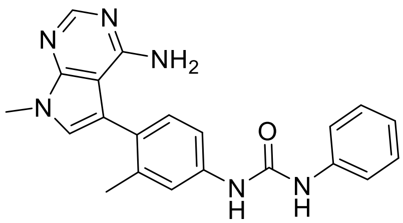

19

^{1}H NMR (400 MHz, DMSO-d_6) δ 8.69 (d, J = 4.00 Hz, 2H), 8.13 (s, 1H), 7.47–7.44 (m, 3H), 7.36 (dd, J = 8.00 Hz, 1H), 7.28 (t, J = 8.00 Hz, 2H), 7.16 (s, 1H), 7.13 (s, 1H), 6.97 (t, J = 8.00 Hz, 1H), 5.75 (s, 2H), 3.74 (s, 3H), 2.17 (s, 3H); ESI (m/z): 373 [$C_{21}H_{20}N_6O$ + H]$^+$; HPLC (Method C) 98.3% (AUC), t_R = 6.55 min.

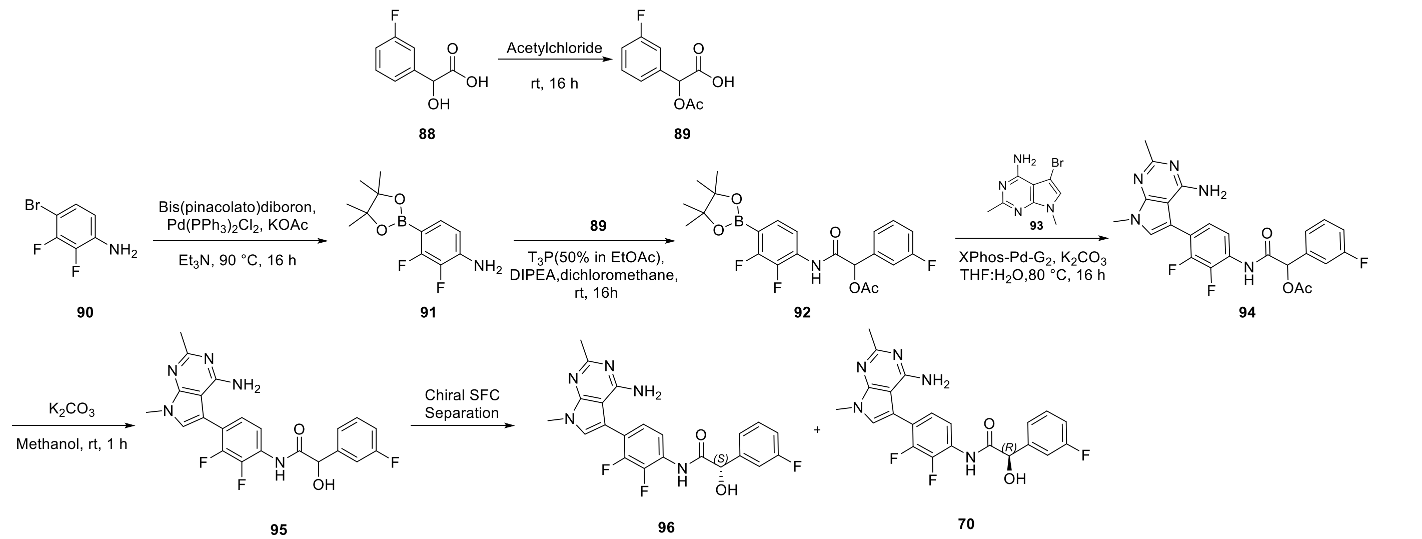

Scheme S2. Synthesis of (*S*)-*N*-(4-(4-amino-2,7-dimethyl-7*H*-pyrrolo[2,3-*d*]pyrimidin-5-yl)-2,3-difluoro phenyl)-2-(3-fluorophenyl)-2-hydroxyacetamide (96) and (*R*)-*N*-(4-(4-amino-2,7-dimethyl-7*H*-pyrrolo[2,3-*d*]pyrimidin-5-yl)-2,3-difluorophenyl)-2-(3-fluoro phenyl)-2-hydroxyacetamide (70).

2-Acetoxy-2-(3-fluorophenyl)acetic acid (89):

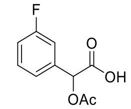

89

Acetyl chloride (40.0 mL) was treated with 2-(3-fluorophenyl)-2-hydroxyacetic acid (**88**, 20.0 g**,** 118 mmol) portion wise over a period of 10 min. at 0 °C. The reaction mixture was allowed to warm to room temperature and stirred for 16 h. After this time, the reaction mixture was concentrated under reduced pressure. Co-distillation with hexane afforded 2-acetoxy-2-(3-fluorophenyl)acetic acid (**89**, 20.0 g, 85%) as a white solid. ESI (*m/z*): 213.1 $[C_{10}H_9FO_4 + H]^+$.

2,3-Difluoro-4-(4,4,5,5-tetramethyl-1,3,2-dioxaborolan-2-yl)aniline (91):

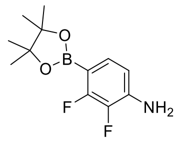

91

To a stirred solution of 4-bromo-2,3-difluoroaniline (**90**, 50.0 g, 241 mmol) in triethylamine (500 mL) under argon atmosphere were added bis(pinacolato)diboron (73.3 g, 289 mmol) followed by potassium acetate (70.8 g, 723 mmol). The resulting reaction mixture was purged with argon for 15 min. followed by addition of bis(triphenylphosphine)palladium(II) chloride (8.44 g, 12.0 mmol). After addition was complete, the resulting mixture was again purged with argon for 10 min. and heated at 90 °C with stirring for 16 h. After this time, the reaction mixture was allowed to cool to room temperature, passed through a bed of diatomaceous earth and washed with methylene chloride (3 × 500 mL). The filtrate was washed with water (2 × 500 mL) followed by brine solution (2 × 250 mL). The organic layer was separated, dried over anhydrous sodium sulfate and concentrated under reduced pressure. The crude material was purified by column chromatography (silica gel (100-200 mesh size), 10% ethyl acetate/hexanes as an eluent) to afford 2,3-difluoro-4-(4,4,5,5-tetramethyl-1,3,2-dioxaborolan-2-yl) aniline (**91**, 36.00 g, 62%) as a pale brown solid.
^{1}H NMR (400 MHz, DMSO-d_6) δ 7.10–7.06 (m, 1H), 6.54–6.50 (m, 1H), 5.92 (s, 2H), 1.25 (s, 12H); ESI (*m/z*): 256 $[C_{12}H_{16}BF_2NO_2 + H]^+$.

2-((2,3-Difluoro-4-(4,4,5,5-tetramethyl-1,3,2-dioxaborolan-2-yl)phenyl)amino)-1-(3-fluorophenyl)-2-oxoethyl Acetate (92):

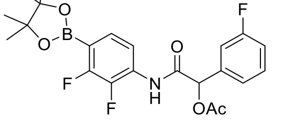

92

To a solution of 2,3-difluoro-4-(4,4,5,5-tetramethyl-1,3,2-dioxaborolan-2-yl)aniline (**91**, 14.0 g, 54.9 mmol) and 2-(3-fluorophenyl)-2-hydroxyacetic acid (**89**, 14.00 g, 65.88 mmol) in methylene chloride (280 mL) were added *N*,*N*-diisopropylethylamine (28.7 mL, 165 mmol) followed by drop-wise addition of propylphosphonic anhydride (T_3P) (50% in ethyl acetate) (52.3 mL, 82.4 mmol) at 0 °C. After addition was complete, the reaction mixture was allowed to warm to room temperature and stirred for 16 h. After this time, the reaction mixture was cooled to 0 °C, diluted with methylene chloride (100 mL) and washed with saturated sodium bicarbonate solution (100 mL). The organic layer was separated, washed with water (200 mL) and brine solution (200 mL), dried over anhydrous sodium sulfate and concentrated under reduced pressure. The crude material was purified by column chromatography (silica gel (100-200 mesh size), 10–20% ethyl acetate/hexanes as an eluent) to afford 2-((2,3-difluoro-4-(4,4,5,5-tetramethyl-1,3,2-dioxaborolan-2-yl)phenyl)amino)-1-(3-fluorophenyl)-2-oxoethyl acetate (**92**, 17.00 g, 69%) as pale brown viscous mass. ^{1}H NMR (400 MHz, DMSO-d_6) δ 7.70 (t, *J* = 7.2 Hz, 1H), 7.52–7.42 (m, 1H), 7.43–7.36 (m, 2H), 7.28–7.23 (m, 1H), 6.22 (s, 1H), 2.17 (s, 3H), 1.38 (s, 12H). ESI (*m/z*): 449 [$C_{22}H_{23}BF_3NO_5 + H]^+$.

2-((4-(4-Amino-2,7-dimethyl-7*H*-pyrrolo[2,3-*d*]pyrimidin-5-yl)-2,3-difluorophenyl)amino)-1-(3-fluorophenyl)-2-oxoethyl acetate (94):

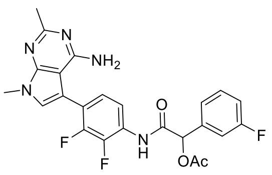

94

To a stirred solution of 5-bromo-2,7-dimethyl-7*H*-pyrrolo[2,3-*d*]pyrimidin-4-amine (**93**, 5.00 g, 20.8 mmol) in tetrahydrofuran (120 mL) and water (16 mL) were added 2-((2,3-difluoro-4-(4,4,5,5-tetramethyl-1,3,2-dioxaborolan-2-yl)phenyl)amino)-1-(3-fluorophenyl)-2-oxoethyl acetate (**5,** 12.15 g, 27.08 mmol) followed by potassium carbonate (8.62 g, 62.5 mmol), and the resulting reaction mixture was purged with argon for 10 min. Then, [2-(2-aminophenyl)phenyl]-chloro-palladium dicyclohexyl-[3-(2,4,6-triisopropylphenyl)phenyl]phosphane (XPhos-Pd-G_2) (1.63 g, 2.00 mmol) was added and the resulting reaction mixture was purged with argon for 10 min. The reaction mixture was heated to stirred at 80 °C for 4 h. After this, the reaction mixture was allowed to cool to room temperature, filtered through a bed of diatomaceous earth, and washed with ethyl acetate (2 × 100 mL). The filtrates were combined, washed with water (50 mL) and brine solution (50 mL). The organic layer was separated, dried over anhydrous sodium sulfate and concentrated under reduced pressure. The crude material was purified by combi flash column chromatography (5% methanol/methylene chloride as an eluent) to afford 2-((4-(4-amino-2,7-dimethyl-7*H*-pyrrolo[2,3-*d*]pyrimidin-5-yl)-2,3-difluorophenyl)amino)-1-(3-fluorophenyl)-2-oxoethyl acetate (**94**, 6.00 g, 60%) as an off white solid. ^{1}H NMR (400 MHz, DMSO-d_6) δ 10.48 (s, 1H), 7.69–7.66 (m, 1H), 7.52–7.38 (m, 3H), 7.27–7.23 (m, 2H), 7.14–7.09 (m, 1H), 6.23 (s, 1H), 6.08 (br s, 2H), 3.69 (s, 3H), 2.40 (s, 3H), 2.17 (s, 3H); ESI (*m/z*): 484 [$C_{24}H_{20}F_3N_5O_3 + H]^+$.

(*S*)-*N*-(4-(4-Amino-2,7-dimethyl-7*H*-pyrrolo[2,3-*d*]pyrimidin-5-yl)-2,3-difluorophenyl)-2-(3-fluorophenyl)-2-hydroxyacetamide (96) and (*R*)-*N*-(4-(4-amino-2,7-dimethyl-7*H*-pyrrolo[2,3-*d*]pyrimidin-5-yl)-2,3-difluorophenyl)-2-(3-fluorophenyl)-2-hydroxyacetamide (70):

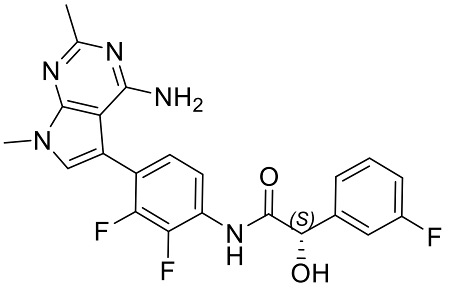

96 (Peak 1)

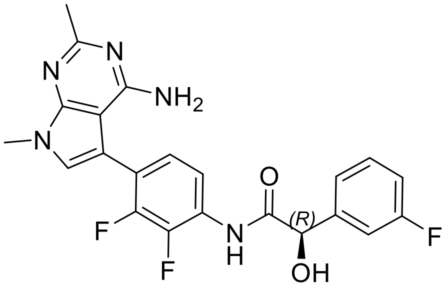

70 (Peak 2)

To a stirred solution of 2-((4-(4-amino-2,7-dimethyl-7*H*-pyrrolo[2,3-*d*]pyrimidin-5-yl)-2,3-difluorophenyl)amino)-1-(3-fluorophenyl)-2-oxoethyl acetate (**94**, 10.00 g, 20.70 mmol) in methanol (200 mL) was added potassium carbonate (3.42 g, 24.8 mmol), and the reaction mixture was stirred at ambient temperature for 1 h under argon. After this time, the reaction mixture was concentrated under vacuum to afford a viscous mass, which was diluted with water (250 mL). The resulting solid precipitate was isolated by filtration, washed with acetonitrile (50 mL) and dried under vacuum to afford racemic *N*-(4-(4-amino-2,7-dimethyl-7*H*-pyrrolo[2,3-*d*]pyrimidin-5-yl)-2,3-difluorophenyl)-2-(3-fluorophenyl)-2-hydroxyacetamide (**95**, 8.00 g, 88%) as an off white solid. ^{1}H NMR (400 MHz, DMSO-d_6) δ 9.98 (br s, 1H), 7.69–7.66 (m, 1H), 7.45–7.32 (m, 3H), 7.25 (s, 1H), 7.16–7.11 (m, 2H), 6.80 (br s, 1H), 6.06 (br s, 2H), 5.28 (s, 1H), 3.70 (s, 3H), 2.40 (s, 3H); ESI (*m/z*): 442 [$C_{22}H_{18}F_3N_5O_2 + H]^+$.
The mixture was combined with another batch of material and a total 30 g of racemate was separated into two pure enantiomers by chiral SFC (supercritical fluid chromatography) (Chiralcel OJ-H column, 0.3% DEA in methanol in CO_2, 40 °C temperature).

96 (Peak 1) (10.90 g) as an off-white solid. ^{1}H NMR (400 MHz, DMSO-d_6) δ 9.88 (s, 1H), 7.70–7.66 (m, 1H), 7.45–7.33 (m, 3H), 7.25 (s, 1H), 7.17–7.12 (m, 2H), 6.77 (br s, 1H), 6.07 (br s, 2H), 5.29 (br s, 1H), 3.70 (s, 3H), 2.41 (s, 3H); ESI (*m/z*) 442 [$C_{22}H_{18}F_3N_5O_2 + H]^+$; HPLC (Method B) 98.3% (AUC), t_R = 8.09 min; Chiral SFC (Chiralcel OJ-H, Method C) >99% (AUC), t_R = 4.67 min.

70 (Peak 2) (11.00 g) as off-white solid: ^{1}H NMR (400 MHz, DMSO-d_6) δ 9.87 (s, 1H), 7.69–7.65 (m, 1H), 7.45–7.32 (m, 3H), 7.25 (s, 1H), 7.17–7.12 (m, 2H), 6.76 (d, *J* = 5.2 Hz, 1H), 6.06 (br s, 2H), 5.29 (d, *J* = 4.8 Hz, 1H), 3.70 (s, 3H), 2.40 (s, 3H); ESI (*m/z*) 442 $[C_{22}H_{18}F_3N_5O_2 + H]^+$; HPLC (Method B) >99% (AUC), t_R = 8.09 min; Chiral SFC (Chiralcel OJ-H, Method C) >99% (AUC), t_R = 5.37 min.

The following compounds were prepared by following the same procedure for synthesizing **70**:

(*R*)-*N*-(4-(4-Amino-2,7-dimethyl-7*H*-pyrrolo[2,3-*d*]pyrimidin-5-yl)-3-methylphenyl)-2-hydroxy-2-(3-(trifluoromethyl) phenyl) acetamide (26):

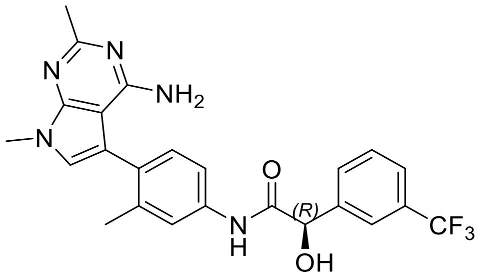

26

^{1}H NMR (400 MHz, DMSO-d_6) δ 10.06 (s, 1H), 7.91 (s, 1H), 7.84 (d, *J* = 7.60 Hz, 1H), 7.70–7.59 (m, 4H), 7.15 (d, *J* = 8.40 Hz, 1H), 7.03 (s, 1H), 6.72 (d, *J* = 5.20 Hz, 1H), 5.63 (brs, 2H), 5.28 (d, *J* = 4.80 Hz, 1H), 3.69 (s, 3H), 2.40 (s, 3H), 2.15 (s, 3H); ESI (*m/z*): 470 $[C_{24}H_{22}F_3N_5O_2 + H]^+$; HPLC (Method B) >99 % (AUC), t_R = 7.73 min; Chiral SFC (Chiralcel OX-H, Method B) >99 % (AUC), t_R = 2.57 min.

(*R*)-*N*-(4-(4-Amino-2,7-dimethyl-7*H*-pyrrolo[2,3-*d*]pyrimidin-5-yl)-3-methylphenyl)-2-(3-chlorophenyl)-2-hydroxyacetamide (28):

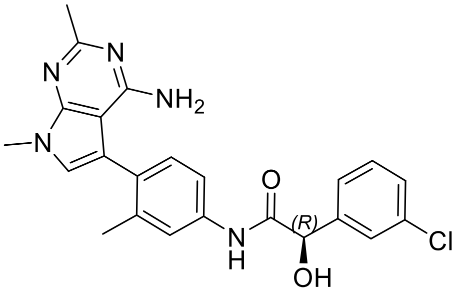

28

^{1}H NMR (400 MHz, DMSO-d_6) δ 9.99 (s, 1H), 7.67 (d, *J* = 1.60 Hz, 1H), 7.61–7.59 (m, 2H), 7.49 (d, *J* = 7.20 Hz, 1H), 7.42–7.36 (m, 2H), 7.15 (d, *J* = 8.00 Hz, 1H), 7.03 (s, 1H), 6.61 (d, *J* = 4.80 Hz, 1H), 5.62 (m, 2H), 5.16 (d, *J* = 4.80 Hz, 1H), 3.69 (s, 3H), 2.40 (s, 3H), 2.15 (s, 3H); ESI (*m/z*): 436 $[C_{23}H_{22}ClN_5O_2 + H]^+$; HPLC (Method B) 98.5% (AUC), t_R = 8.35 min; Chiral SFC (Chiralcel OX-H , Method B) >99% (AUC), t_R = 2.17 min.

(*R*)-*N*-(4-(4-Amino-2,7-dimethyl-7*H*-pyrrolo[2,3-*d*]pyrimidin-5-yl)-3-methylphenyl)-2-(3-(difluoromethyl) phenyl)-2-hydroxyacetamide (34):

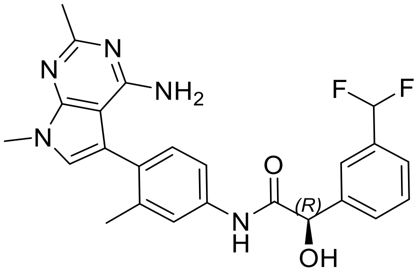

34

^{1}H NMR (400 MHz, DMSO-d_6) δ 10.00 (s, 1H), 7.74 (s, 1H), 7.69–7.64 (m, 2H), 7.60 (dd, *J* = 2.00, 8.00 Hz, 1H), 7.53–7.51 (m, 2H), 7.21–6.93 (m, 3H), 6.62 (d, *J* = 4.00 Hz, 1H), 5.76 (brs, 2H), 5.21 (d, *J* = 0.80 Hz, 1H), 3.68 (s, 3H), 2.39 (s, 3H), 2.14 (s, 3H); ESI (*m/z*): 452

[$C_{24}H_{23}F_2N_5O_2$ + H]$^+$; HPLC (Method A) 98.9% (AUC), t_R = 3.32 min; Chiral SFC (Chiralcel OX–H, Method B) 98.4% (AUC), t_R = 1.51 min.

(*R*)-*N*-(4-(4-Amino-2,7-dimethyl-7*H*-pyrrolo[2,3-*d*]pyrimidin-5-yl)-3-methylphenyl)-2-(3-cyclopropylphenyl)-2-hydroxyacetamide (35):

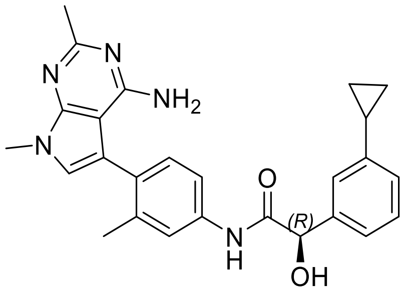

35

^{1}H NMR (400 MHz, DMSO-d_6) δ 9.90 (s, 1H), 7.68–7.67 (m, 1H), 7.60 (dd, J = 4.00 Hz, 8.00 Hz, 1H), 7.28–7.20 (m, 3H), 7.13 (d, J = 8.00 Hz, 1H), 7.02 (s, 1H), 6.98 (d, J = 8.00 Hz, 2H), 6.38 (d, J = 8.00 Hz, 1H), 5.63 (s, 2H), 5.06 (d, J = 4.00 Hz, 1H), 3.68 (s, 3H), 2.39 (s, 3H), 2.14 (s, 3H), 1.95–1.88 (m, 1H), 0.97–0.93 (m, 2H), 0.67–0.53 (m, 2H); ESI (*m/z*): 442 [$C_{26}H_{27}N_5O_2$ + H]$^+$; HPLC (Method A) 99.9% (AUC), t_R = 3.51 min; Chiral SFC (Chiralcel OJ-H, Method C) 97.2% (AUC), t_R = 5.18 min.

(*R*)-*N*-(4-(4-Amino-2,7-dimethyl-7*H*-pyrrolo[2,3-*d*]pyrimidin-5-yl)-3-methylphenyl)-2-(3-cyanophenyl)-2-hydroxyacetamide (36):

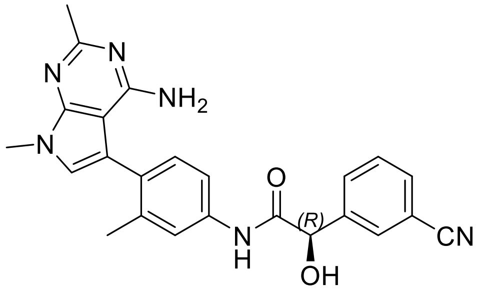

36

^{1}H NMR (400 MHz, DMSO-d_6) δ 9.99 (s, 1H), 7.96 (s, 1H), 7.87 (d, J = 7.60 Hz, 1H), 7.79 (d, J = 7.60 Hz, 1H), 7.66 (dd, J = 2.00 Hz, 1H), 7.62–7.58 (m, 2H), 7.15 (d, J = 8.00 Hz, 1H), 7.02 (s, 1H), 6.71 (d, J = 4.80 Hz, 1H), 5.62 (brs, 2H), 5.23 (d, J = 4.80 Hz, 1H), 3.69 (s, 3H), 2.40 (s, 3H), 2.15 (s, 3H); ESI (*m/z*): 427 [$C_{24}H_{22}N_6O_2$ + H]$^+$; HPLC (Method A) 97.2% (AUC), t_R = 2.95 min; Chiral SFC (ChiralPak IB , Method D) >99% (AUC), t_R = 4.14 min.

(*R*)-*N*-(4-(4-Amino-2,7-dimethyl-7*H*-pyrrolo[2,3-*d*]pyrimidin-5-yl)-3-methylphenyl)-2-hydroxy-2-(3-(methylsulfonyl)phenyl)acetamide (37):

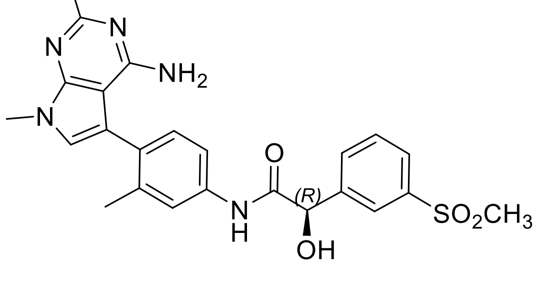

37

^{1}H NMR (400 MHz, DMSO-d_6) δ 8.12 (s, 1H), 7.89–7.87 (m, 2H), 7.68–7.64 (m, 2H), 7.61–7.59 (m, 1H), 7.15 (d, J = 8.00 Hz, 1H), 7.02 (s, 1H), 5.60 (brs, 2H), 5.29 (s, 1H), 3.69 (s, 3H), 3.23 (s, 3H), 2.40 (s, 3H), 2.15 (s, 3H); ESI (*m/z*): 480 [$C_{24}H_{25}N_5O_4S$ + H]$^+$; HPLC (Method A) 99.3% (AUC), t_R = 2.86 min; Chiral SFC (Chiralcel OX-H, Method B) 97.6% (AUC), t_R = 2.73 min.

(*R*)-*N*-(4-(4-Amino-2,7-dimethyl-7*H*-pyrrolo[2,3-*d*]pyrimidin-5-yl)-3-methylphenyl)-2-(3,5-difluorophenyl)-2-hydroxyacetamide (38):

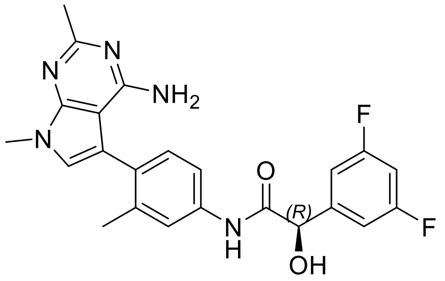

38

^{1}H NMR (400 MHz, DMSO-d6) δ 10.00 (s, 1H), 7.66 (d, *J* = 2.00 Hz, 1H), 7.60 (dd, *J* =2.00 , 8.20 Hz, 1H), 7.26–7.14 (m, 4H), 7.03 (s, 1H), 6.74 (d, *J* = 4.80 Hz, 1H), 5.66 (brs, 2H), 5.19 (d, *J* = 4.80 Hz, 1H), 3.69 (s, 3H), 2.40 (s, 3H), 2.15 (s, 3H) (one NH not appearing); ESI (*m/z*): 438 [$C_{23}H_{21}F_2N_5O_2$ + H]$^{++}$; HPLC (Method B) >99% (AUC), t_R = 8.11 min; Chiral SFC (Chiralcel OX-H, Method B) >99% (AUC), t_R = 2.39 min.

(*R*)-*N*-(4-(4-Amino-2,7-dimethyl-7*H*-pyrrolo[2,3-*d*]pyrimidin-5-yl)-3-methylphenyl)-2-(3-fluoro-5-(trifluoromethyl)phenyl)-2-hydroxyacetamide (39):

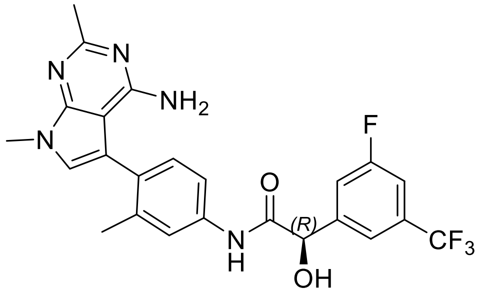

39

^{1}H NMR (400 MHz, DMSO-*d*$_6$) δ 10.07 (s, 1H), 7.78 (s, 1H), 7.70–7.58 (m, 4H), 7.15 (d, *J* = 8.40 Hz, 1H), 7.03 (s, 1H), 6.87 (d, *J* = 5.20 Hz, 1H), 5.54 (brs, 2H) 5.31 (d, *J* = 4.80 Hz, 1H), 3.69 (s, 3H), 2.40 (s, 3H), 2.15 (s, 3H); ESI (*m/z*): 488 [$C_{24}H_{21}F_4N_5O_2$ + H]$^+$; HPLC (Method B) >99% (AUC), t_R = 8.79 min; Chiral SFC (ChiralPak IB, Method D) >99% (AUC), t_R = 1.88 min.

(*R*)-*N*-(4-(4-Amino-2,7-dimethyl-7*H*-pyrrolo[2,3-*d*]pyrimidin-5-yl)-3-methylphenyl)-2-(3-chloro-5-fluorophenyl)-2-hydroxyacetamide (40):

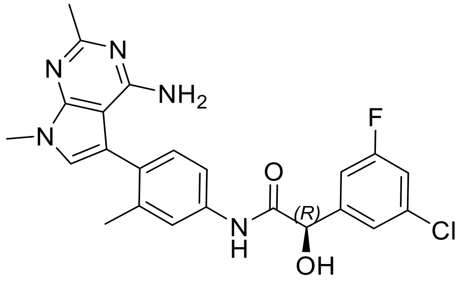

40

^{1}H NMR (400 MHz, DMSO–*d*$_6$) δ 9.98 (s, 1H), 7.66 (d, *J* = 1.60 Hz, 1H), 7.59 (dd, *J* = 2.00 , 8.40 Hz, 1H), 7.46 (s, 1H), 7.40–7.34 (m, 2H), 7.15 (d, *J* = 8.00 Hz, 1H), 7.02 (s, 1H), 6.73 (d, *J* = 4.80 Hz, 1H), 5.58 (brs, 2H), 5.19 (d, *J* = 5.20 Hz, 1H), 3.69 (s, 3H), 2.40 (s, 3H), 2.15 (s, 3H); ESI (*m/z*): 454 [$C_{23}H_{21}ClFN_5O_2$ + H]$^+$; HPLC (Method A) 97.6% (AUC), t_R = 3.46 min; Chiral SFC (Chiralcel OX–H, Method B) 97.7% (AUC), t_R = 1.25 min

(*R*)-*N*-(4-(4-Amino-2,7-dimethyl-7*H*-pyrrolo[2,3-*d*]pyrimidin-5-yl)-3-methylphenyl)-2-(3-fluoro-5-methylphenyl)-2-hydroxyacetamide (41):

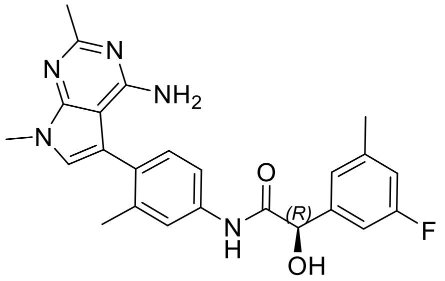

41

^{1}H NMR (400 MHz, DMSO-d_6) δ 9.92 (brs, 1H), 7.66 (s, 1H), 7.60 (d, *J* = 8.00 Hz, 1H), 7.18–7.13 (m, 3H), 7.01 (s, 1H), 6.96 (d, *J* = 12.00 Hz, 1H), 6.53 (brs, 1H), 5.60 (brs, 2H), 5.10 (s, 1H), 3.68 (s, 3H), 2.39 (s, 3H), 2.32 (s, 3H), 2.15 (s, 3H); ESI (*m/z*): 434 [$C_{24}H_{24}FN_5O_2$ + H]$^+$. HPLC (Method B) >99% (AUC), t_R = 8.28 min.

(*R*)-*N*-(4-(4-Amino-2,7-dimethyl-7*H*-pyrrolo[2,3-*d*]pyrimidin-5-yl)-2-fluoro-3-methylphenyl)-2-(3-fluorophenyl)-2-hydroxyacetamide (65):

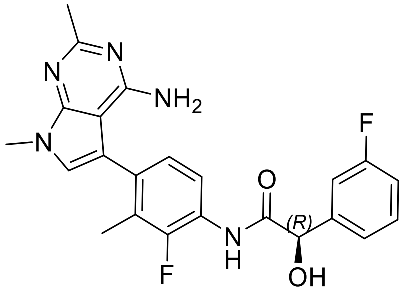

65

^{1}H NMR (400 MHz, DMSO-d_6) δ 9.62 (s, 1H), 7.82 (t, *J* = 8.00 Hz, 1H), 7.45–7.32 (m, 3H), 7.17–7.12 (m, 1H), 7.08 (s, 1H), 7.04 (d, *J* = 8.00 Hz, 1H), 6.82 (d, *J* = 4.80 Hz, 1H), 5.76 (brs, 2H), 5.28 (d, *J* = 4.80 Hz, 1H), 3.70 (s, 3H), 2.41 (s, 3H), 2.13 (d, *J* = 2.40 Hz, 3H); ESI (*m/z*): 438 [$C_{23}H_{21}F_2N_5O_2$ + H]$^+$; HPLC (Method A) >99% (AUC), t_R = 3.40 min; Chiral SFC (Chiralcel OD-H, Method F) >99% (AUC), t_R = 3.23 min.

(*R*)-*N*-(5-(4-Amino-2,7-dimethyl-7*H*-pyrrolo[2,3-*d*]pyrimidin-5-yl)-6-methylpyridin-2-yl)-2-(3-fluorophenyl)-2-hydroxyacetamide (72):

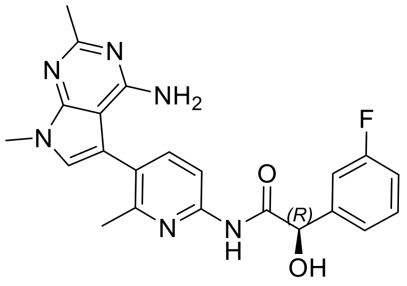

72

^{1}H NMR (400 MHz, DMSO-d_6) *δ* 10.13 (s, 1H), 7.92 (d, *J* = 8.4 Hz, 1H), 7.56 (d, *J* = 8.0 Hz, 1H), 7.44–7.33 (m, 3H), 7.15–7.10 (m, 1H), 7.09 (s, 1H), 6.57 (d, *J* = 6.0 Hz, 1H), 5.82 (brs, 2H), 5.30 (d, *J* = 5.0 Hz, 1H), 3.68 (s, 3H), 2.39 (s, 3H), 2.34 (s, 3H); ESI (*m/z*): 421 [$C_{22}H_{21}FN_6O_2$ + H]$^+$; HPLC (Method B) 96.7% (AUC), t_R = 7.19 min; Chiral SFC (ChiralPak OD-H, Method E) >99% (AUC), t_R = 3.50 min.

(*R*)-*N*-(4-(4-Amino-2,7-dimethyl-7*H*-pyrrolo[2,3-*d*]pyrimidin-5-yl)-3-methylphenyl)-2-(3-cyano-5-fluorophenyl)-2-hydroxyacetamide (42):

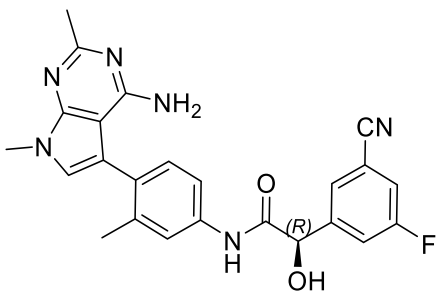

42

^{1}H NMR (400 MHz, DMSO-d_6) δ 10.01 (s, 1H), 7.86 (d, *J* = 5.20 Hz, 1H), 7.84–7.82 (m, 2H), 7.74 (dd, *J* = 1.60 , 9.60 Hz, 1H), 7.66 (d, *J* = 2.00 Hz, 1H), 7.15 (d, *J* = 8.00 Hz, 1H), 7.02 (s, 1H), 6.84 (d, *J* = 4.0 Hz, 1H), 5.68 (brs, 2H), 5.27 (d, *J* = 5.20 Hz, 1H), 3.69 (s, 3H), 2.40 (s, 3H), 2.15 (s, 3H); ESI (*m/z*): 445 $[C_{24}H_{21}FN_6O_2 + H]^+$; HPLC (Method A) >99% (AUC), t_R = 3.25 min; Chiral SFC (Chiralcel OD–H, Method E) >99% (AUC), t_R = 1.52 min.

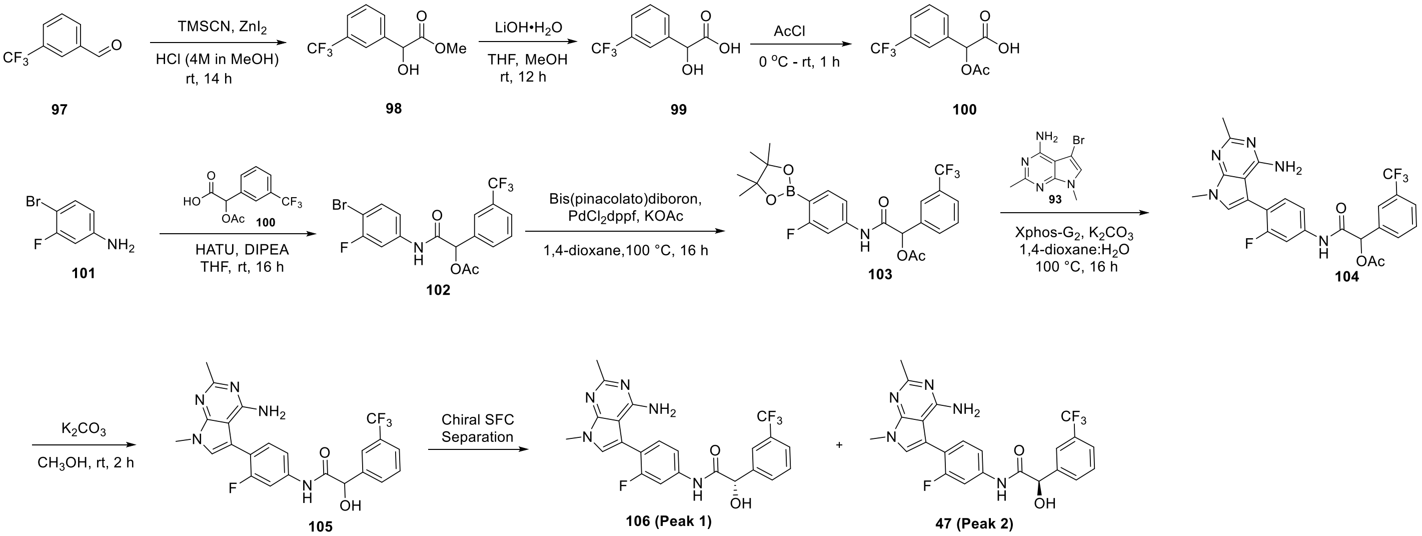

Scheme S3. Synthesis of (*S*)-*N*-(4-(4-amino-2,7-dimethyl-7*H*-pyrrolo[2,3-*d*]pyrimidin-5-yl)-3-fluorophenyl)-2-hydroxy-2-(3-(trifluoromethyl)phenyl)acetamide (106) and (*R*)-N-(4-(4-amino-2,7-dimethyl-7*H*-pyrrolo[2,3-*d*]pyrimidin-5-yl)-3-fluorophenyl)-2-hydroxy-2-(3-(trifluoromethyl) phenyl)acetamide (47).

Methyl 2-Hydroxy-2-(3-(trifluoromethyl)phenyl)acetate (98)

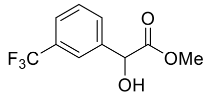

98

3-(Trifluoromethyl)benzaldehyde (**97**, 25.0 g, 143 mmol) was treated with zinc iodide (4.50 g, 14.3 mmol) at 0 °C followed by the drop wise addition of trimethylsilyl cyanide (17.0 mL, 172.0 mmol). The resulting reaction mixture was stirred at 0 °C for 2 h. After this time, hydrogen chloride (100 mL, 4N in methanol) was added at 0 °C. The reaction mixture was allowed to warm to room temperature and stirred for 12 h. After this, the reaction mixture was concentrated under reduced pressure. The crude product was treated with saturated sodium bicarbonate solution (100 mL) to adjust the pH to ~8, then ethyl acetate (200 mL) was added. The organic layer was washed with water (4 × 200 mL) followed by brine (200 mL). The organic layer was separated, dried over anhydrous sodium sulfate and concentrated under reduced pressure to afford methyl 2-hydroxy-2-(3-(trifluoromethyl)phenyl) acetate (**98**, 28 g, 83%) as a yellow liquid; ESI (*m/z*): 235 $[C_{10}H_9F_3O_3 + H]^-$.

2-Hydroxy-2-(3-(trifluoromethyl)phenyl)acetic acid (99)

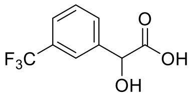

99

To a stirred solution of methyl 2-hydroxy-2-(3-(trifluoromethyl)phenyl)acetate (**98**, 28.0 g, 119 mmol) in tetrahydrofuran (70 mL), water (20 mL) and methanol (50 mL) was added lithium hydroxide hydrate (6.00 g, 143 mmol). The reaction mixture was stirred at room temperature for 12 h. After this time, the reaction mixture was concentrated under reduced pressure and diluted with water (100 mL). The aqueous mixture was washed with ethyl acetate (200 mL). The aqueous layer was acidified with hydrochloric acid (2N) to a pH of ~ 2 and extracted with ethyl acetate (2 x 150 mL). The Combined organic layers were dried over anhydrous sodium sulfate and concentrated under reduced pressure to afford 2-hydroxy-2-(3-(trifluoromethyl)phenyl)acetic acid (**99**, 25.00 g, 95%) as a colorless liquid. ESI (*m/z*): 219 $[C_9H_7F_3O_3 - H]^-$.

2-Acetoxy-2-(3-(trifluoromethyl)phenyl)acetic acid (100)

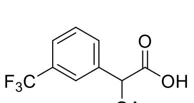

100

Acetyl chloride (50 mL) was treated with 2-hydroxy-2-(3-trifluoromethyl)phenyl)acetic acid (**99,** 25.0 g, 113 mmol) at 0 °C portion wise over a period of 30 min. The reaction mixture was allowed to warm at room temperature and stirred for 1 h. After this time, the reaction mixture was concentrated under reduced pressure and co-distilled with hexanes to afford 2-acetoxy-2-(3-(trifluoromethyl)phenyl)acetic acid

(**100**, 21.00 g, 70%) as a white solid. ^{1}H NMR (400 MHz, DMSO-d_6) δ 7.80 (t, *J* = 8.0 Hz, 3H), 7.68 (t, *J* = 8.0 Hz, 1H), 6.00 (s, 1H), 2.15 (s, 3H); ESI (*m/z*): 262 [$C_{11}H_9F_3O_4$ + H]$^+$.

2-((4-Bromo-3-fluoro phenyl)amino)-2-oxo-1-(3-(trifluoro methyl)phenyl)ethyl acetate (102)

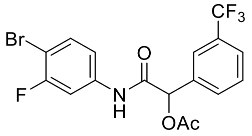

102

To a stirred solution of 4-bromo-3-fluoroaniline (**101**, 0.25 g, 1.3 mmol) and 2-acetoxy-2-(3-(trifluoromethyl)phenyl)acetic acid (**100**, 0.379 g, 1.45 mmol) in tetrahydrofuran (10 mL) was added *N*,*N*-diisopropylethylamine (0.339 g, 2.63 mmol) followed by 1-[bis(dimethylamino)methylene]-1*H*-1,2,3-triazolo[4,5-*b*]pyridinium 3-oxide hexafluorophosphate (HATU) (0.60 g, 1.6 mmol) at 0 °C. The reaction mixture was allowed to warm to room temperature and stirred for 16 h. After this time, the reaction mixture was cooled to 0 °C, diluted with water (20 mL), and extracted with ethyl acetate (2 × 20 mL). The organic layer was separated, washed with water (20 mL) and brine solution (20 mL), dried over anhydrous sodium sulfate and concentrated under reduced pressure. The crude material was purified by combi flash column chromatography (30% ethyl acetate/hexanes as an eluent) to afford 2-((4-bromo-3-fluorophenyl)amino)-2-oxo-1-(3-(trifluoromethyl)phenyl)ethyl acetate (**102,** 0.35 g, 62%) as pale-yellow solid. ESI (*m/z*): 434 [$C_{17}H_{12}BrF_4NO_3$ + H]$^+$.

2-((3-Fluoro-4-(4,4,5,5-tetramethyl-1,3,2-dioxaborolan-2-yl)phenyl)amino)-2-oxo-1-(3-(trifluoromethyl)phenyl)ethyl acetate (103)

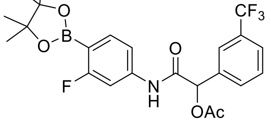

103

A mixture of 2-((4-bromo-3-fluorophenyl)amino)-2-oxo-1-(3-(trifluoromethyl)phenyl)ethyl acetate (**102,** 0.32 g, 0.74 mmol), bis(pinacolato)diboron (0.207 g, 0.814 mmol), and potassium acetate (0.218 g, 2.22 mmol) in 1,4-dioxane (6.5 mL) was degassed with argon for 10 min. Then, (1,1'-bis(diphenylphosphino)ferrocene)palladium(II) dichloride ($PdCl_2$dppf) (0.032 g, 0.044 mmol) was added, and the resulting reaction mixture was heated at 90 °C for 16 h. After this time, the reaction mixture was cooled to room temperature, passed through a bed of diatomaceous earth and washed with methylene chloride (3 × 10 mL). The filtrate was washed with water (2 × 10 mL) and brine solution (2 × 10 mL). The organic layer was separated, dried over anhydrous sodium sulfate and concentrated under reduced pressure. The crude product was purified by combi flash column chromatography (25% ethyl acetate/hexanes an eluent) to afford 2-((3-fluoro-4-(4,4,5,5-tetramethyl-1,3,2-dioxaborolan-2-yl)phenyl)amino)-2-oxo-1-(3-(trifluoromethyl)phenyl)ethyl acetate (**103**, 0.31 g, 87%) as a viscous mass. ESI (*m/z*): 482 [$C_{23}H_{24}BF_4NO_5$ + H]$^+$.

2-((4-(4-Amino-2,7-dimethyl-7*H*-pyrrolo[2,3-*d*]pyrimidin-5-yl)-3-fluorophenyl)amino)-2-oxo-1-(3-(trifluoromethyl)phenyl)ethyl acetate (104)

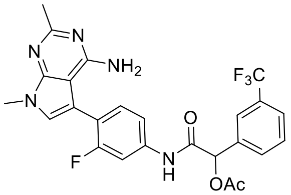

104

To a stirred solution of 5-bromo-2,7-dimethyl-7*H*-pyrrolo[2,3-*d*]pyrimidin-4-amine (**93**, 0.12 g, 0.50 mmol) in 1,4-dioxane (2 mL) and water (1 mL) were added 2-((3-fluoro-4-(4,4,5,5-tetramethyl-1,3,2-dioxaborolan-2-yl)phenyl)amino)-2-oxo-1-(3-(trifluoromethyl)phenyl)ethyl acetate (**103**, 0.263 g, 0.547 mmol) followed by potassium carbonate (0.274 g, 1.988 mmol). The resulting mixture was purged with argon for 10 min. Then, [2-(2-aminophenyl)phenyl]-chloro-palladium dicyclohexyl-[3-(2,4,6-triisopropylphenyl)phenyl]phosphane (XPhos-Pd-G_2) (0.023 g, 0.029 mmol) was added, and the mixture was purged with Argon for 10 min. The reaction mixture was heated at 100 °C for 16 h. After this time, the reaction mixture was allowed to cool to room temperature, filtered through a bed of diatomaceous earth and washed with ethyl acetate (10 mL). The filtrates were combined and washed with water (10 mL) and brine solution (10 mL). The organic layer was separated, dried over anhydrous sodium sulfate and concentrated under reduced pressure. The crude product was purified by combi flash column chromatography (3% methanol/methylene chloride as an eluent) to afford 2-((4-(4-amino-2,7-dimethyl-7*H*-pyrrolo[2,3-*d*]pyrimidin-5-yl)-3-fluorophenyl)amino)-2-oxo-1-(3-(trifluoromethyl)phenyl)ethyl acetate (**104**, 0.17 g, 66%) as pale-yellow solid. ESI (*m/z*): 516 [$C_{25}H_{21}F_4N_5O_3$ + H]$^+$.

(*S*)-*N*-(4-(4-Amino-2,7-dimethyl-7*H*-pyrrolo[2,3-*d*]pyrimidin-5-yl)-3-fluorophenyl)-2-hydroxy-2-(3-(trifluoro methyl)phenyl)acetamide (106) and (*R*)-*N*-(4-(4-amino-2,7-dimethyl-7*H*-pyrrolo[2,3-*d*]pyrimidin-5-yl)-3-fluorophenyl)-2-hydroxy-2-(3-(trifluoromethyl)phenyl)acetamide (47)

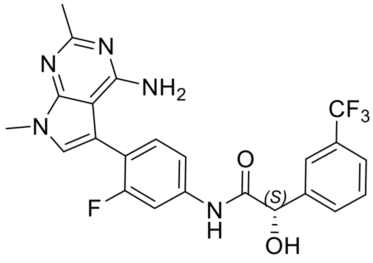

106

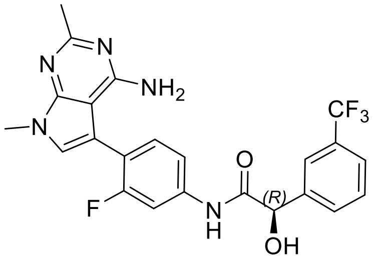

47

To a stirred solution of 2-((4-(4-amino-2,7-dimethyl-7*H*-pyrrolo[2,3-*d*]pyrimidin-5-yl)-3-fluorophenyl)amino)-2-oxo-1-(3-(trifluoromethyl)phenyl)ethyl acetate (**104**, 0.17 g, 0.33 mmol) in methanol (3.4 mL) was added potassium carbonate (0.91 g, 0.66 mmol), and the resulting reaction mixture was stirred at ambient temperature for 2 h under argon. After this time, the reaction mixture was concentrated under vacuum. The resulting crude material was diluted with methylene chloride (10 mL) and water (10 mL). The organic layer was separated, dried over anhydrous sodium sulfate and concentrated under reduced pressure to afford a pale yellow solid, which was purified by reverse phase column chromatography (acetonitrile 45–50% in water an eluent) to afford racemic *N*-(4-(4-amino-2,7-dimethyl-7*H*-pyrrolo[2,3-*d*]pyrimidin-5-yl)-3-fluorophenyl)-2-hydroxy-2-(3-(trifluoromethyl)phenyl)acetamide (**105**, 0.14 g) as a pale-yellow solid. ^{1}H NMR (400 MHz, DMSO-d_6) δ 10.34 (s, 1H), 7.91 (s, 1H), 7.83 (d, *J* = 8.00 Hz, 1H), 7.79 (dd, *J* = 4.00, 12.00 Hz, 1H), 7.69 (d, *J* = 8.00 Hz, 2H), 7.59–7.64 (m, 1H), 7.30 (t, *J* = 8.80 Hz, 1H), 7.17 (s, 1H), 6.82 (s, 1H), 5.92 (s, 2H), 5.29 (s, 1H), 3.69 (s, 3H), 2.40 (s, 3H); ESI (m/z): 474 $[C_{23}H_{19}F_4N_5O_2 + H]^+$.

The mixture of enantiomers (0.14 g) was separated into two pure enantiomers by SFC (supercritical fluid chromatography) (Chiralcel OJ-H column, 0.3% DEA in methanol in CO_2, 40 °C temperature).

106 (**Peak 1**) (0.038 g) as an off-white solid: ^{1}H NMR (400 MHz, DMSO-d_6) δ 10.33 (s, 1H), 7.90 (s, 1H), 7.83 (d, *J* = 8.00 Hz, 1H), 7.79 (dd, *J* = 4.00, 12.00 Hz, 1H), 7.69 (d, *J* = 8.00 Hz, 2H), 7.59–7.64 (m, 1H), 7.30 (t, *J* = 8.00 Hz, 1H), 7.17 (s, 1H), 6.80 (s, 1H), 5.90 (s, 2H), 5.20 (s, 1H), 3.69 (s, 3H), 2.40 (s, 3H); ESI (m/z): 474 $[C_{23}H_{19}F_4N_5O_2 + H]^+$; HPLC (Method B) >99% (AUC), t_R = 8.65 min; Chiral SFC (Chiralcel OJ-H, Method C) 97.2% (AUC), t_R = 1.49 min.

47 (**Peak 2**) (0.045 g) as an off-white solid. ^{1}H NMR (400 MHz, DMSO-d_6) δ 10.33 (s, 1H), 7.90 (s, 1H), 7.83 (d, *J* = 8.00 Hz, 1H), 7.79 (dd, *J* = 4.00, 12.00 Hz, 1H), 7.69 (d, *J* = 8.00 Hz, 2H), 7.59–7.64 (m, 1H), 7.30 (t, *J* = 8.00 Hz, 1H), 7.17 (s, 1H), 6.80 (s, 1H), 5.90 (s, 2H), 5.20 (s, 1H), 3.69 (s, 3H), 2.40 (s, 3H); ESI (m/z): 474 $[C_{23}H_{19}F_4N_5O_2 + H]^+$; HPLC (Method B) >99% (AUC), t_R = 8.65 min; Chiral SFC (Chiralcel OJ-H, Method C) 97.6% (AUC), t_R = 1.22 min.

The following compounds were prepared by following the same procedure for synthesizing **47**:

(*R*)-*N*-(4-(4-Amino-2,7-dimethyl-7*H*-pyrrolo[2,3-*d*]pyrimidin-5-yl)-3-methylphenyl)-2-hydroxy-2-(5-(trifluoromethyl)pyridin-3-yl)acetamide (45)

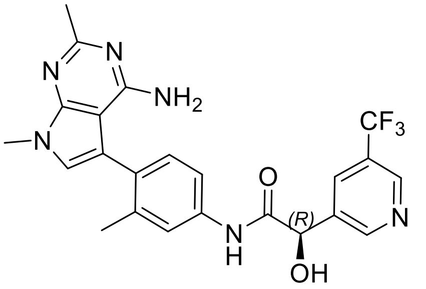

45

^{1}H NMR (400 MHz, DMSO-d_6) δ 10.10 (s, 1H), 9.03 (d, *J* = 1.20 Hz, 1H), 8.95 (d, *J* = 1.20 Hz, 1H), 8.29 (s, 1H), 7.63 (s, 1H), 7.60 (dd, *J* = 2.00 , 8.40 Hz, 1H), 7.03 (s, 1H), 7.16 (d, *J* = 8.40 Hz, 1H), 6.93 (d, *J* = 5.20 Hz, 1H), 5.62 (brs, 2H), 5.40 (d, *J* = 4.80 Hz, 1H), 3.69 (s, 3H), 2.40 (s, 3H), 2.15 (s, 3H); ESI (*m/z*): 471 $[C_{23}H_{21}F_3N_6O_2 + H]^+$; HPLC (Method A) 97% (AUC), t_R = 3.04 min; Chiral SFC (Chiralcel OX–H, Method B) 96.5% (AUC), t_R = 1.93 min.

(*R*)-*N*-(4-(4-Amino-2,7-dimethyl-7*H*-pyrrolo[2,3-*d*]pyrimidin-5-yl)-3-fluorophenyl)-2-(3-fluorophenyl)-2-hydroxyacetamide (46)

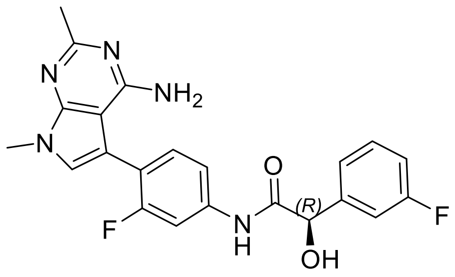

46

^{1}H NMR (400 MHz, DMSO-d_6) δ 10.26 (s, 1H), 7.80 (dd, *J* =2.00, 12.40 Hz, 1H), 7.61 (dd, *J* = 2.00, 8.40 Hz, 1H), 7.45–7.28 (m, 4H), 7.17–7.12 (m, 2H), 6.69 (d, *J* = 4.40 Hz, 1H), 5.94 (brs, 2H), 5.18 (d, *J* = 4.80 Hz, 1H), 3.69 (s, 3H), 2.41 (s, 3H); ESI (*m/z*): 424 [$C_{22}H_{19}F_2N_5O_2$ + H]$^+$; HPLC (Method B) 96.3% (AUC), t_R = 8.06 min; Chiral SFC (Chiralcel OX-H, Method B) 98.0% (AUC), t_R = 3.65 min.

(*R*)-*N*-(4-(4-Amino-2,7-dimethyl-7*H*-pyrrolo[2,3-*d*]pyrimidin-5-yl)-3-fluorophenyl)-2-(3-chlorophenyl)-2-hydroxyacetamide (48)

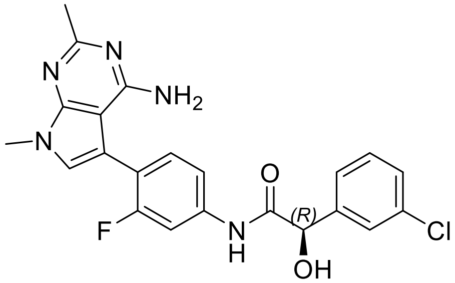

48

^{1}H NMR (400 MHz, DMSO-d_6) δ 10.24 (brs, 1H), 7.79 (dd, *J* = 2.00, 12.40 Hz, 1H), 7.61–7.59 (m, 2H), 7.49–7.47 (m, 1H), 7.42–7.35 (m, 2H), 7.30 (t, *J* = 8.00 Hz, 1H), 7.16 (s, 1H), 6.69 (brs, 1H), 5.89 (brs, 2H), 5.17 (s, 1H), 3.69 (s, 3H), 2.40 (s, 3H); ESI (*m/z*): 440 [$C_{22}H_{19}ClFN_5O_2$ + H]$^+$; HPLC (Method B) >99% (AUC), t_R = 8.32 min; Chiral SFC (Chiralcel OJ-H, Method C) >99% (AUC), t_R = 2.51 min.

(*R*)-*N*-(4-(4-Amino-2,7-dimethyl-7*H*-pyrrolo[2,3-*d*]pyrimidin-5-yl)-3-fluorophenyl)-2-hydroxy-2-(*m*-tolyl)acetamide (49)

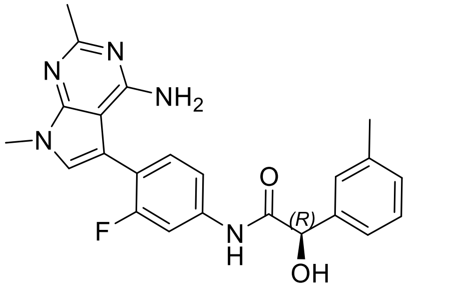

49

^{1}H NMR (400 MHz, DMSO-d_6) δ 10.17 (s, 1H), 7.80 (d, *J* = 12.40 Hz, 1H), 7.61 (d, *J* = 8.80 Hz, 1H), 7.33–7.24 (m, 4H), 7.11 (d, *J* = 6.8 Hz, 1H), 7.11 (d, *J* = 6.80 Hz, 1H), 6.47 (d, *J* = 4.40 Hz, 1H), 5.89 (brs, 2H), 5.08 (d, *J* = 4.80 Hz, 1H), 3.69 (s, 3H), 2.40 (s, 3H), 2.31 (s, 3H); ESI (*m/z*): 420 [$C_{23}H_{22}FN_5O_2$ + H]$^+$; HPLC (Method B) >99% (AUC), t_R = 8.11 min; Chiral SFC (Chiralcel OJ-H, Method C) >99% (AUC), t_R = 2.07 min.

(*R*)-*N*-(4-(4-Amino-2,7-dimethyl-7*H*-pyrrolo[2,3-*d*]pyrimidin-5-yl)-3-fluorophenyl)-2-(3-ethylphenyl)-2-hydroxyacetamide (50)

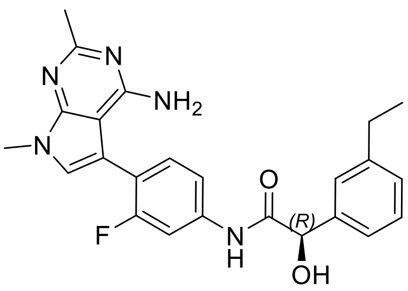

50

^{1}H NMR (400 MHz, DMSO-d_6) δ 10.19 (s, 1H), 7.81 (d, *J* = 14.40 Hz, 1H), 7.61 (d, *J* =10.40 Hz, 1H), 7.37 (s, 1H), 7.32–7.27 (m, 3H), 7.15 (dd, , *J* = 7.60 , Hz, 2H), 5.89 (brs, 2H), 5.09 (d, 1H), 3.69 (s, 3H), 2.61 (m, 2H), 2.40 (s, 3H), 1.19 (t, , *J* = 7.60 Hz, 3H); ESI (*m/z*): 434 [$C_{24}H_{24}FN_5O_2$ + H]$^+$; HPLC (Method B) 97.7% (AUC), t_R = 8.99 min; Chiral SFC (Chiralcel OJ-H, Method C) >99% (AUC), t_R = 2.52 min.

(*R*)-*N*-(4-(4-Amino-2,7-dimethyl-7*H*-pyrrolo[2,3-*d*]pyrimidin-5-yl)-3-fluorophenyl)-2-(3-fluoro-5-(trifluoromethyl)phenyl)-2-hydroxyacetamide (51)

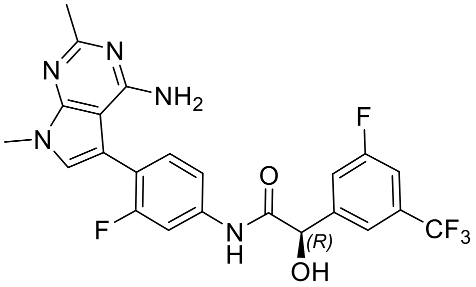

51

^{1}H NMR (400 MHz, DMSO-d_6) δ 10.32 (s, 1H), 7.79–7.76 (m, 2H), 7.69–7.58 (m, 3H), 7.31 (t, *J* = 8.00 Hz, 1H), 7.16 (s, 1H), 6.95 (s, 1H), 5.89 (s, 2H), 5.33 (s, 1H), 3.69 (s, 3H), 2.40 (s, 3H); ESI (*m/z*): 492 [$C_{23}H_{18}F_5N_5O_2$ + H]$^+$; HPLC (Method B) 98.5% (AUC), t_R = 8.87 min; Chiral SFC (Chiralcel OX–H, Method B) 97.9% (AUC), t_R = 1.71 min.

(*R*)-*N*-(4-(4-Amino-2,7-dimethyl-7*H*-pyrrolo[2,3-*d*]pyrimidin-5-yl)-3-fluorophenyl)-2-(3-fluoro-5-methylphenyl)-2-hydroxyacetamide (52)

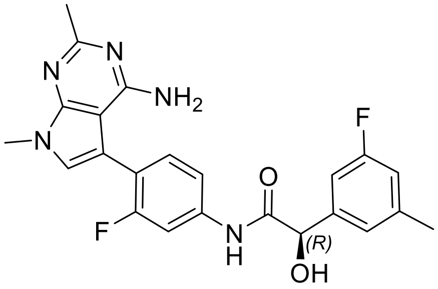

52

^{1}H-NMR (400 MHz, DMSO-d_6) *δ*: 10.24 (brs, 1H), 7.79 (dd, *J* = 2.00 , 12.40 Hz, 1H), 7.60 (dd, *J* = 2.00, 8.40 Hz, 1H), 7.30 (t, *J* = 8.80 Hz, 1H), 7.18–7.12 (m, 3H), 6.96 (d, *J* = 10.00 Hz, 1H), 6.69 (brs, 1H), 5.89 (brs, 2H), 5.12 (s, 1H), 3.69 (s, 3H), 2.40 (s, 3H), 2.33 (s, 3H); ESI (m/z): 438 [$C_{23}H_{21}F_2N_5O_2$+H]$^+$; HPLC (Method B) >99% (AUC), t_R = 8.29 min; Chiral SFC (Chiralcel OX-H, Method B) >99% (AUC), t_R = 4.29 min.

(*R*)-*N*-(4-(4-Amino-2,7-dimethyl-7*H*-pyrrolo[2,3-*d*]pyrimidin-5-yl)-3-chlorophenyl)-2-(3-fluorophenyl)-2-hydroxyacetamide (53)

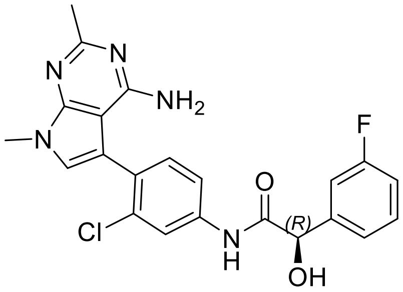

53

^{1}H NMR 400 MHz, DMSO-d_6) δ 10.22 (s, 1H), 8.04 (d, *J* = 2.0 Hz, 1H), 7.73 (dd, *J* = 8.0, 4.0 Hz, 1H), 7.44–7.30 (m, 4H), 7.13 (s, 2H), 6.67 (d, *J* = 4.0 Hz, 1H), 5.71 (brs, 2H), 5.18 (d, *J* = 4.0 Hz, 1H), 3.68 (s, 3H), 2.39 (s, 3H); ESI (*m/z*): 440 [$C_{22}H_{19}ClFN_5O_2$ + H]$^+$; HPLC (Method B) 94.5% (AUC), t_R = 8.33 min.; Chiral SFC (Chiralcel OJ-H, Method C) 97.2% (AUC), t_R = 6.39 min.

(*R*)-*N*-(4-(4-Amino-2,7-dimethyl-7*H*-pyrrolo[2,3-*d*]pyrimidin-5-yl)-3-chlorophenyl)-2-hydroxy-2-(3-(trifluoromethyl)phenyl)acetamide (54)

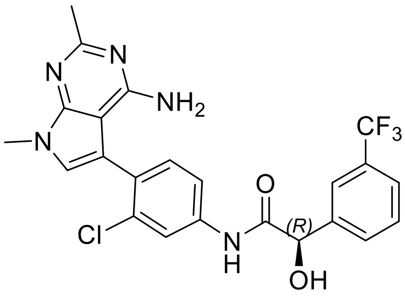

54

^{1}H NMR (400 MHz, DMSO-d_6) δ 10.3 (brs, 1H), 8.05 (d, *J* = 4.0 Hz, 1H), 7.90 (s, 1H), 7.83 (d, *J* = 8.0 Hz, 1H), 7.74–7.64 (m, 3H), 7.31 (d, *J* = 8.0 Hz, 1H), 7.12 (s, 1H), 6.81 (brs, 1H), 5.81 (brs, 2H), 5.28 (s, 1H), 3.68 (s, 3H), 2.39 (s, 3H); ESI (*m/z*): 490 [$C_{23}H_{19}ClF_3N_5O_2$ + H]$^+$; HPLC (Method B) 98.7% (AUC), t_R = 8.77 min; Chiral SFC (ChiralPak OD-H, Method E) 98.2% (AUC), t_R = 3.15 min.

(*R*)-*N*-(4-(4-Amino-2,7-dimethyl-7*H*-pyrrolo[2,3-*d*]pyrimidin-5-yl)-3-chlorophenyl)-2-(3-chlorophenyl)-2-hydroxyacetamide (55)

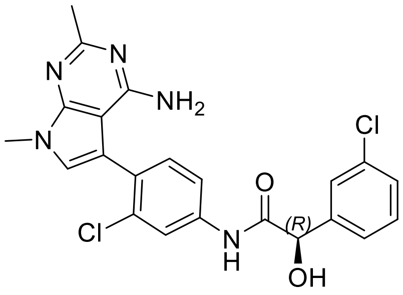

55

^{1}H NMR (400 MHz, DMSO-d_6) δ 10.26 (s, 1H), 8.05 (d, *J* = 2.00 Hz, 1H), 7.73 (dd, *J* =2.00 , 8.40 Hz, 1H), 7.59 (s, 1H), 7.50–7.48 (m, 1H), 7.43–7.37 (m, 2H), 7.31 (d, *J* = 8.40 Hz, 1H), 7.13 (s, 1H), 6.71 (d, *J* = 4.40 Hz, 1H), 5.76 (brs, 2H), 5.17 (d, *J* = 4.40 Hz, 1H), 3.69 (s, 3H), 2.40 (s, 3H); ESI (*m/z*): 456 [$C_{22}H_{19}Cl_2N_5O_2$ + H]$^+$; HPLC (Method B) 98.6% (AUC), t_R = 9.03 min; Chiral SFC (Chiralcel OX-H, Method B) >99% (AUC), t_R = 6.40 min.

(*R*)-*N*-(4-(4-Amino-2,7-dimethyl-7*H*-pyrrolo[2,3-*d*]pyrimidin-5-yl)-3-chlorophenyl)-2-hydroxy-2-(*m*-tolyl)acetamide (56)

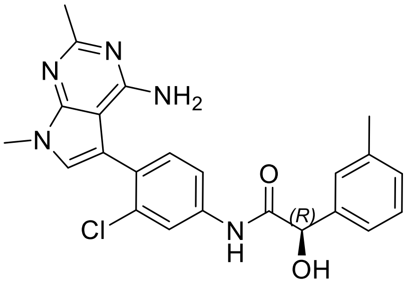

56

^{1}H NMR (400 MHz, DMSO-d_6) δ 10.19 (s, 1H), 8.07 (d, *J* = 2.00 Hz, 1H), 7.74 (dd, *J* = 2.40, 8.60 Hz, 1H), 7.34–7.32 (m, 4H), 7.12 (d, *J* = 2.40 Hz, 2H), 6.49 (d, *J* = 4.40 Hz, 1H), 5.76 (s, 2H), 5.08 (s, 1H), 3.69 (s, 3H), 2.40 (s, 3H), 2.32 (s, 3H); ESI (*m/z*): 436 [$C_{23}H_{22}ClN_5O_2$ + H]$^+$; HPLC (Method B) 97.21% (AUC), t_R = 8.29 min; Chiral SFC (Chiralcel OJ-H, Method C) 97.98% (AUC), t_R = 2.93 min.

(*R*)-*N*-(4-(4-Amino-2,7-dimethyl-7*H*-pyrrolo[2,3-*d*]pyrimidin-5-yl)-3-chlorophenyl)-2-(3-ethylphenyl)-2-hydroxyacetamide (57)

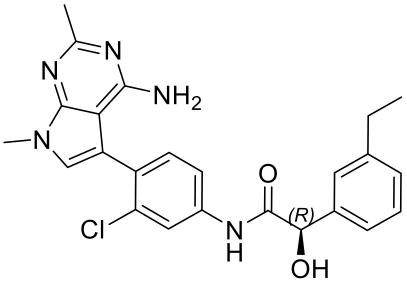

57

^{1}H NMR (400 MHz, DMSO-d_6) δ 10.18 (s, 1H), 8.06 (d, *J* = 2.00 Hz, 1H), 7.74 (dd, *J* =10.40 , Hz, 1H), 7.35 (d, *J* =14.00 Hz, 1H), 7.27 (dd, *J* =18.80 , Hz, 3H), 7.16–7.12 (m, 2H), 6.47 (s, 1H), 5.74 (s, 2H),), 5.10 (s, 1H), 3.69 (s, 3H), 2.67–2.59 (m, 2H), 2.40 (s, 3H), 1.19 (t, *J* =7.60 Hz, 3H); ESI (*m/z*): 450 [$C_{24}H_{24}ClN_5O_2$ + H]$^+$, HPLC (Method B) >99 (AUC), t_R = 9.15 min; Chiral SFC (Chiralcel OD-H, Method F) 95.7% (AUC), t_R = 6.18 min.

(*R*)-*N*-(4-(4-Amino-2,7-dimethyl-7*H*-pyrrolo[2,3-*d*]pyrimidin-5-yl)-3-(trifluoromethyl) phenyl)-2-(3-fluoro phenyl)-2-hydroxyacetamide (58)

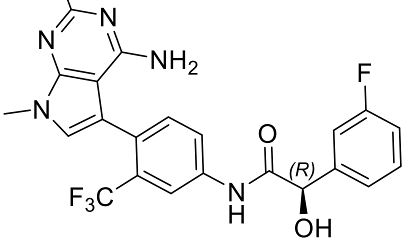

58

^{1}H NMR (400 MHz, DMSO-d_6) δ 10.41 (s, 1H), 8.32 (d, *J* = 1.60 Hz, 1H), 8.03 (d, *J* = 8.40 Hz, 1H), 7.45–7.34 (m, 4H), 7.17–7.12 (m, 1H), 7.03 (s, 1H), 6.72 (d, *J* = 4.80 Hz, 1H), 5.67 (brs, 2H), 5.20 (d, *J* = 4.40 Hz, 1H), 3.68 (s, 3H), 2.40 (s, 3H); ESI (*m/z*): 474 [$C_{23}H_{19}F_4N_5O_2$ + H]$^+$; HPLC (Method B) >99 % (AUC), t_R = 8.47 min; Chiral SFC (Chiralcel OJ-H, Method C) >99% (AUC), t_R = 4.11 min.

(*R*)-*N*-(4-(4-Amino-2,7-dimethyl-7*H*-pyrrolo[2,3-*d*]pyrimidin-5-yl)-3-methoxyphenyl)-2-(3-fluorophenyl)-2-hydroxyacetamide (60)

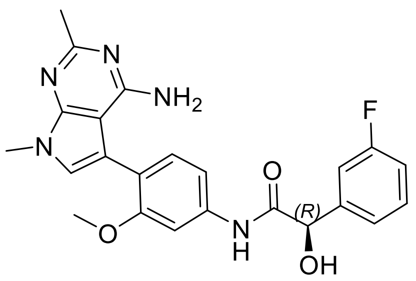

60

^{1}H NMR (400 MHz, DMSO-d_6) δ 10.06 (brs, 1H), 7.59 (d, *J* = 2.00 Hz, 1H), 7.44–7.34 (m, 4H), 7.16–7.11 (m, 2H), 7.01 (s, 1H), 6.65 (brs, 1H), 5.75 (brs, 2H), 5.17 (s, 1H), 3.70 (s, 3H), 3.66 (s, 3H), 2.38 (s, 3H); ESI (*m/z*): 436 [$C_{23}H_{22}FN_5O_3$ + H]$^+$; HPLC (Method B) 97.8% (AUC), t_R = 8.07 min; Chiral SFC (Chiralcel OJ-H, Method C) 98.8% (AUC), t_R = 4.05 min.

(*R*)-*N*-(4-(4-Amino-2,7-dimethyl-7*H*-pyrrolo[2,3-*d*]pyrimidin-5-yl)-3-(trifluoromethoxy)phenyl)-2-(3-fluorophenyl)-2-hydroxyacetamide (61)

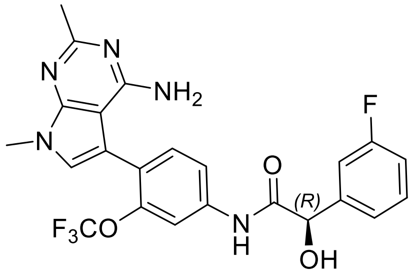

61

^{1}H NMR (400 MHz, DMSO-d_6) δ 10.35 (brs, 1H), 8.05 (s, 1H), 7.79 (dd, *J* = 1.60 , 8.40 Hz, 1H), 7.45–7.34 (m, 4H), 7.17–7.11 (m, 2H), 6.68 (s, 1H), 5.81 (brs, 2H), 5.51 (d, *J* = 4.4 Hz, 1H), 3.69 (s, 3H), 2.40 (s, 3H); ESI (*m/z*): 490 [$C_{23}H_{19}F_4N_5O_3$ + H]$^+$; HPLC (Method B) 98.0% (AUC), t_R = 8.61 min; Chiral SFC (Chiralcel OJ-H, Method C) >99% (AUC), t_R = 2.66 min.

(*R*)-*N*-(4-(4-Amino-2,7-dimethyl-7*H*-pyrrolo[2,3-*d*]pyrimidin-5-yl)-2-fluoro-5-methylphenyl)-2-(3-fluorophenyl)-2-hydroxyacetamide (66)

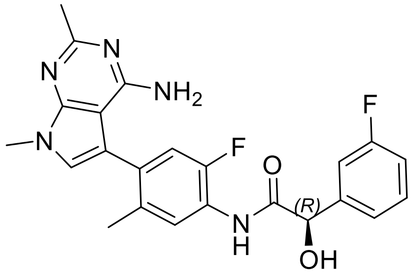

66

^{1}H NMR (400 MHz, DMSO-d_6) δ 9.64 (s, 1H), 7.82 (d, *J* = 8.40 Hz, 1H), 7.43–7.32 (m, 3H), 7.17–7.09 (m, 3H), 6.80 (brs, 1H), 5.73 (brs, 2H), 5.26 (s, 1H), 3.69 (s, 3H), 2.40 (s, 3H), 2.13 (s, 3H); ESI (*m/z*): 438 [$C_{23}H_{21}F_2N_5O_2$ + H]$^+$: HPLC (Method B) 98.0% (AUC), t_R = 8.17 min; Chiral SFC (Chiralpak IA, Method A) 98.8% (AUC), t_R = 3.56 min.

***N*-(4-(4-Amino-2,7-dimethyl-7*H*-pyrrolo[2,3-*d*]pyrimidin-5-yl)-3-fluoro-5-methylphenyl)-2-(3-fluorophenyl)-2-hydroxyacetamide (67)**

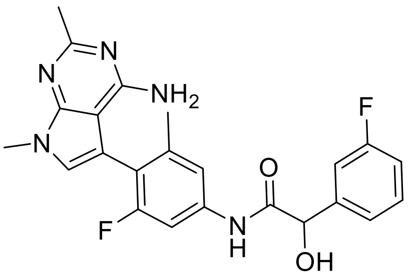

67

^{1}H NMR (400 MHz, DMSO-d_6) δ 10.13 (brs, 1H), 7.62–7.58 (m, 1H), 7.50 (s, 1H), 7.44–7.35 (m, 3H), 7.16–7.11 (m, 1H), 7.04 (s, 1H), 6.66–6.64 (m, 1H), 5.62 (brs, 2H), 5.16 (d, *J* = 4.80 Hz, 1H), 3.69 (s, 3H), 2.39 (s, 3H), 2.09 (s, 3H); ESI (*m/z*): 438 [$C_{23}H_{21}F_2N_5O_2$ + H]$^+$; HPLC (Method B) >99% (AUC), t_R = 8.15 min.

***N*-(4-(4-Amino-2,7-dimethyl-7*H*-pyrrolo[2,3-*d*]pyrimidin-5-yl)-3,5-dimethylphenyl)-2-(3-fluorophenyl)-2-hydroxyacetamide (68)**

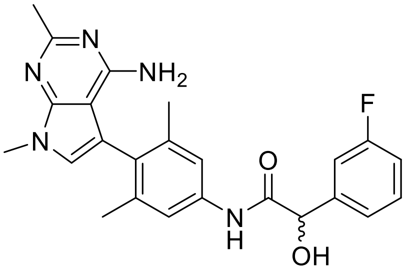

68

^{1}H NMR (400 MHz, DMSO-d6) δ 9.87 (s, 1H), 7.50 (s, 2H), 7.44–7.33 (m, 3H), 7.13 (t, *J* = 7.60 Hz, 1H), 6.92 (s, 1H), 6.57 (d, *J* = 4.80 Hz, 1H), 5.42 (brs, 2H), 5.15 (d, *J* = 4.80 Hz, 1H), 3.70 (s, 3H), 2.39 (s, 3H), 1.99 (s, 6H); ESI (m/z): 434 [$C_{24}H_{24}FN_5O_2$ + H]$^+$; HPLC (Method B) 98.6% (AUC), t_R = 8.20 min.

(*R*)-*N*-(4-(4-Amino-2,7-dimethyl-7*H*-pyrrolo[2,3-*d*]pyrimidin-5-yl)-2-fluorophenyl)-2-(3-fluorophenyl)-2-hydroxyacetamide (69)

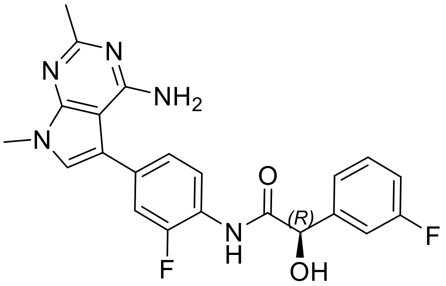

69

^{1}H NMR (400 MHz, DMSO-d_6) δ 9.69 (s, 1H), 7.91 (t, *J* = 8.40 Hz, 1H), 7.45–7.13 (m, 7H), 6.14 (brs, 1H), 6.07 (brs, 2H), 5.23 (s, 1H), 3.69 (s, 3H), 2.41 (s, 3H); ESI (m/z): 424 [$C_{22}H_{19}F_2N_5O_2$ + H]$^+$; HPLC (Method B) >99% (AUC), t_R = 8.00 min; Chiral SFC (Chiralcel OJ–H, Method C) >99% (AUC), t_R = 3.37 min.

***N*-(4-(4-Amino-2,7-dimethyl-7*H*-pyrrolo[2,3-*d*]pyrimidin-5-yl)-2,5-difluorophenyl)-2-(3-fluorophenyl)-2-hydroxyacetamide (71)**

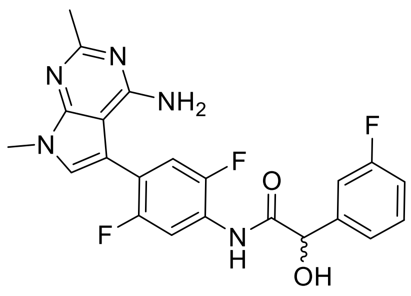

71

^{1}H NMR (400 MHz, DMSO-d_6) δ 7.94–7.89 (m, 1H), 7.44–7.24 (m, 5H), 7.16–7.12 (m, 1H), 6.06 (brs, 2H), 5.29 (s, 1H), 3.69 (s, 3H), 2.40 (s, 3H); ESI (m/z): 442 [$C_{22}H_{18}F_3N_5O_2$ + H]$^+$; HPLC (Method B) 98.9% (AUC), t_R = 8.23 min.

(*R*)-*N*-(5-(4-Amino-2,7-dimethyl-7*H*-pyrrolo[2,3-*d*]pyrimidin-5-yl)-6-methylpyridin-2-yl)-2-hydroxy-2-(3-(trifluoromethyl)phenyl)acetamide (73)

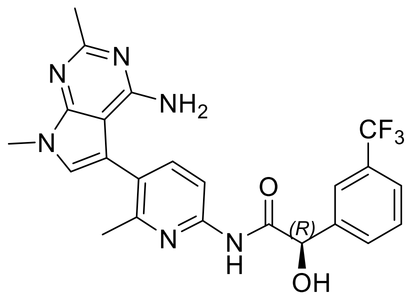

73

^{1}H NMR (400 MHz, DMSO-d_6) δ 10.22 (s, 1H), 7.93–7.91 (m, 2H), 7.85 (d, *J* = 7.6 Hz, 1H), 7.69–7.67 (m, 1H), 7.63–7.55 (m, 2H), 7.08 (s, 1H), 6.66 (d, *J* = 5.6 Hz, 1H), 5.81 (brs, 2H), 5.40 (d, *J* = 5.2 Hz, 1H), 3.68 (s, 3H), 2.39 (s, 3H), 2.34 (s, 3H); ESI (m/z): 471 [$C_{23}H_{21}F_3N_6O_2$ + H]$^+$; HPLC (Method B) 98.8% (AUC), t_R = 7.71 min; Chiral SFC (ChiralPak OD-H, Method E) >99% (AUC), t_R = 2.20 min.

(*R*)-*N*-(5-(4-Amino-2,7-dimethyl-7*H*-pyrrolo[2,3-*d*]pyrimidin-5-yl)-4-methylpyridin-2-yl)-2-(3-fluorophenyl)-2-hydroxyacetamide (74)

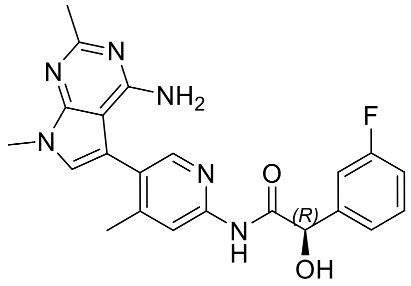

74

^{1}H-NMR (400 MHz, DMSO-d_6) δ: 10.04 (brs, 1H), 8.10 (s, 1H), 8.00 (s, 1H), 7.42–7.33 (m, 3H), 7.16–7.11 (m, 1H), 7.10 (s, 1H), 6.65 (brs, 1H), 5.82 (brs, 2H), 5.29 (s, 1H), 3.70 (s, 3H), 2.40 (s, 3H), 2.19 (s, 3H); ESI (m/z): 421 [$C_{22}H_{21}FN_6O_2$+H]$^+$; HPLC (Method B) 98.5% (AUC), t_R = 6.57 min; Chiral SFC (Chiralcel OX-H, Method B) 95.4% (AUC), t_R = 7.95 min.

(*R*)-*N*-(5-(4-Amino-2,7-dimethyl-7*H*-pyrrolo[2,3-*d*]pyrimidin-5-yl)-4-methylpyridin-2-yl)-2-hydroxy-2-(3-(trifluoromethyl)phenyl)acetamide (75)

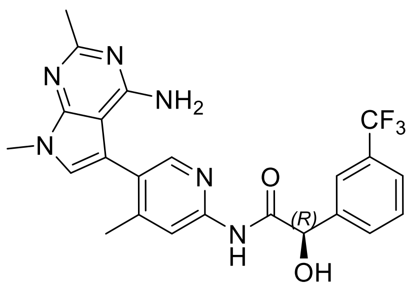

75

^{1}H NMR (400 MHz, DMSO-d_6) δ 10.13 (s, 1H), 8.11 (s, 1H), 8.01 (s, 1H), 7.95 (s, 1H), 7.85 (d, *J* =8.00 Hz, 1H), 7.68 (d, *J* =7.20 Hz, 1H), 7.61 (d, *J* =8.0 Hz, 1H), 7.10 (s, 1H), 6.74 (s, 1H), 5.81 (s, 2H), 5.40 (s, 1H), 3.70 (s, 3H), 2.40 (s, 3H), 2.19 (s, 3H); ESI (m/z): 471 [$C_{23}H_{21}F_3N_6O_2$ + H]$^+$, HPLC (Method B) 96.6% (AUC), t_R = 7.127 min; Chiral SFC (Chiralcel OX-H, Method B) 91.3% (AUC), t_R = 3.98 min.

(*R*)-*N*-(5-(4-Amino-2,7-dimethyl-7*H*-pyrrolo[2,3-*d*]pyrimidin-5-yl)-4-methylpyrimidin-2-yl)-2-(3-fluorophenyl)-2-hydroxyacetamide (76)

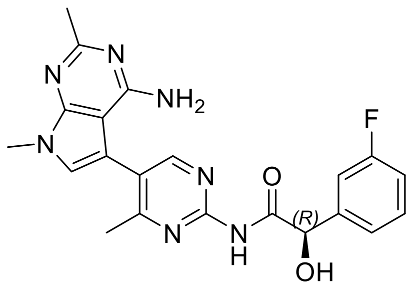

76

^{1}H NMR (400 MHz, DMSO-d_6) δ 10.26 (s, 1H), 8.31 (s, 1H), 7.44–7.33 (m, 3H), 7.13–7.10 (m, 2H), 6.50 (d, *J* = 5.60 Hz, 1H), 6.14 (brs, 2H), 5.31 (d, *J* = 5.20 Hz, 1H), 3.69 (s, 3H), 2.39 (s, 3H), 2.32 (s, 3H); ESI (m/z): 422 [$C_{21}H_{20}FN_7O_2$ + H]$^+$; HPLC (Method B) 97.4% (AUC), t_R = 6.40 min; Chiral SFC (Chiralcel OX-H, Method B) 84.3% (AUC), t_R = 6.19 min.

HPLC Conditions
Method A
Column: XBridge BEH C18 2.5µm(2.1*50mm)
Total Flow: 0.6mL/min
Mobile phase-A: 0.05%TFA in ACN
Mobile phase-B: 0.05%TFA in WATER
Column Temperature: Ambient
Gradient -%A: 0/10,1.5/10,5/70,7/95,8.5/95,8.6/10,10/10.
Diluent: Water: ACN (1:1) (%v /v)
Channel Description: PDA Spectrum

Method B
Column: Eclipse Plus C18, 100 x 4.6 mm,3.5µm.
Mobile Phase-A: 0.05%TFA in Water.
Mobile Phase-B: 0.05%TFA in Acetonitrile
Elution: Time/%B-0/5, 2/5, 7/70, 10/95, 12/95, 12.1/5, 15/5
Diluent: ACN: Water (1:1) (%v/v)
Flow: 1.0 mL/min;
Sample Temperature: Ambient
Column Temperature: Ambient
Detector: DAD

Method C:
Column: Polaris C18-A 100 x 3.0 mm, 2.6µm
Mobile Phase-A: 0.05% TFA in Water
Mobile Phase-B: 0.05%TFA in Acetonitrile

Elution: Time/%B-0/5, 3/5, 6/90, 12/90, 12.1/5, 15/5
Diluent: ACN **:** Water (1:1) % v/v
Flow: 0.8 mL/min;
Sampler Temperature: Ambient
Column Temperature: Ambient

SFC Conditions
Method A
Column: Chiralpak IA (4.6*150 mm)5µ
Total Flow: 3g-30%
Mobile phase (A%): CO_2 (2.1g/min)
Mobile phase (B%): 0.3 % DEA in MeOH (0.9mL/min)
Column Temperature: 40°C
Detection: 240 nm
ABPR: 1500 psi
Diluent: MeOH

Method B
Column: Chiralcel OX-H (4.6*150 mm)5µ
Total FLow: 3g-30%
Mobile phase (A%): CO_2 (2.1g/min)
Mobile phase (B%): 0.3 % DEA in MeOH (0.9mL/min)
Column Temperature: 40°C
Detection: 240 nm
ABPR: 1500 psi
Diluent: MeOH

Method C
Column: Chiralcel OJ-H (4.6*150 mm)5µ
Total FLow: 3g-15%
Mobile phase (A%): CO_2 (2.55g/min)
Mobile phase (B%): 0.3 % DEA in MeOH (0.45mL/min)
Column Temperature: 40°C
Detection: 240 nm
ABPR: 1500 psi
Diluent: MeOH

^{1}H NMR (400 MHz, DMSO-d_6)

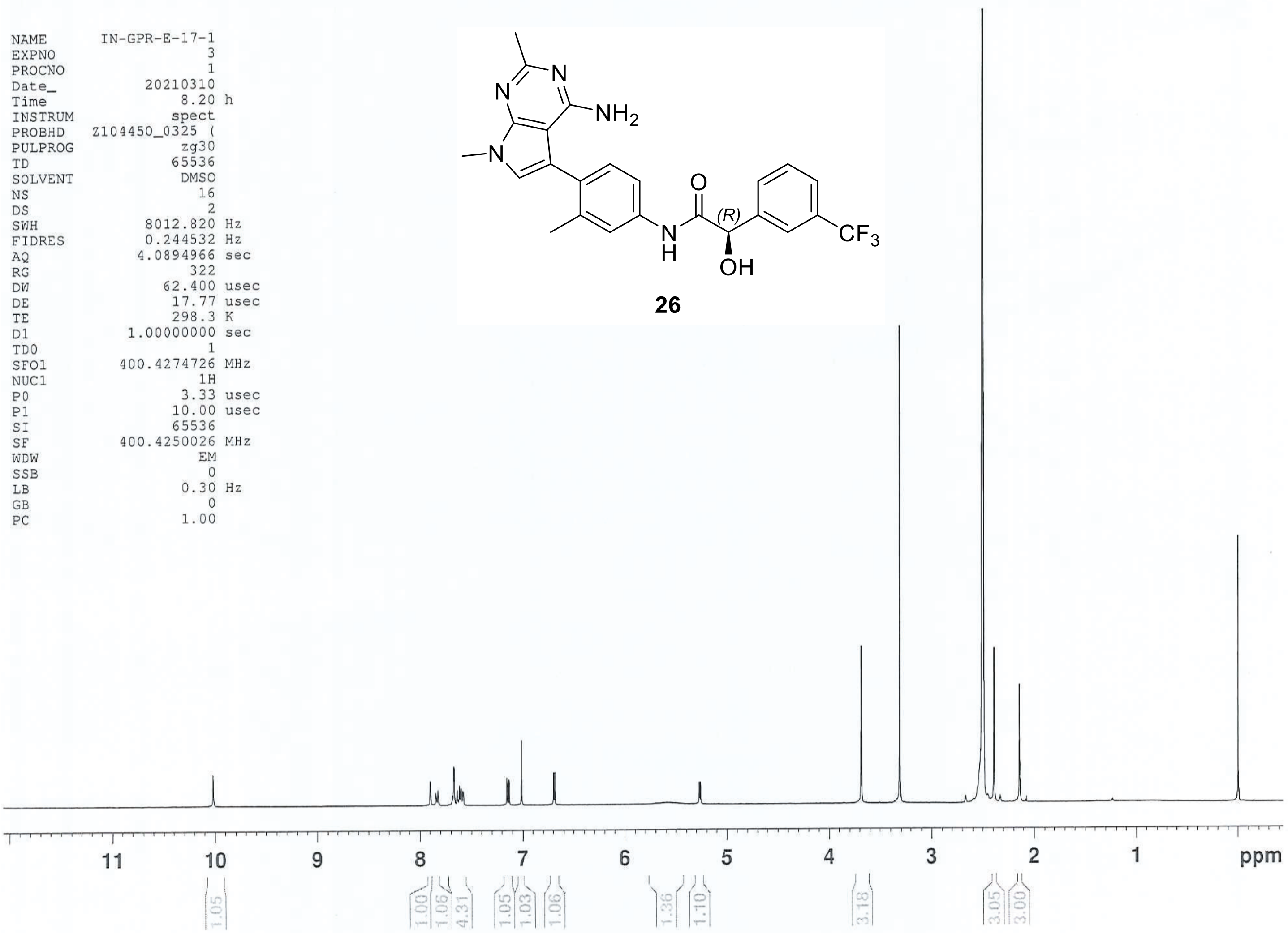

^1H NMR (400 MHz, DMSO-d_6)

```
NAME       IN-RRC-H-143-1
EXPNO                   4
PROCNO                  1
Date_            20191115
Time                 9.26
INSTRUM             spect
PROBHD   5 mm PABBO BB-
PULPROG              zg30
TD                  65536
SOLVENT              DMSO
NS                     16
DS                      2
SWH              8223.685 Hz
FIDRES           0.125483 Hz
AQ              3.9846387 sec
RG                    161
DW                 60.800 usec
DE                   6.50 usec
TE                  298.3 K
D1             1.00000000 sec
TD0                     1

======== CHANNEL f1 ========
NUC1                   1H
P1                  10.85 usec
PL1                 -2.00 dB
PL1W          14.33185768 W
SFO1          400.4274728 MHz
SI                  32768
SF            400.4250080 MHz
WDW                    EM
SSB                     0
LB                   0.30 Hz
GB                      0
PC                   1.00
```

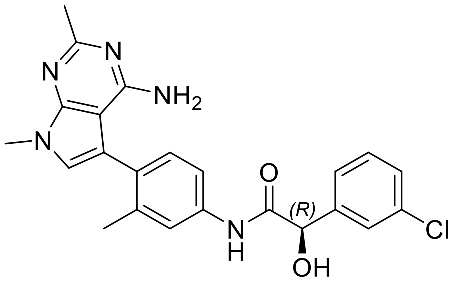

28

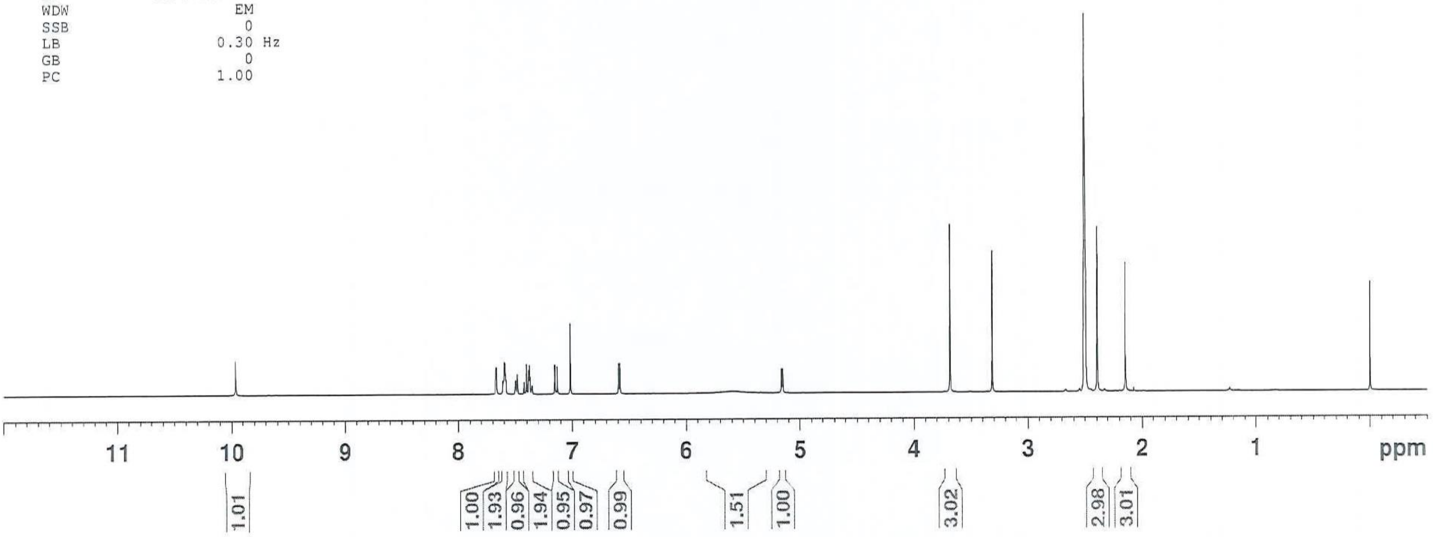

^{1}H NMR (400 MHz, DMSO-d_6)

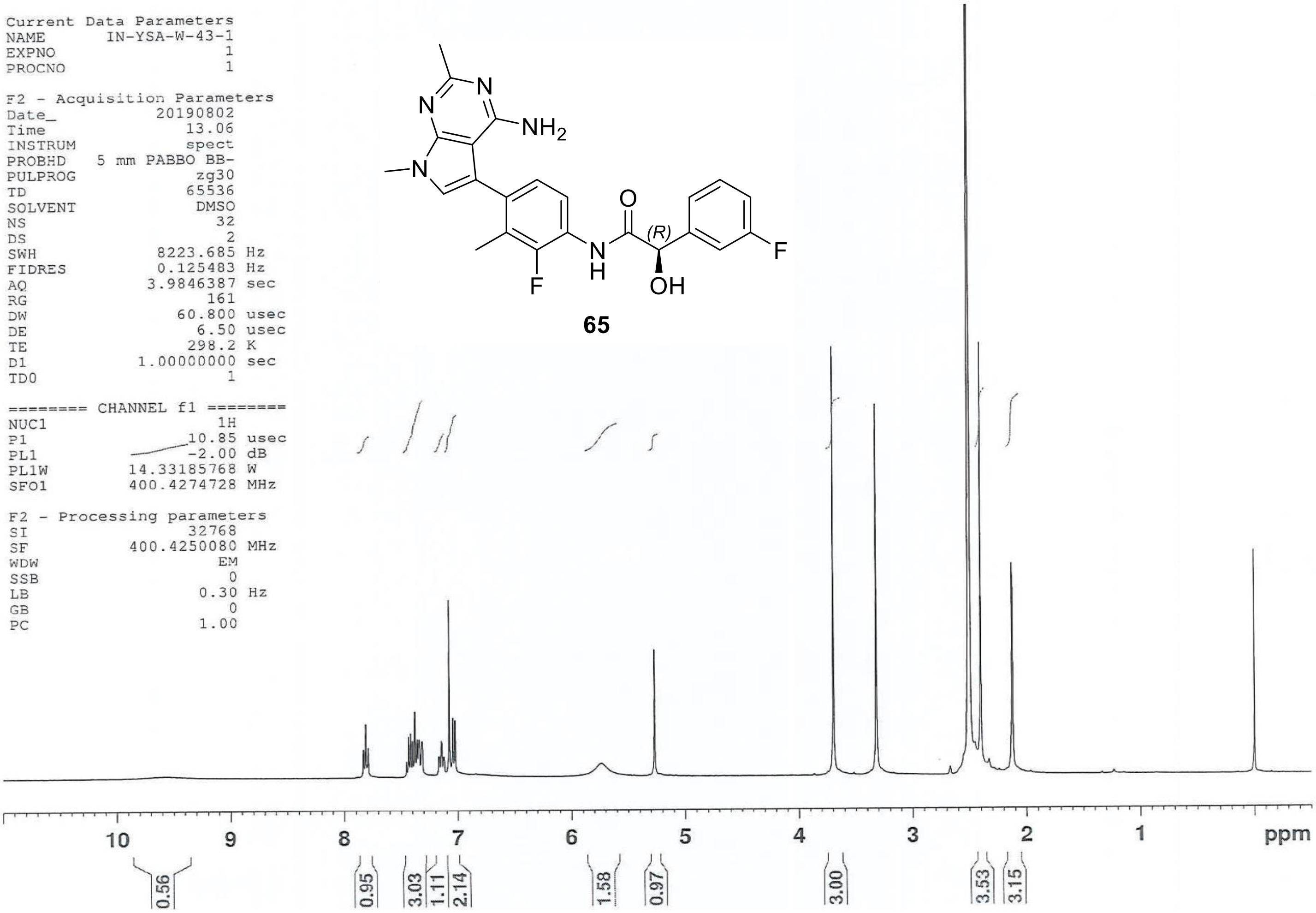

^{1}H NMR (400 MHz, DMSO-d_6)

NAME	IN-KNV-C-13-1
EXPNO	6
PROCNO	1
Date_	20190828
Time	10.59
INSTRUM	spect
PROBHD	5 mm PABBO BB-
PULPROG	zg30
TD	65536
SOLVENT	DMSO
NS	16
DS	2
SWH	8223.685 Hz
FIDRES	0.125483 Hz
AQ	3.9846387 sec
RG	161
DW	60.800 usec
DE	6.50 usec
TE	296.4 K
D1	1.00000000 sec
TD0	1

======== CHANNEL f1 ========

NUC1	1H
P1	10.85 usec
PL1	-2.00 dB
PL1W	14.33185768 W
SFO1	400.4274728 MHz
SI	32768
SF	400.4250079 MHz
WDW	EM
SSB	0
LB	0.30 Hz
GB	0
PC	1.00

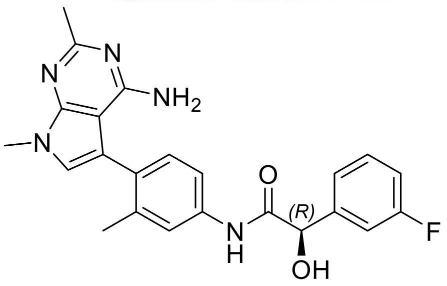

24

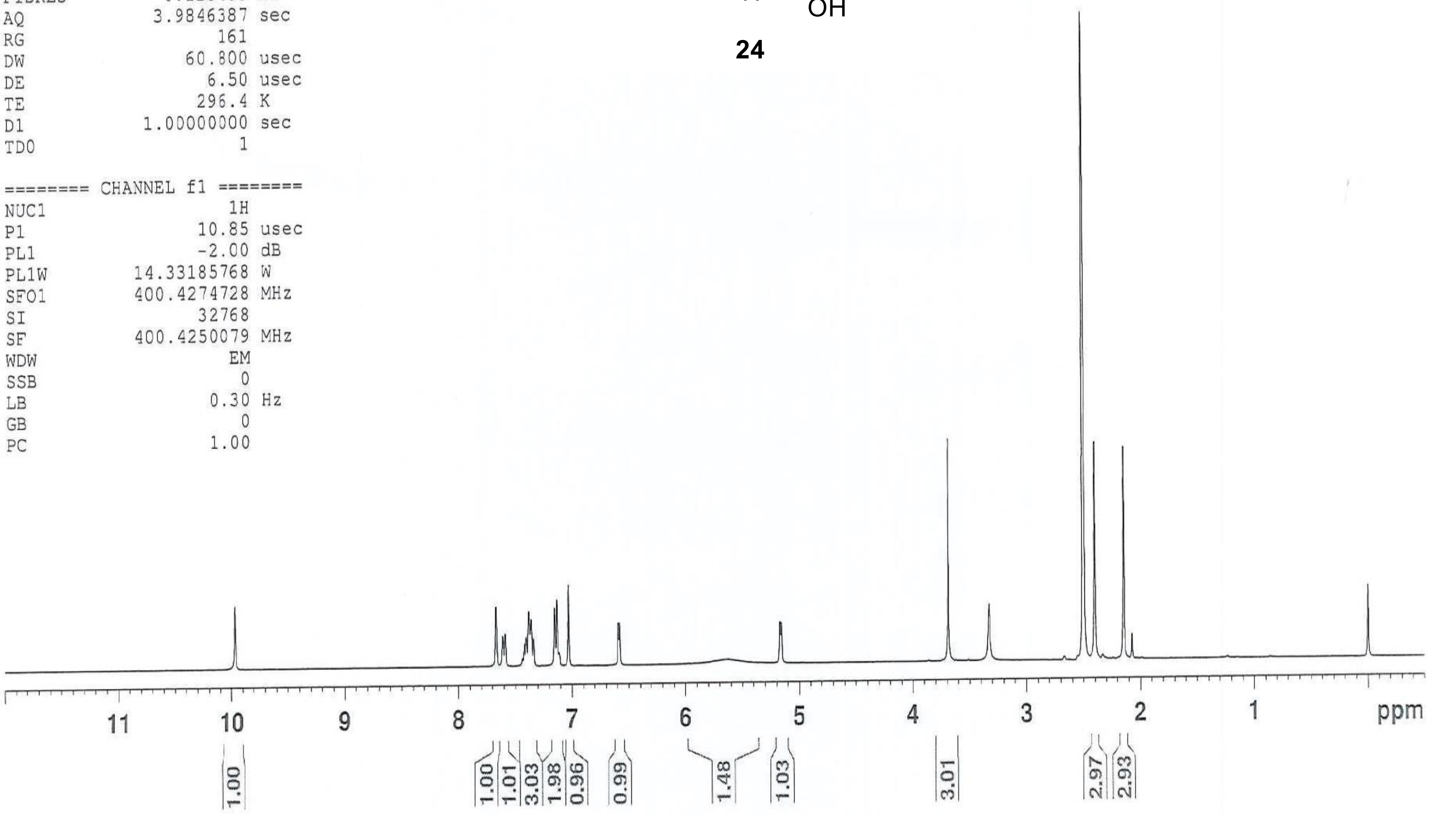

^{1}H NMR (400 MHz, DMSO-d_6)

```
NAME        IN-MVE-C-88-1
EXPNO                   1
PROCNO                  1
Date_            20191024
Time                10.05
INSTRUM             spect
PROBHD   5 mm PABBO BB-
PULPROG              zg30
TD                  65536
SOLVENT              DMSO
NS                     16
DS                      2
SWH              8223.685 Hz
FIDRES           0.125483 Hz
AQ              3.9846387 sec
RG                    144
DW                 60.800 usec
DE                   6.50 usec
TE                  298.2 K
D1             1.00000000 sec
TD0                     1

======== CHANNEL f1 ========
NUC1                   1H
P1                  10.85 usec
PL1                 -2.00 dB
PL1W          14.33185768 W
SFO1          400.4274728 MHz
SI                  32768
SF            400.4250077 MHz
WDW                    EM
SSB                     0
LB                   0.30 Hz
GB                      0
PC                   1.00
```

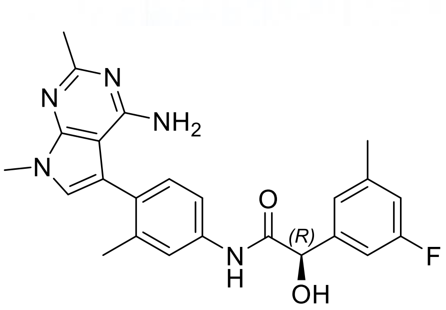

41

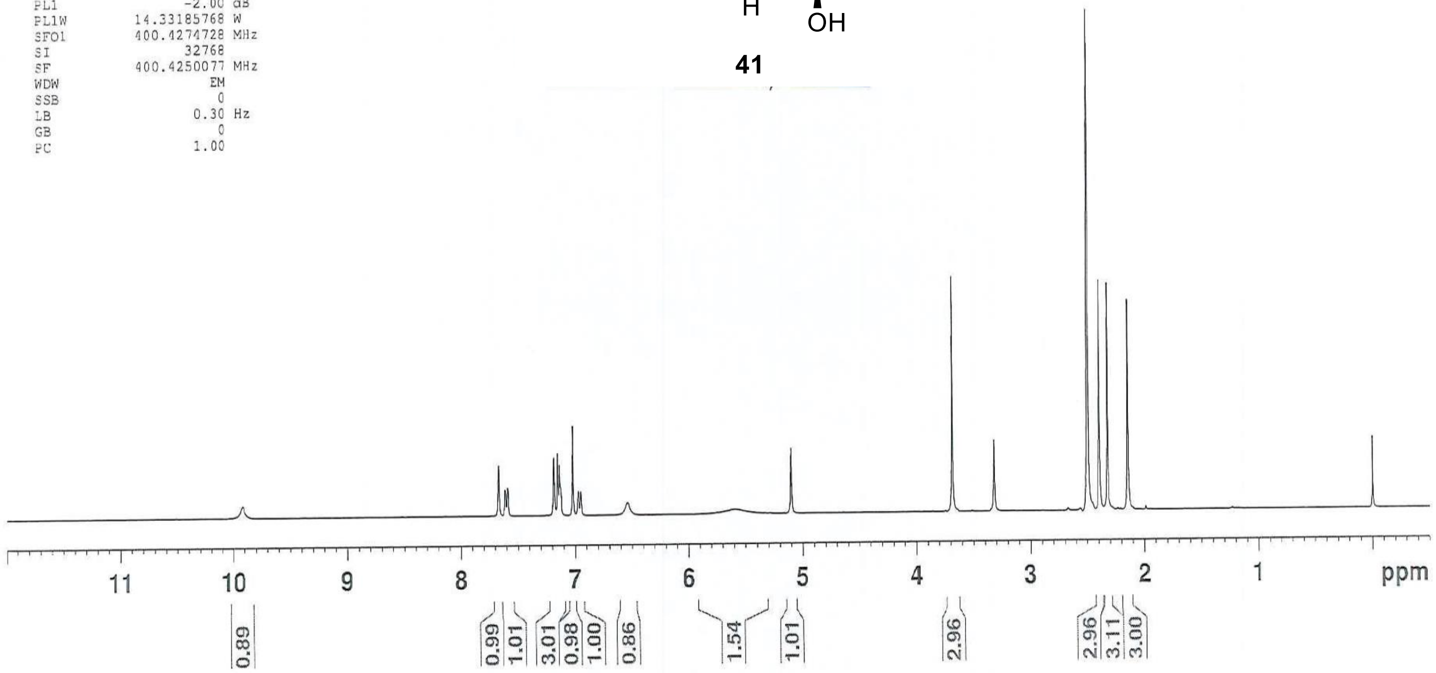

^1H NMR (400 MHz, DMSO-d_6)

```
NAME       IN-MVE-C-87-1
EXPNO                  1
PROCNO                 1
Date_           20191023
Time               10.20
PROBHD   5 mm QNP 1H/13
SOLVENT             DMSO
NS                    16
TE                 294.6 K
DS                     2
PULPROG             zg30
INSTRUM            spect
TD                 65536
D1            1.00000000 sec
TD0                    1

======== CHANNEL f1 ========
NUC1                  1H
P1                 14.00 usec
PL1                -1.40 dB
PL1W        13.31469536 W
SFO1        400.1324710 MHz
SI                 32768
SF          400.1300022 MHz
WDW                   EM
SSB                    0
LB                  0.30 Hz
GB                     0
PC                  1.00
```

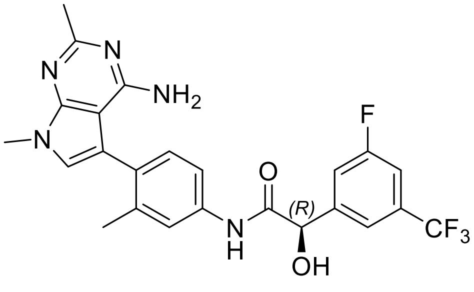

39

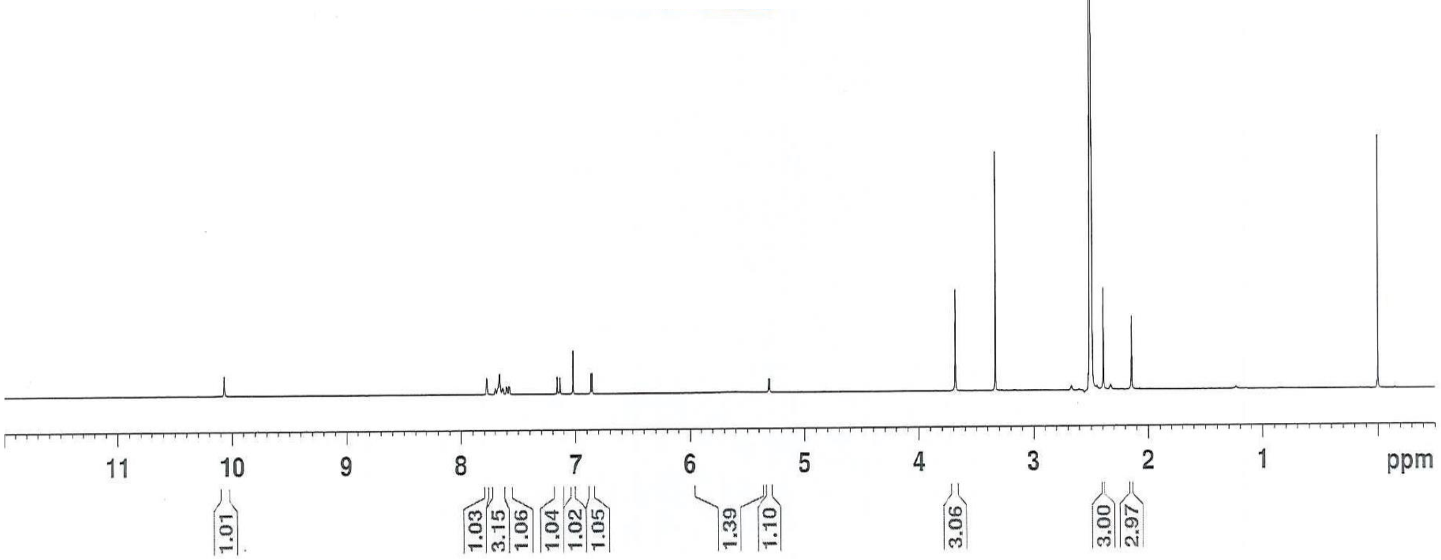

^{1}H NMR (400 MHz, DMSO-d_6)

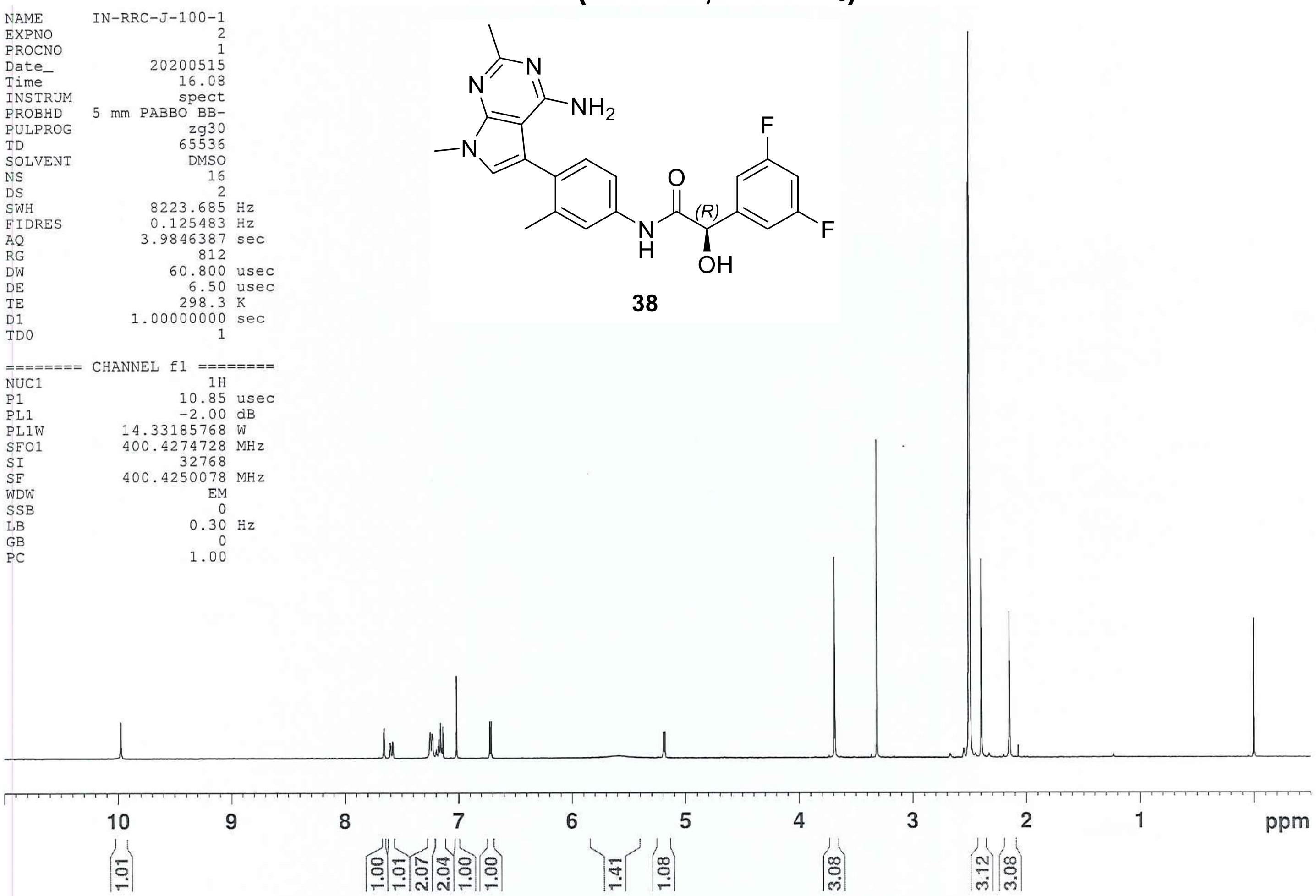

^{1}H NMR (400 MHz, DMSO-d_6)

```
NAME       IN-BNA-B-155-2
EXPNO                   1
PROCNO                  1
Date_            20191106
Time                10.28
INSTRUM             spect
PROBHD   5 mm PABBO BB-
PULPROG              zg30
TD                  65536
SOLVENT              DMSO
NS                     16
DS                      2
SWH            8223.685 Hz
FIDRES         0.125483 Hz
AQ            3.9846387 sec
RG                    256
DW               60.800 usec
DE                 6.50 usec
TE                298.2 K
D1           1.00000000 sec
TD0                     1

======== CHANNEL f1 ========
NUC1                   1H
P1                10.85 usec
PL1               -2.00 dB
PL1W        14.33185768 W
SFO1        400.4274728 MHz
SI                  32768
SF          400.4250082 MHz
WDW                    EM
SSB                     0
LB                 0.30 Hz
GB                      0
PC                   1.00
```

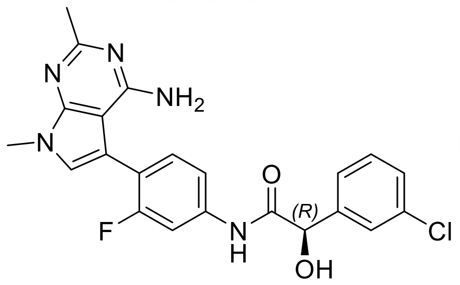

48

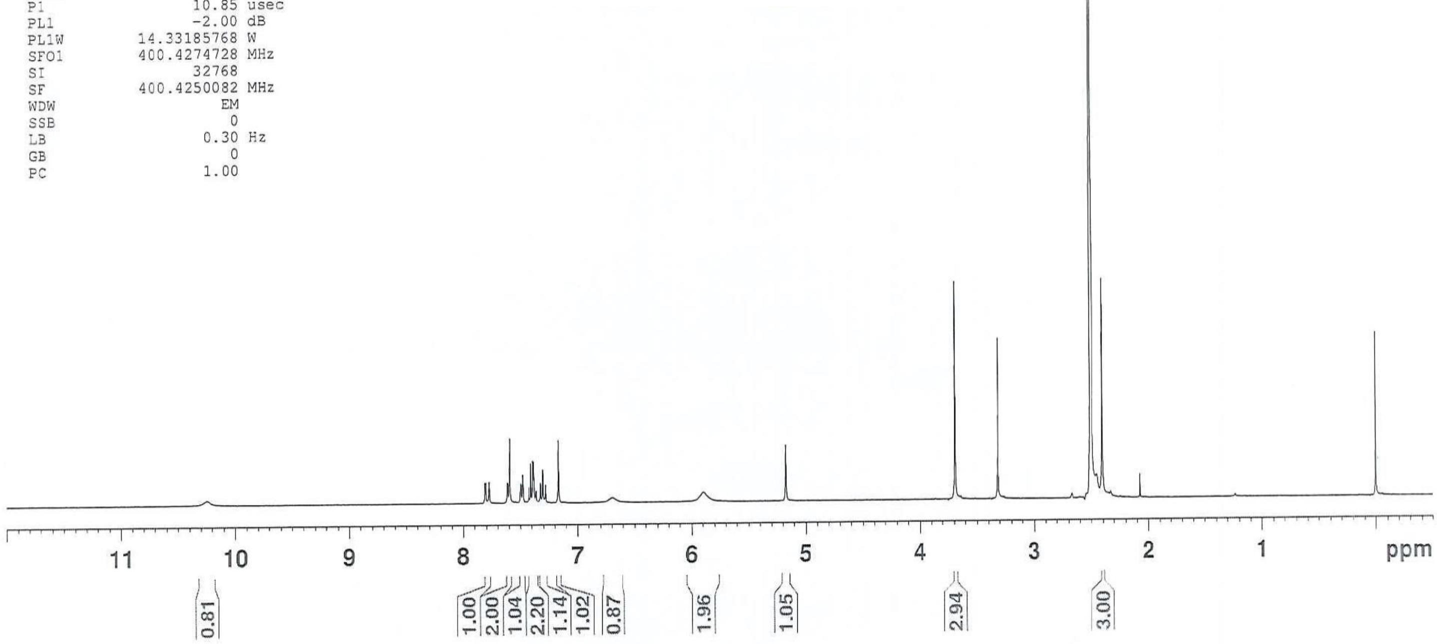

^1H NMR (400 MHz, DMSO-d_6)

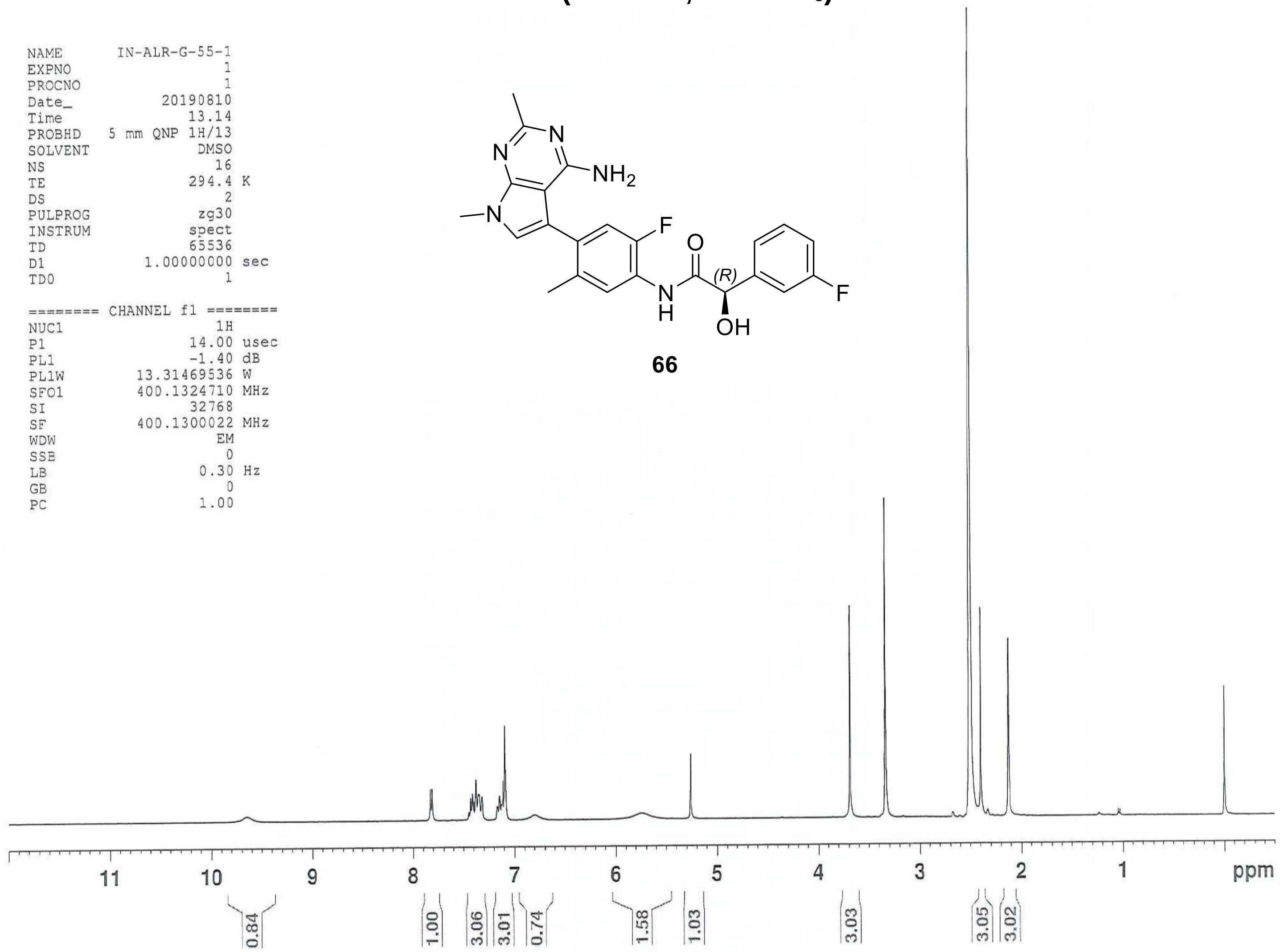

^{1}H NMR (400 MHz, DMSO-d_6)

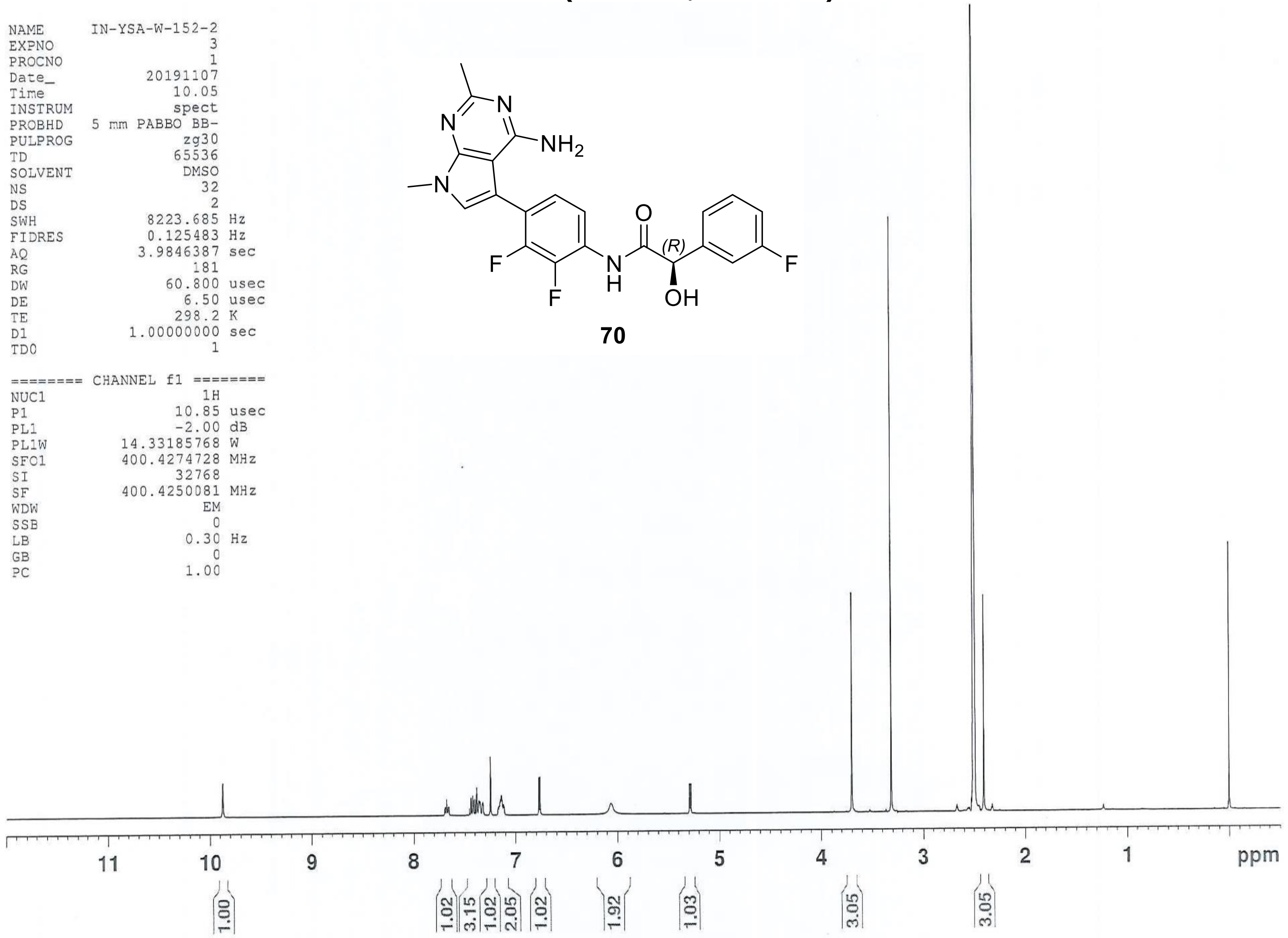

70

2. Crystallography

Supplemental Table S1. Crystallography data collection and refinement statistics.

Inhibitor	11	24	26
PDB ID	8EQ9	8EQD	8EQE
Data collection beamline	APS 17ID	APS 23-ID-D	APS 23-ID-D
Wavelength (Å)	1.00	1.033	1.033
Space group	$P3_221$	$P3_221$	$P3_221$
Unit cell			
a = b (Å)	126.73	125.90	125.34
c (Å)	58.77	55.22	58.08
α=β (°)	90	90	90
γ (°)	120	120	120
Resolution (Å)	55 – 2.86	50 – 2.92	55.0 – 2.56
Highest-resolution shell (Å)	2.93 – 2.86	3.00 – 2.92	2.63 – 2.56
No. of total reflections	122456	71294	167478
No. of unique reflections	12802	10640	17214
R_{merge} (%)	12.6 (69.6)	9.7 (85.3)	7.2 (118.0)*
I/σ(I)	8.0 (2.4)	9.5 (1.7)	15.0 (1.8)
Completeness (%)	100.0 (100.0)	96.2 (98.8)	100.0 (100.0)
Multiplicity	9.6 (10.0)	6.7 (6.8)	9.7 (10.0)
Refinement			
R_{work}	0.239 (0.453)	0.246 (0.481)	0.224 (0.477)
R_{free}	0.277 (0.469)	0.280 (0.428)	0.287 (0.480)
No. of protein atoms	2095	2106	2108
No. of inhibitor atoms	30	31	34
No. of water atoms	24	0	6
Average B-factor, protein ($Å^2$)	84.2	101.1	84.5
Average B-factor, inhibitor ($Å^2$)	47.8	63.2	56.5
Average B-factor, water ($Å^2$)	49.6	-	56.5
r.m.s.d. bond length (Å)	0.004	0.002	0.003
r.m.s.d. angle (°)	1.30	1.21	1.25
Ramachandran plot#			
Favored (%)	91.8	94.8	95.2
Allowed (%)	8.2	5.2	4.8
Outliers (%)	0.0	0.0	0.0

* The values in parenthesis are for the highest-resolution shell.
# Calculated by Molprobity (1).

3. Supplemental Data

Cellular viability assays using AML and CEL cell lines, **26** exhibited IC_{50} values of 0.226 µM and 0.311 µM in the FLT3-ITD mutant cell lines MV4-11 and MOLM-13, and no inhibitory effect up to 10 µM against two FLT3wt lines MOLM-16 and Kasumi. The inhibitory effect of **26** against FLT3-ITD was evaluated in cells by quantifying p-STAT5 and p-ERK, two canonical downstream readouts of FLT3-ITD activity in MV4-11 cells. Treatment with **26** inhibited pSTAT5 and pERK at IC_{50} values of 64 nM and 551 nM, respectively. Together, these data indicate **26** presents moderate inhibitory activity against FLT3-ITD mutant kinase but not wild-type FLT3 (in contrast, other FLT3 inhibitors- gilteritinib, crenolanib, quizartinib- decrease viability of FLT3wt cell lines; Supplemental Figure S1).

	Viability IC_{50} (µM)			
Compound	MV4-11 (FLT3-ITD)	MOLM-13 (FLT3-ITD)	MOLM-16 (FLT3wt)	Kasumi-1 (FLT3wt)
**26**	0.226	0.311	>10	>10
Gilteritinib	n.d.	0.014	n.d.	0.478
Crenolanib	n.d.	0.003	n.d.	0.08
Quizartinib	n.d.	<0.001	n.d.	0.006

A)

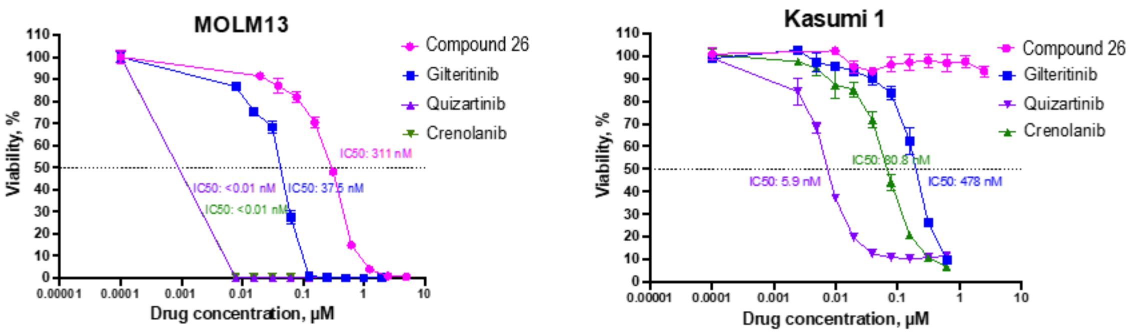

B)

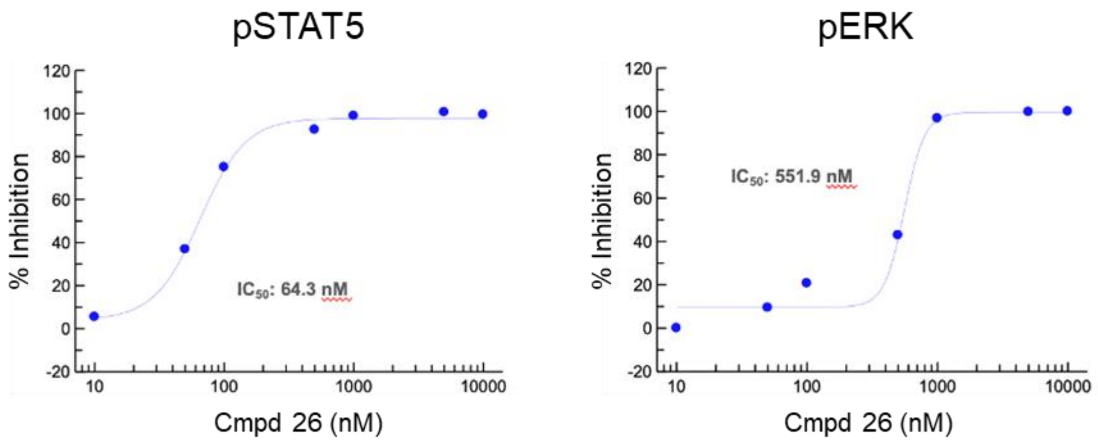

Supplemental Figure S1. Cmpd 26 is selective against cell lines driven by FLT3-ITD. A) in vitro cell viability IC_{50} in human AML and CEL cancer cell lines expressing either FLT3wt or FLT3-ITD mutation background. Viability IC_{50} graphs for **26** and FLT3

inhibitors (Gilteritinib, Quizartinib, Crenolab). MV4-11 and MOLM-16 were not tested with FLT3 inhibitors (not determined; n.d). B) IC_{50} values for inhibition of FLT3-ITD downstream signaling through STAT5 and ERK in MV4-11 cells. Values were normalized to Crenolanib (100% inhibition).

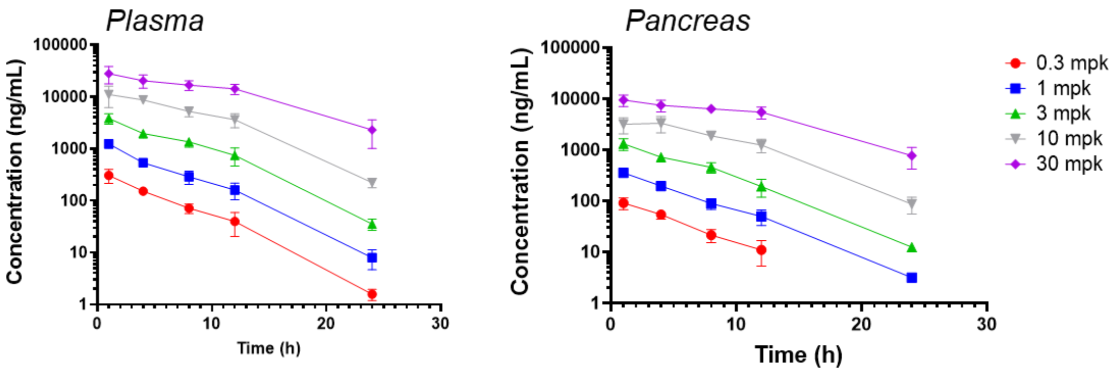

Supplemental Figure S2. In vivo PK in plasma and pancreas. Plasma and pancreas sampled at 1, 4, 8, 12, 24 h following a single oral administration of **26** at indicated dose levels. Values indicate average of 5 mice; **26** abundance determined by LC-MS/MS.

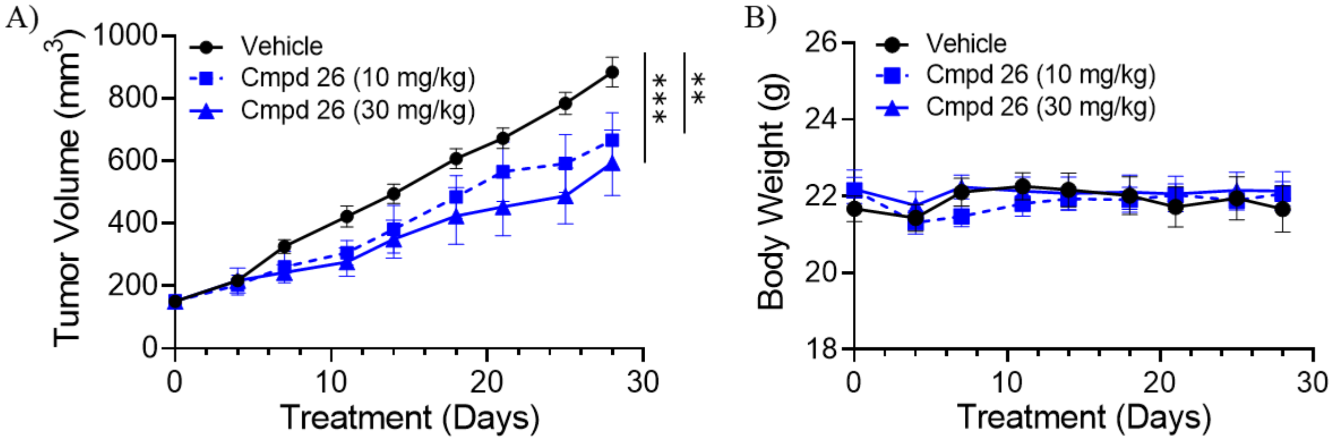

Supplemental Figure S3. Compound 26 slows growth of 786-O RCC tumor xenografts. A) Mice harboring 786-O xenografts were treated with 10 or 30 mg/kg 26 for 28 days. Treatments significantly decreased tumor volume (ANOVA; ** $p<0.01$, *** $p<0.001$). B) Mouse body weight measurements taken across the 28-day study period. No significant effects on body weght were observed.

Supplemental Table S2. scanMAXSM Kinome Assay Results. Complete tabulated data of all 468 kinases from the panel, including 403 wild-type and 65 mutant isoforms. Values displayed represent Percent Activity (%) relative to control. Cmpd **11** and Cmpd **24** assayed at 1000 nM; Cmpd **26** assayed at both 100 nM and 1000 nM.

	% Control			
DiscoveRx Gene Symbol	**11** (1000 nM)	**24** (1000 nM)	**26** (100 nM)	**26** (1000 nM)
AAK1	89	99	100	96
ABL1(E255K)-phosph.	65	100	100	89
ABL1(F317I)-nonphosph.	96	100	100	99
ABL1(F317I)-phosph.	92	77	100	96
ABL1(F317L)-nonphosph.	86	100	100	96
ABL1(F317L)-phosph.	86	100	100	93
ABL1(H396P)-nonphosph.	71	95	100	91
ABL1(H396P)-phosph.	75	76	100	100
ABL1(M351T)-phosph.	98	100	100	93
ABL1(Q252H)-nonphosph.	66	98	99	78
ABL1(Q252H)-phosph.	98	100	100	74
ABL1(T315I)-nonphosph.	72	100	100	89
ABL1(T315I)-phosph.	75	100	100	91
ABL1(Y253F)-phosph.	82	63	100	77
ABL1-nonphosph.	55	100	98	84
ABL1-phosph.	68	100	97	72
ABL2	100	100	100	97
ACVR1	99	100	100	100
ACVR1B	86	95	100	95
ACVR2A	100	100	75	100
ACVR2B	100	100	100	100
ACVRL1	100	100	100	100
ADCK3	93	100	100	100
ADCK4	100	91	100	100
AKT1	90	100	86	100
AKT2	100	100	100	100
AKT3	88	100	100	100
ALK	90	100	96	36
ALK(C1156Y)	78	100	100	57
ALK(L1196M)	85	100	100	93
AMPK-alpha1	94	100	98	45
AMPK-alpha2	76	85	77	96
ANKK1	78	96	100	85
ARK5	100	96	100	93
ASK1	92	95	100	99
ASK2	93	86	95	100
AURKA	72	100	98	96
AURKB	24	90	81	70
AURKC	81	100	100	100
AXL	56	100	99	0.7
BIKE	100	97	100	94
BLK	19	100	100	91
BMPR1A	86	96	100	95
BMPR1B	99	100	97	80
BMPR2	96	100	100	99
BMX	87	100	100	84
BRAF	76	100	100	96
BRAF(V600E)	52	100	99	99
BRK	94	100	100	97
BRSK1	100	78	100	87
BRSK2	78	86	100	97
BTK	95	92	100	73
BUB1	100	97	81	97
CAMK1	44	100	100	96
CAMK1B	98	91	100	100
CAMK1D	70	100	100	100
CAMK1G	77	100	99	96
CAMK2A	93	100	100	93
CAMK2B	100	100	100	99
CAMK2D	80	65	97	88
CAMK2G	66	100	100	91
CAMK4	32	100	100	75
CAMKK1	100	90	100	84
CAMKK2	100	90	97	84
CASK	95	100	89	88
CDC2L1	100	97	100	100
CDC2L2	90	99	100	100
CDC2L5	100	82	100	89
CDK11	1	89	89	8.2
CDK2	100	100	94	98
CDK3	85	100	100	97
CDK4	100	100	100	85
CDK4-cyclinD1	100	100	100	93
CDK4-cyclinD3	94	100	100	84
CDK5	95	100	95	100
CDK7	38	98	100	70
CDK8	2.5	98	100	57
CDK9	100	100	100	100
CDKL1	73	98	96	90
CDKL2	97	100	100	100
CDKL3	78	100	93	99
CDKL5	92	86	100	90
CHEK1	100	89	100	98
CHEK2	90	97	94	94
CIT	93	99	100	97
CLK1	86	100	100	77
CLK2	95	100	100	91
CLK3	81	100	100	76
CLK4	93	100	87	100
CSF1R	45	94	99	15
CSF1R-autoinhibited	53	100	100	89
CSK	83	99	100	100
CSNK1A1	62	100	100	97
CSNK1A1L	99	100	100	100
CSNK1D	100	100	100	91
CSNK1E	63	100	100	89
CSNK1G1	95	100	100	81
CSNK1G2	96	100	98	99
CSNK1G3	97	100	100	99
CSNK2A1	93	95	84	90
CSNK2A2	68	100	100	88
CTK	60	100	100	100
DAPK1	100	100	100	100
DAPK2	100	100	100	99
DAPK3	100	99	100	94
DCAMKL1	44	82	100	100
DCAMKL2	93	98	100	100
DCAMKL3	69	100	100	100
DDR1	72	100	100	29
DDR2	100	96	100	68
DLK	100	97	100	84
DMPK	100	91	100	100
DMPK2	74	100	100	100
DRAK1	90	97	100	98
DRAK2	98	95	99	73
DYRK1A	77	86	100	77
DYRK1B	47	100	93	96
DYRK2	89	90	90	100
EGFR	100	81	100	85
EGFR(E746-A750del)	63	73	100	97
EGFR(G719C)	67	88	100	83

EGFR(G719S)	84	91	100	100
EGFR(L747-E749del, A750P)	84	100	93	88
EGFR(L747-S752del, P753S)	100	99	100	94
EGFR(L747-T751del,Sins)	95	100	96	100
EGFR(L858R)	100	81	99	86
EGFR(L858R,T790M)	83	98	78	100
EGFR(L861Q)	94	100	96	100
EGFR(S752-I759del)	100	100	94	100
EGFR(T790M)	94	89	100	100
EIF2AK1	49	73	89	78
EPHA1	55	100	100	99
EPHA2	87	100	100	100
EPHA3	100	100	100	99
EPHA4	78	98	100	88
EPHA5	100	100	99	100
EPHA6	100	86	98	79
EPHA7	97	100	100	100
EPHA8	100	100	100	93
EPHB1	100	100	100	97
EPHB2	76	100	94	95
EPHB3	99	100	100	96
EPHB4	100	100	100	94
EPHB6	2.6	100	94	48
ERBB2	100	75	100	100
ERBB3	89	76	100	98
ERBB4	85	95	100	97
ERK1	98	100	96	99
ERK2	98	73	99	100
ERK3	100	100	100	97
ERK4	100	100	100	100
ERK5	64	82	100	75
ERK8	89	100	100	92
ERN1	88	100	82	95
FAK	90	100	100	100
FER	87	100	100	98
FES	100	96	100	100
FGFR1	91	95	100	100
FGFR2	92	100	100	100
FGFR3	100	100	100	100
FGFR3(G697C)	93	85	92	100
FGFR4	87	95	98	79
FGR	89	97	100	96
FLT1	81	99	100	90
FLT3	4.3	66	41	0
FLT3(D835H)	33	98	85	25
FLT3(D835V)	52	100	100	8.5
FLT3(D835Y)	80	96	88	35
FLT3(ITD)	34	94	95	9.4
FLT3(ITD,D835V)	89	100	100	75
FLT3(ITD,F691L)	100	63	41	42
FLT3(K663Q)	26	83	51	20
FLT3(N841I)	0.3	100	34	0
FLT3(R834Q)	59	98	84	7.5
FLT3-autoinhibited	57	100	100	65
FLT4	100	100	100	78
FRK	95	100	96	90
FYN	78	100	97	98
GAK	79	100	100	91
GCN2(Kin.Dom.2,S808G)	84	100	76	94
GRK1	65	89	99	79
GRK2	100	100	100	87
GRK3	97	100	98	82
GRK4	100	100	100	100
GRK7	100	100	100	99
GSK3A	66	83	100	98
GSK3B	100	100	97	86
HASPIN	100	37	75	81
HCK	85	100	94	67
HIPK1	66	100	100	77
HIPK2	77	96	100	100

HIPK3	97	100	100	69
HIPK4	16	100	69	3.8
HPK1	53	90	83	97
HUNK	91	100	100	100
ICK	93	69	92	80
IGF1R	90	100	94	100
IKK-alpha	81	98	96	100
IKK-beta	69	100	92	99
IKK-epsilon	67	90	97	72
INSR	77	100	99	71
INSRR	100	88	100	95
IRAK1	86	92	100	100
IRAK3	82	90	100	60
IRAK4	68	95	100	94
ITK	100	100	100	97
JAK1(JH1domain-catalytic)	100	100	100	96
JAK1(JH2domain-pseudok)	54	54	100	92
JAK2(JH1domain-catalytic)	74	100	99	85
JAK3(JH1domain-catalytic)	91	84	100	88
JNK1	95	86	91	89
JNK2	100	88	95	70
JNK3	93	100	91	69
KIT	38	100	97	0.65
KIT(A829P)	89	100	100	100
KIT(D816H)	100	100	85	69
KIT(D816V)	96	100	100	100
KIT(L576P)	36	98	85	2.6
KIT(V559D)	27	72	96	0.45
KIT(V559D,T670I)	100	98	93	69
KIT(V559D,V654A)	100	94	98	62
KIT-autoinhibited	85	88	100	100
LATS1	94	100	98	82
LATS2	86	78	89	86
LCK	55	93	97	92
LIMK1	86	100	100	100
LIMK2	100	100	94	66
LKB1	100	95	100	99
LOK	4	88	94	12
LRRK2	71	100	100	87
LRRK2(G2019S)	94	100	97	81
LTK	35	100	98	22
LYN	85	83	99	99
LZK	100	100	98	90
MAK	99	100	100	100
MAP3K1	61	100	88	82
MAP3K15	29	82	100	99
MAP3K2	14	100	100	89
MAP3K3	32	100	100	92
MAP3K4	70	100	92	82
MAP4K2	100	94	94	89
MAP4K3	53	98	98	74
MAP4K4	84	100	100	98
MAP4K5	68	100	100	96
MAPKAPK2	100	93	100	100
MAPKAPK5	100	100	99	88
MARK1	85	97	98	98
MARK2	100	100	100	100
MARK3	100	76	99	96
MARK4	67	100	100	100
MAST1	81	100	100	89
MEK1	90	92	97	96
MEK2	75	69	99	98
MEK3	83	88	100	98
MEK4	75	81	100	100
MEK5	1.1	100	100	68
MEK6	55	100	87	100
MELK	91	93	97	100
MERTK	65	100	94	29
MET	66	100	96	90
MET(M1250T)	85	100	100	90

MET(Y1235D)	67	100	100	82
MINK	50	100	100	100
MKK7	91	100	99	100
MKNK1	53	100	79	81
MKNK2	1.2	58	88	3.9
MLCK	67	63	100	94
MLK1	100	100	96	97
MLK2	62	78	100	96
MLK3	91	95	99	95
MRCKA	98	100	100	100
MRCKB	95	100	100	100
MST1	82	100	100	100
MST1R	83	61	100	100
MST2	65	100	100	100
MST3	100	100	100	96
MST4	88	95	94	100
MTOR	52	100	86	89
MUSK	100	88	100	88
MYLK	59	100	100	91
MYLK2	71	100	100	100
MYLK4	64	92	92	100
MYO3A	83	100	100	79
MYO3B	47	100	100	65
NDR1	80	90	100	100
NDR2	96	99	100	89
NEK1	2.9	100	88	100
NEK10	100	100	100	95
NEK11	100	100	99	76
NEK2	81	81	100	85
NEK3	100	65	100	83
NEK4	93	99	100	100
NEK5	34	100	100	96
NEK6	87	97	94	100
NEK7	95	94	100	100
NEK9	67	100	97	98
NIK	94	100	92	77
NIM1	98	95	93	90
NLK	100	100	100	98
OSR1	94	100	96	88
p38-alpha	97	100	100	87
p38-beta	92	100	100	91
p38-delta	100	95	100	98
p38-gamma	98	100	85	92
PAK1	87	100	100	100
PAK2	97	100	100	100
PAK3	36	100	97	31
PAK4	95	100	100	100
PAK6	100	100	100	100
PAK7	96	94	100	90
PCTK1	90	95	98	80
PCTK2	100	99	100	100
PCTK3	73	100	100	100
PDGFRA	70	94	100	39
PDGFRB	40	100	100	11
PDPK1	79	100	76	89
PFCDPK1(P.falciparum)	83	89	100	76
PFPK5(P.falciparum)	100	92	90	98
PFTAIRE2	94	100	100	100
PFTK1	59	77	100	100
PHKG1	68	100	100	91
PHKG2	82	100	100	95
PIK3C2B	27	100	100	78
PIK3C2G	100	100	62	58
PIK3CA	100	100	100	76
PIK3CA(C420R)	100	100	100	89
PIK3CA(E542K)	89	0.7	100	91
PIK3CA(E545A)	96	100	100	98
PIK3CA(E545K)	51	85	99	82
PIK3CA(H1047L)	91	100	94	100
PIK3CA(H1047Y)	100	100	70	55

PIK3CA(I800L)	94	74	98	91
PIK3CA(M1043I)	100	100	82	86
PIK3CA(Q546K)	100	72	100	87
PIK3CB	100	100	100	95
PIK3CD	65	100	89	76
PIK3CG	63	100	100	89
PIK4CB	84	28	93	4.3
PIKFYVE	100	63	85	88
PIM1	93	73	98	100
PIM2	94	100	88	100
PIM3	98	99	99	100
PIP5K1A	94	90	80	100
PIP5K1C	99	34	100	91
PIP5K2B	94	100	95	73
PIP5K2C	79	81	95	100
PKAC-alpha	95	100	81	93
PKAC-beta	82	100	100	100
PKMYT1	100	100	100	89
PKN1	100	100	100	96
PKN2	78	100	97	95
PKNB(M.tuberculosis)	97	100	100	94
PLK1	78	100	94	98
PLK2	92	89	100	95
PLK3	94	78	100	100
PLK4	87	95	100	97
PRKCD	76	87	100	100
PRKCE	91	65	98	97
PRKCH	90	100	84	100
PRKCI	45	100	90	91
PRKCQ	82	81	100	93
PRKD1	89	100	98	98
PRKD2	95	100	99	99
PRKD3	100	100	100	98
PRKG1	93	100	100	100
PRKG2	98	100	100	95
PRKR	42	42	91	96
PRKX	100	100	100	87
PRP4	100	100	80	100
PYK2	86	100	100	100
QSK	82	100	83	81
RAF1	83	90	87	74
RET	99	100	100	92
RET(M918T)	87	99	99	83
RET(V804L)	90	100	99	93
RET(V804M)	100	98	100	99
RIOK1	54	100	100	100
RIOK2	20	89	91	92
RIOK3	100	100	100	100
RIPK1	100	85	99	97
RIPK2	15	100	91	43
RIPK4	93	80	100	99
RIPK5	3.4	100	100	100
ROCK1	100	91	90	71
ROCK2	100	100	98	87
ROS1	79	100	100	95
RPS6KA4(Kin.Dom.1-N-term.)	50	100	100	92
RPS6KA4(Kin.Dom.2-C-term.)	100	100	90	90
RPS6KA5(Kin.Dom.1-N-term.)	77	82	87	100
RPS6KA5(Kin.Dom.2-C-term.)	100	100	100	95
RSK1(Kin.Dom.1-N-term.)	97	100	97	100
RSK1(Kin.Dom.2-C-term.)	100	100	100	100
RSK2(Kin.Dom.1-N-term.)	58	90	77	66
RSK2(Kin.Dom.2-C-term.)	98	96	88	79
RSK3(Kin.Dom.1-N-term.)	100	100	100	97
RSK3(Kin.Dom.2-C-term.)	100	93	100	98
RSK4(Kin.Dom.1-N-term.)	82	91	100	95
RSK4(Kin.Dom.2-C-term.)	100	95	100	93
S6K1	83	98	100	95
SBK1	95	96	75	48
SGK	69	100	83	99

SgK110	59	96	100	99
SGK2	70	100	71	71
SGK3	52	98	100	74
SIK	71	98	93	99
SIK2	100	100	100	100
SLK	83	87	99	100
SNARK	100	100	100	93
SNRK	99	77	100	95
SRC	90	100	89	91
SRMS	72	100	87	69
SRPK1	92	100	100	86
SRPK2	100	98	68	64
SRPK3	100	100	100	100
STK16	87	100	100	100
STK33	83	64	97	96
STK35	100	100	100	100
STK36	78	90	97	91
STK39	97	100	83	68
SYK	68	81	96	100
TAK1	94	88	99	43
TAOK1	100	100	96	88
TAOK2	68	74	87	76
TAOK3	100	100	93	84
TBK1	62	100	100	91
TEC	100	100	100	97
TESK1	15	100	97	99
TGFBR1	100	91	89	88
TGFBR2	100	100	100	100
TIE1	86	66	100	64
TIE2	14	100	100	65
TLK1	100	80	100	99
TLK2	39	100	92	100
TNIK	56	94	100	93
TNK1	97	78	100	82
TNK2	99	100	100	100
TNNI3K	100	100	90	69
TRKA	12	67	29	3.4
TRKB	22	96	92	18
TRKC	9.5	79	100	10
TRPM6	88	79	100	98
TSSK1B	100	100	100	92
TSSK3	91	100	100	99
TTK	97	53	98	100
TXK	98	95	94	98
TYK2(JH1domain-cat.)	86	98	100	97
TYK2(JH2domain-pseudok.)	80	79	98	30
TYRO3	86	100	100	100
ULK1	94	100	98	80
ULK2	97	100	98	97
ULK3	96	83	100	100
VEGFR2	59	100	100	80
VPS34	100	87	96	99
VRK2	26	100	94	78
WEE1	85	100	100	100
WEE2	86	100	100	100
WNK1	98	100	99	100
WNK2	75	79	96	81
WNK3	100	90	100	97
WNK4	100	74	88	94
YANK1	28	94	96	67
YANK2	11	100	92	63
YANK3	83	96	100	98
YES	83	100	100	95
YSK1	42	100	86	97
YSK4	18	100	95	55
ZAK	96	100	97	100
ZAP70	95	100	93	85

Supplemental Table S3. Potency of Cmpd **26** against four ISR kinases and two FLT3 isoforms. Compound binding evaluated in cell-free biochemical kinase assays. IC_{50} values determined based on 11-point 3-fold dilution series of **26** ranging from 10 µM to 0.17 nM.

Compound	GCN2	HRI	PERK	PKR	FLT3	FLT3 (D835Y)
**26**	>10 µM	>10 µM	4.7 nM	>10 µM	>10 µM	1.66 µM

4. Materials and Methods

Kinetic Solubility

Verapamil hydrochloride and tamoxifen were obtained from Sigma-Aldrich. All solvents were obtained from commercial sources and used without further purification. Hydrophilic PVDF 96-well filter plates (0.45 mm), 2 mL 96-well assay block, and 96-well UV plates were purchased from Fisher Scientific.Test compound was prepared as a 10 mM stock solution in DMSO. Aqueous suspensions of test compound at 100 µM were prepared in assay buffers. The composition of phosphate buffered saline (PBS) is 0.1 M sodium phosphate, and 0.15 M sodium chloride, adjusted to pH 7.4. The suspensions were agitated at 200 rpm for 1 hour at 25 °C. The suspensions were transferred to a 0.45 µm hydrophilic PVDF 96-well filter plate mounted on a fresh 2 mL 96-well plate and were filtered by centrifugation at 3,000 rpm for 1 min. 150 µL of filtrates were transferred to 96-well UV plate for absorbance measurement. In order to determine the optimal wavelength for detection (λ_{max}), the absorbance spectrum of the DMSO stock solution for each compound was recorded over a broad range of wavelengths (260–450 nm) using a UV/VIS plate reader (Molecular Devices Spectramax i3). To prepare calibration curves, a 1:1 serial dilution was performed on each compound starting at 100 µM to generate calibration solutions with concentrations ranging from 1.25 µM to 100 µM. The UV absorbance of calibrants was measured at λ_{max} for each compound. Concentration of each compound in the assay solution was calculated using the corresponding calibration curve. All assay measurements were performed in duplicate.

Plasma Protein Binding

Pooled mixed gender human donor plasma, Sprague Dawley male rat plasma, CD-1 (ICR) male mouse plasma and male beagle dog plasma were obtained from Bioreclamation Inc. (Westbury, NY). Reusable Teflon base plate, RED Device inserts, and BupH Phosphate Buffered Saline packs (0.1 M sodium phosphate and 0.15 M sodium chloride when dissolved in 0.5 L water) were obtained from Thermo Scientific (Waltham, MA). Propranolol was obtained from Sigma-Aldrich (St. Louis, MO). All solvents were obtained from commercial sources and used without further purification. Frozen plasma was thawed and the pH of plasma and BupH phosphate buffered saline was adjusted to 7.4 prior to dialysis. Into each well of a reusable Teflon 48-well base plate a RED insert was placed open end up. Ten microliters (10 µL) of the test compound (0.1 mM in acetonitrile/water, 1:1 v/v) was added to 990 µL of plasma to achieve 1 µM final concentration. The spiked plasma (200 µL) was added to the sample chamber, which is indicated by the red retainer ring (donor side). To the other side (receiver side), 350 µL of BupH phosphate buffered saline (pH 7.4) was added. A buffer-to-buffer assay was also performed to measure equilibrium across the membrane in the absence of plasma protein. Propranolol was used as a positive control. All samples were prepared in triplicate. The RED device base plate was then sealed and placed in a shaking incubator set to maintain 37°C and 200 rpm for four hours.

After incubation, 100 µL of each sample was combined with an equal volume of either plasma or buffer, to create a similar matrix for all samples. Blank buffer was added to plasma samples, and vice versa. Samples from the incubation, along with the original compound-spiked plasma samples, were prepared for analysis in the same manner. Protein precipitation by addition of two parts of ice-cold acetonitrile to one part of reaction volume was carried out. To ensure complete protein precipitation samples were sealed and placed overnight at 4°C. The next morning samples were centrifuged at 3,600 rpm for 15 minutes. The supernatants (70 µL) were supplemented with an equal volume of internal standard solution (0.15 µM verapamil in water) and subjected to LC-MS/MS analysis.

LC-MS/MS Analysis using the AB Sciex 4500 QTRAP mass spectrometer

Liquid chromatography:
Column: Waters Atlantis T3, 2.1 x 50 mm, 3 µM
Mobile Phase A: Water with 0.1% formic acid
Mobile Phase B: Acetonitrile with 0.1% formic acid
Flow Rate: 0.6 mL/minute

Gradient Program

Time (min)	% A	% B
0.20	95	5.0
1.00	5.0	95
1.99	5.0	95
2.00	95	5.0
3.00	Stop	

Total Run Time: 3.015 minutes
Autosampler: 10 µL injection volume
Autosampler Wash: A: 90% water, 10 % methanol; B: 90% methanol, 10% water

Mass spectrometer:
Instrument: AB SCIEX API 4500 Qtrap
Interface: Turbo Ionspray
Mode: Multiple Reaction Monitoring (Negative ion mode)
Method: 3.015 minute duration

Mass Spectrometer Settings

IS	TEM	CAD	CUR	GS1	GS2
4000	650	Medium	25	70	75

MS/MS Parameters

Compound	Precursor Ion (m/z)	Product Ion (m/z)	Dwell Time (ms)	EP	CE	CXP	DP
Propranolol	260.129	116.1	150	10	25	10	56
Tolbutamide	271.073	90.9	50	10	49	4	76

Data Analysis and Calculations

The data reported is percent plasma protein binding (% PPB), which is obtained from the calculated fraction unbound (f_u):

$$f_u = \frac{compound/IS\ area\ ratio_{\ buffer}}{compound/IS\ area\ ratio_{\ plasma}}$$

$$\%\ \text{PPB} = 100 - (f_u \times 100)$$

P-gp Substrate Assessment using Caco-2 Monolayers

Caco-2 cells (clone C2BBe1) were obtained from American Type Culture Collection (Manassas, VA). Hanks' balanced salt solution, valspodar and lucifer yellow were obtained from Sigma-Aldrich (St. Louis, MO). All solvents were obtained from commercial sources and used without further purification. Cell monolayers were grown to confluence on collagen-coated, microporous membranes in 12-well assay plates. Details of the plates and their certification are shown below. The permeability assay buffer was Hanks' balanced salt solution (HBSS) containing 10 mM HEPES and 15 mM glucose at a pH of 7.4. The buffer in the receiver chamber also contained 1% bovine serum albumin. The dosing solution concentration was 5 μM of test article in the assay buffer +/- 1 μM valspodar. Cells were first pre-incubated for 30 minutes with HBSS containing +/- 1 μM valspodar. Cell monolayers were dosed on the apical side (A-to-B) or basolateral side (B-to-A) and incubated at 37°C with 5% CO_2 in a humidified incubator. Samples were taken from the donor and receiver chambers at 120 minutes. Each determination was performed in duplicate. The flux of lucifer yellow was also measured post-experimentally for each monolayer to ensure no damage was inflicted to the cell monolayers during the flux period. All samples were assayed by LC-MS/MS using electrospray ionization.

LC-MS/MS Analysis using the PE Sciex API 4000 mass spectrometer

Liquid chromatography:
Column: Waters ACQUITY UPLC BEH Phenyl 30 × 2.1 mm, 1.7 µm
Mobile Phase Buffer: 25 mM ammonium formate buffer, pH 3.5
Aqueous Reservoir (A): 90% water, 10% buffer
Organic Reservoir (B): 90% acetonitrile, 10% buffer
Flow Rate: 0.7 mL/minute

Gradient Program

Time (min)	% A	% B
0.00	99	1.0
0.65	1.0	99
0.75	1.0	99
0.80	99	1.0
1.00	99	1.0

Total Run Time: 1.00 minute
Autosampler: 10 µL injection volume
Wash1: water/methanol/2-propanol:1/1/1; with 0.2% formic acid
Wash2: 0.1% formic acid in water

Mass spectrometer:
Instrument: PE SCIEX API 4000
Interface: Turbo Ionspray
Mode: Multiple Reaction Monitoring (Negative ion mode)
Method: 1.0 minute duration

Mass Spectrometer Settings

IS	TEM	CAD	CUR	GS1	GS2
5500	500	7	30	50	5

Data Analysis and Calculations

The apparent permeability (Papp) and percent recovery were calculated as follows:

$$P_{app} = (dC_r/dt) \times V_r/(A \times C_A)$$

$$Percent\ recovery = 100 \times \left(\left(V_r \times C_r^{final}\right) + \left(V_d \times C_d^{final}\right)\right) / (V_d \times C_N)$$

Where,
dC_r/dt is the slope of the cumulative receiver concentration versus time in μM s^{-1};
V_r is the volume of the receiver compartment in cm^3;
V_d is the volume of the donor compartment in cm^3;
A is the area of the insert (1.13 cm^2 for 12-well);
C_A is the average of the nominal dosing concentration and the measured 120 minute donor concentration in μM;
C_N is the nominal concentration of the dosing solution in μM;
C_r^{final} is the cumulative receiver concentration in μM at the end of the incubation period;
C_d^{final} is the concentration of the donor in μM at the end of the incubation period.

Efflux ratio (ER) is defined as Papp (B-to-A) / Papp (A-to-B).

Metabolic Clearance in Hepatocytes

Testosterone was obtained from Cerilliant (Round Rock, TX). 7-Hydroxycoumarin was obtained from Sigma-Aldrich (St. Louis, MO). Ten-donor male pooled cryopreserved human hepatocytes, cryopreserved male Sprague Dawley rat hepatocytes, cryopreserved male ICR/CD-1 mouse hepatocytes, cryopreserved male beagle dog hepatocytes, *In Vitro*Gro HI Medium (Incubation), and *InVitro*Gro HT Medium (Thawing) were obtained from Bioreclamation IVT (Baltimore, MD). All solvents were obtained from commercial sources and used without further purification.Test compound was prepared as a 1 mM stock solution in DMSO. A 2 μM solution of test compound and testosterone was prepared (7-hydroxycoumarin was prepared at 20 μM) in *In Vitro*Gro HI Medium (Incubation). These solutions were placed in a sterile incubator set to maintain 37°C, 5% CO_2, and 98% humidity to pre-warm. Cryopreserved hepatocytes were prepared at a concentration of 2 x 10^6 living cells/mL in incubation media and placed in the incubator to pre-warn. The compound solutions and hepatocyte mixtures were then combined at a ratio of 1:1 (v:v). The final volume of the reaction mixture was 750 μL, containing 1 μM test compound and 1 x 10^6 cells/mL. The reaction mixture was placed in the incubator on a plate shaker. After 0, 15, 30, 60, 90, and 120 minutes of incubation, 100 μL of reaction mixtures were removed from the incubation plate and mixed with 150 μL of ice-cold acetonitrile in a designated well of a 96-well crash plate. The 96-well crash plate was placed on ice for 15 min, and samples were centrifuged (3,600 rpm, 10 min, 4 °C) to precipitate protein. The supernatants were diluted 1:1 (v/v) with water containing tolbutamide, (internal standard for positive and negative modes, respectively) in a 96-well shallow injection plate. This plate was sealed for LC-MS analysis. All measurements were done in duplicate.

LC-MS Analysis

Liquid chromatography:

Column: Waters Atlantis T3, 2.1 × 50 mm x 5 mm
Mobile Phase A: Water with 0.1% formic acid
Mobile Phase B: Acetonitrile with 0.1% formic acid
Flow Rate: 0.7 mL/minute
Gradient Program

Time (min)	% A	% B
0.00	90	10
0.40	90	10
1.20	10	90
2.00	10	90
2.01	90	10
3.00	90	10

Total Run Time: 3 minutes
Autosampler: 10 µL injection volume
Autosampler Wash: A: 90% water, 10 % acetonitrile; B: 90% acetonitrile, 10% water

Mass spectrometer:
Instrument: AB SCIEX API 3200
Interface: Turbo Ionspray
Mode: Q1
Method: 3.0 minute duration

Mass Spectrometer Settings

IS	TEM	CUR
-4500	500	25

MS Parameters

Compound	Polarity	Q_1 Mass	Dwell Time	DP	EP
Testosterone	+	289	150	70	10
7-Hydroxycoumarin	+	163.2	150	70	10
Tolbutamide	-	269.3	150	-70	10

Data Analysis and Calculations

Calculation of *in vivo* hepatic clearance

In vivo hepatic clearance CL_H was calculated using the well stirred liver model according to the following equation:

$$CL_H = \frac{Q_H \cdot f_u \cdot CL'_{int}}{Q_H + f_u \cdot CL'_{int}}$$

where Q_H is the total liver blood flow, f_u is unbound fraction of the drug, and CL'_{int} is defined as follows:

$$CL'_{int} = CL_{int} \times (10^6 \text{ cells/g of liver weight}) \times (\text{g liver weight/kg of body weight})$$

In the first approximation, used in this study, $f_u = 1$.

Hepatic extraction ratio E_H was calculated using the following equation:

$$E_H = \frac{CL_H}{Q_H}$$

Physiological Parameters of Mammalian Species Used for Calculation of Hepatic Clearance:

Species	g liver wt/kg body wt	10^6 cells/g liver wt	Q_H, mL/min/kg body wt
Human	26	99	21
Rat	37	128	68
Dog	33	188	31
Mouse	55	128	120

PERK Crystallization and Structure Determination

Human PERK (575-1094 Δ670-874) was purified as described previously (2). Purified PERK protein at 11.3 mg/ml was mixed with 10 mM **26** (in DMSO) to a final protein-inhibitor molar ratio 1:2. The PERK-**26** mixture was incubated on ice for 2 hours. The crystal for the data collection was grown at 20 °C in a sitting drop by combining 2.0 µl PERK1/ **26** mixture, 2.0 µl reservoir solution (12% PEG3350, 4% tacsimate pH7.0), and 0.4 µl seed stock which was equilibrated over a 500 µl reservoir solution. The crystal was grown to 0.2 x 0.2 mm over a three-week period before harvesting for analysis. The crystal was transferred stepwise to a cryo-solution with 12% PEG3350, 4% tacsimate, pH7.0, 250mM NaCl, 20% glycerol before being frozen in liquid nitrogen.

Diffraction data was collected at the GMCA-CAT beamline 23IDD at the Advanced Photon Source at Argonne National Laboratory using a Pilatus 6M detector. The data set was collected from a single crystal at 1.03322Å (12000 eV) wavelength for 180° in 900 images. The diffraction images were processed with DIALS (3) and scaled with AIMLESS (4). The structure was solved by molecular replacement using PDB 4X7J (5) as starting model by Phaser (6). The structure was manually built using Coot (7) and subsequently refined using Refmac5 (8). The crystallographic figures were generated by CCP4MG (9), and the statistics of data collection and refinement are summarized in Supplemental Table S1.

Cell lines

MV-4-11 cells (ATCC CRL-9591) were grown in Iscove's Modified Dulbecco's Medium (Gibco 12440-053) with 10% Dialyzed FBS (Gibco 26400-044). EoL-1 cells (ECACC 94042252) were grown in RPMI (Gibco 11875-093) with 10% Dialyzed FBS (Gibco 26400-044).

In vitro kinase activity assay

Flt3 (h) and Flt3(D835Y) are incubated with 8 mM MOPS pH 7.0, 0.2 mM EDTA, 50 µM EAIYAAPFAKKK, 10 mM Magnesium acetate and [γ-33P]-ATP (specific activity and concentration as required). The reaction is initiated by the addition of the Mg/ATP mix. After incubation for 40 minutes at room temperature, the reaction is stopped by the addition of phosphoric acid to a concentration of 0.5%. An aliquot of the reaction is then spotted onto a filter and washed four times for 4 minutes in 0.425% phosphoric acid and once in methanol prior to drying and scintillation counting.

Cell viability assay

MV-4-11 and EoL-1 were plated at a density 20,000 cells/well in a 96 well plate. Cells were incubated with DMSO, 1 µM docetaxel, or an 8-pt, 2-fold dose response of **26** in quadruplicate. On day 3 and day 7, cells from DMSO-treated wells were counted and cells from all wells were split back so that the DMSO-treated wells matched the original seeding density. Cells were centrifuged in 96 well filter plates (Sigma) and resuspended in fresh compound-containing media. On day 10, cells were incubated with alamarBlue™ HS Cell Viability Reagent (Invitogen) (10 µL of alamarBlue in a well of 90 µL media) for 2 hours at 37C/5% CO_2. Fluorescence was measured on the EnVision Plate reader (Perkin Elmer) at an ex/em of 560/590 nm. Raw fluorescence values were normalized to positive and negative controls (DMSO, 1 µM docetaxel) to get a % viability. Replicates were averaged and dose responses were plotted on a 4 Parameter Logistic Model using XLfit.

Western blot analysis

MV-4-11 cells were plated in 6 well plates overnight in reduced serum media (IMDM + 0.5% FBS). The following day, compound or vehicle were added to the plate for a final DMSO concentration of 0.1% and incubated at 37 C for 2 hours. Cells were washed with 1X PBS (Gibco 10010-023), and lysed in RIPA buffer with added inhibitors. Protein concentration was determined using a BCA assay (Thermo Scientific 23225). Lysate was then combined with Bolt LDS Sample Buffer and reducing agent (Thermo Fisher Scientific) - Samples were heated 70°C in the heat block for 10 minutes and loaded onto 12 well Bolt Bis-Tris Plus gels with 23 µg total protein and 2.5 µl of LiCOR Chameleon Duo Pre-stained ladder. P-STAT5 used 10% Bolt gels and p-ERK was run on 12% Bolt gels. Gels were run for 32 minutes at 200V in 1X Bolt MOPS Running Buffer (Invitrogen). Gels were transferred onto a nitrocellulose membrane (Invitrogen) using the Invitrogen Mini Blot Module and 1X Bolt Transfer Buffer with 10% methanol (Invitrogen) at 10V for 120 minutes. Membranes were stained for total protein normalization using the Revert 700 Total Protein Stain Kit (LiCOR). Membranes were blocked for 1 hour at room temperature with Intercept® (TBS) Blocking Buffer (LiCOR 927-60001) and then an overnight incubation at 4 C with primary antibody. The following day, membranes are washed 3 times with 1X TBS and incubated with secondary antibody for 1 hour at room temperature. Membranes are washed again and imaged on the LiCOR Odyssey CLx. The primary antibodies used were p-STAT5 and p-ERK from Cell Signaling. Other compounds used were Crenolanib and Gilteritinib from Medchemexpress, and Quizartinib from Tocris.

5. References

1. Williams CJ, Headd JJ, Moriarty NW, Prisant MG, Videau LL, Deis LN, et al. MolProbity: More and better reference data for improved all-atom structure validation. Protein Sci. 2018;27(1):293-315.
2. Calvo V, Surguladze D, Li AH, Surman MD, Malibhatla S, Bandaru M, et al. Discovery of 2-amino-3-amido-5-aryl-pyridines as highly potent, orally bioavailable, and efficacious PERK kinase inhibitors. Bioorg Med Chem Lett. 2021;43:128058.
3. Winter G, Waterman DG, Parkhurst JM, Brewster AS, Gildea RJ, Gerstel M, et al. DIALS: implementation and evaluation of a new integration package. Acta Crystallogr D Struct Biol. 2018;74(Pt 2):85-97.
4. Evans PR, Murshudov GN. How good are my data and what is the resolution? Acta Crystallogr D Biol Crystallogr. 2013;69(Pt 7):1204-14.
5. Smith AL, Andrews KL, Beckmann H, Bellon SF, Beltran PJ, Booker S, et al. Discovery of 1H-pyrazol-3(2H)-ones as potent and selective inhibitors of protein kinase R-like endoplasmic reticulum kinase (PERK). J Med Chem. 2015;58(3):1426-41.
6. McCoy AJ, Grosse-Kunstleve RW, Adams PD, Winn MD, Storoni LC, Read RJ. Phaser crystallographic software. J Appl Crystallogr. 2007;40(Pt 4):658-74.
7. Emsley P, Lohkamp B, Scott WG, Cowtan K. Features and development of Coot. Acta Crystallogr D Biol Crystallogr. 2010;66(Pt 4):486-501.
8. Murshudov GN, Skubak P, Lebedev AA, Pannu NS, Steiner RA, Nicholls RA, et al. REFMAC5 for the refinement of macromolecular crystal structures. Acta Crystallogr D Biol Crystallogr. 2011;67(Pt 4):355-67.
9. McNicholas S, Potterton E, Wilson KS, Noble ME. Presenting your structures: the CCP4mg molecular-graphics software. Acta Crystallogr D Biol Crystallogr. 2011;67(Pt 4):386-94.
